# A pH‐Responsive Synthetic Receptor for Switchable Binding of Carbohydrates

**DOI:** 10.1002/cplu.202500447

**Published:** 2025-10-23

**Authors:** Francesco Milanesi, Giona Corti, Andrea Baldi, Stefano Roelens, Oscar Francesconi

**Affiliations:** ^1^ Department of Chemistry “Ugo Schiff” DICUS Università degli Studi di Firenze Via della Lastruccia 13 I‐50019 Sesto Fiorentino Firenze Italy; ^2^ Consorzio Interuniversitario Nazionale per la Scienza e Tecnologia dei Materiali (INSTM) 50121 Firenze Italy

**Keywords:** aggregation, carbohydrates, molecular recognition, molecular switch, synthetic receptor

## Abstract

Stimuli‐responsive, water‐soluble synthetic receptors are key to advancing dynamic molecular recognition in aqueous environments, with implications for self‐assembly, molecular machines, and biomedical systems. Herein, a macrocyclic receptor is reported that exhibits pH‐dependent binding properties toward saccharides in water, in that it displays markedly different affinities between alkaline and neutral conditions. Spectroscopic and binding studies reveal that the degree of protonation of the solubilizing groups modulates the receptor self‐association phenomena, together with concomitant substantial loss of binding ability. This work highlights a rare example of a pH‐switchable carbohydrate receptor operating in water and underscores the potentials of such system in the design of smart, responsive molecular architectures.

## Graphical abstract

A diaminocarbazole macrocyclic receptor, designed for disaccharide recognition in water, exhibits distinct binding properties under alkaline versus neutral pH conditions. Carbohydrates are selectively recognized under alkaline conditions, whereas release of the bound guest occurs at physiological pH, effectively behaving as a pH‐switchable carbohydrate receptor. Such a stimuli‐responsive behavior holds promise for applications in sensing, molecular recognition, and smart material design.

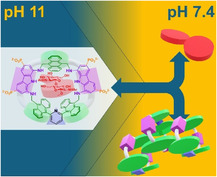

## 1 Introduction

Molecular recognition of carbohydrates, typically mediated by specialized proteins in aqueous environments, underpins a wide range of biological processes, both physiological and pathological. These include cell–cell recognition and adhesion, host–pathogen interactions, immune system modulation, and the promotion of inflammatory responses.^[^ ^1^ ^]^ Synthetic receptors selectively recognizing carbohydrates in water may provide new tools for chemical biology and biomedical science, potentially modulating protein‐carbohydrate interactions.^[^ ^2^ ^]^ However, effective recognition of highly hydrophilic molecules, such as carbohydrates in water, by synthetic receptors remains challenging, especially when their design follows a biomimetic approach that relies exclusively on noncovalent interactions, such as hydrogen bonding.^[^ ^3^ ^,^ ^4^ ^]^ In water, these interactions are often weakened due to competition from solvent molecules, making selective and strong binding particularly difficult to achieve.^[^ ^5^ ^,^ ^6^ ^]^

Supramolecular chemists have tackled this challenge by designing endo‐functionalized cavities, which involves embedding hydrogen‐bonding sites within deep hydrophobic cavities.^[^ ^7^, ^8^ ^–^ ^9^ ^]^ These architectures include temples,^[^ ^10^ ^]^ cages,^[^ ^11^ ^]^ macrocycles,^[^ ^12^ ^]^ and molecular tweezers.^[^ ^13^ ^]^ These systems have demonstrated effective and selective recognition in water, not only of simple monosaccharides,^[^ ^14^, ^15^ ^–^ ^16^ ^]^ but also of more complex and challenging carbohydrates, such as disaccharides,^[^ ^17^ ^,^ ^18^ ^]^ oligosaccharides,^[^ ^19^ ^,^ ^20^ ^]^ and glycans.^[^ ^21^ ^,^ ^22^ ^]^ Major advances have opened the way to applications in biologically relevant contexts, including sensing,^[^ ^23^ ^]^ diagnostics,^[^ ^24^ ^]^ and modulation of carbohydrate‐mediated biological processes.^[^ ^25^ ^,^ ^26^ ^]^

Notably, most of reported synthetic receptors are not stimuli responsive. Incorporating responsiveness to external triggers, such as pH, light, or redox triggers, could significantly expand their scope in molecular recognition.^[^ ^27^, ^28^ ^–^ ^29^ ^]^ This feature would enable the development of switchable noncovalent bioconjugation strategies, smart drug delivery systems, water‐soluble molecular machines, and recyclable host‐based adsorption materials.

**Figure 1:**
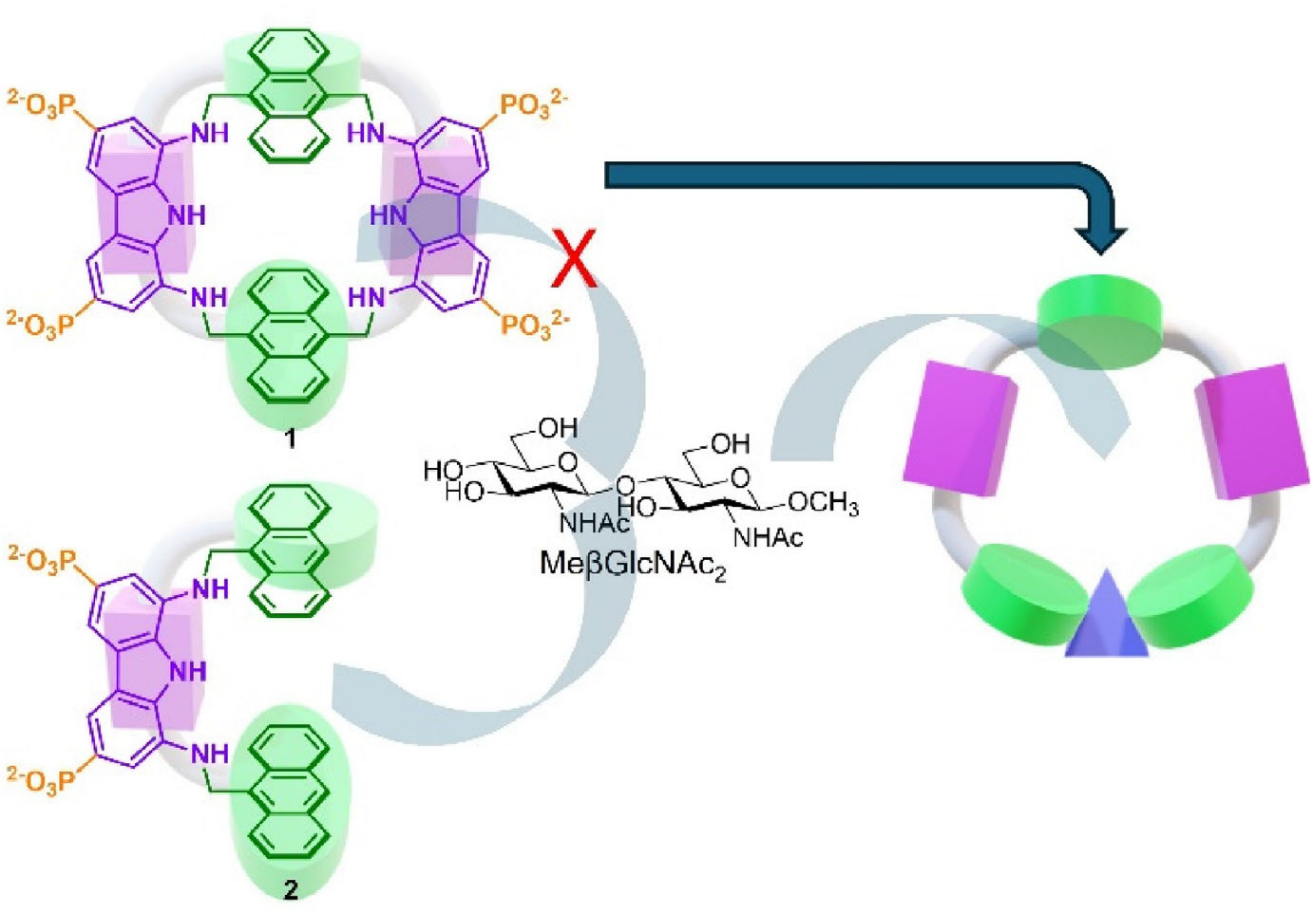
Schematic representation of the strategy followed to develop a larger cavity macrocyclic receptor to accommodate disaccharide guests, such as meβGlcNAc_2_.

In recent years, we have developed a family of synthetic receptors featuring diaminocarbazole units as hydrogen‐bonding motifs,^[^ ^30^ ^]^ and phosphonate groups as water solubilizing groups. These receptors featuring both, macrocyclic and tweezers‐shaped architectures, have demonstrated effective carbohydrate recognition in water.^[^ ^16^ ^,^ ^17^ ^,^ ^31^ ^]^ The progenitor of this family is macrocycle **1** (**Figure** **1**), which incorporates two diaminocarbazole units for hydrogen bonding, alternating with two anthracene moieties, to promote CH–*π* interactions.^[^ ^16^ ^]^ Receptor **1** exhibits significant affinity and selectivity toward monosaccharides in water; however, the size and rigidity of the macrocyclic architecture proved unsuitable to accommodate larger guests, such as disaccharides. Indeed, recognition of 1 → 4 linked disaccharides, such as the methyl glycosides of cellobiose (MeβCeB), maltose (MeβMal), and lactose (MeβLac), was restricted to binding to the methyl‐β‐glucoside unit exclusively, resulting in lack of selectivity. Furthermore, larger disaccharides, such as the methyl β glycoside of *N*,*N′*‐diacetylchitobiose (MeβGlcNAc_2_), were not recognized at all. To open the binding cavity of the macrocyclic structure, one diaminocarbazole unit was removed, yielding the tweezers‐shaped receptor **2**.^[^ ^17^ ^]^ Despite the loss of hydrogen bonding groups, this open chain architecture gave selective recognition of 1 → 4 linked disaccharides, exhibiting a remarkable binding affinity of 160 μM for Me*β*GlcNAc_2_.

In addition to their recognition properties, diaminocarbazole‐based receptors bearing phosphonate solubilizing groups exhibit different degrees of self‐association in water at neutral pH. This behavior is primarily driven by hydrophobic interactions between extended aromatic surfaces, further stabilized by hydrogen bonding involving partially protonated phosphonate groups. Notably, many of these systems display a pH‐dependent behavior, as evidenced by ^1^H‐NMR experiments. At alkaline pH, spectra typically show sharp, well resolved signals. In contrast, at neutral pH, self‐association leads to pronounced signal broadening, significant chemical shift variations, or the emergence of new set of signals. Interestingly, despite these differences, the overall binding abilities toward carbohydrates are generally conserved.

Here we present the synthesis of a new member of the diaminocarbazole receptor family featuring a larger macrocyclic structure with respect to progenitor **1** (Figure 1), designed to accommodate disaccharide structures, including MeβGlcNAc_2_. In contrast to its progenitor, the new receptor shows a pH‐dependance dramatically affecting the recognition properties, in that it shows on–off switching of saccharide binding. This makes it a rare example of a stimuli‐responsive receptor for biomimetic recognition of carbohydrates, which can be used to induce binding and release of disaccharides as a function of the solution pH.

## 2 Results and Discussion

**Scheme 1:**
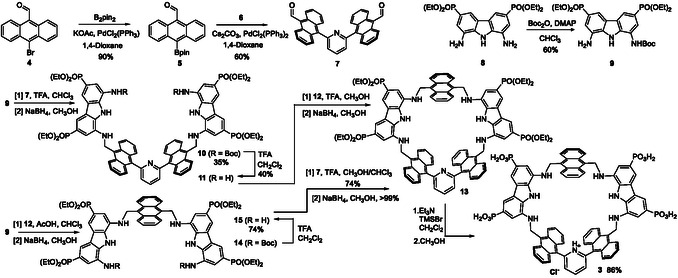
Schematic pathways for the synthesis of building blocks **7**, **9** and their alternative assembling sequences with **12** to give receptor **3**. TFA = trifluoroacetic acid; B_2_pin_2_ = bis(pinacolato)diboron; Boc = *tert*‐Butyloxycarbonyl; TMSBr = bromotrimethylsilane.

Receptor **3** (**Scheme** **1**) was designed to combine the structural preorganization of the macrocyclic receptor **1** with the enhanced binding site accessibility of receptor **2**. To this end, the macrocyclic structure of receptor **1** was expanded by replacing one of the two anthracene units with a 2,6‐dianthracenylpyridine moiety. This new building block introduces two convergent anthracene units, providing additional hydrophobic surface to match the extended hydrophobic backbone of all equatorially substituted 1 → 4 linked disaccharides, such as cellobiose and GlcNAc_2_. Furthermore, the inward‐oriented nitrogen atom of the pyridine ring can provide additional hydrogen bonding interactions, stabilizing guest binding within the cavity. The macrocyclic structure of receptor **3** is designed to increase the distance between both the anthracene and the carbazole units, with respect to **1**.

Due to the lower symmetry of receptor **3**, the synthesis was approached via two distinct strategies, differing in the order of building blocks assembly. In the first strategy, the two diaminocarbazole units were first linked to the 2,6‐dianthracenylpyridine moiety, followed by ring‐closing with a third anthracene unit. In the alternative route, two mono‐protected diaminocarbazole units were assembled with a single anthracene unit and subsequently ring‐closed using the 2,6‐dianthracenylpyridine building block. Both synthetic pathways were explored to optimize yields and macrocycle formation.

In the first approach, boronate **5** was obtained in 90% yield by a Miyaura borylation of 10‐bromoanthracene‐9‐carbaldehyde (**4**), prepared according to a reported procedure.^[^ ^32^ ^]^ The boronate ester **5** was subsequently used for a Suzuki coupling with 2,6‐dibromopyridine **6,** to afford the dialdehyde **7** in 60% yield. The tetraethyl diphosphonate ester of the diaminocarbazole unit **8**, prepared as previously described,^[^ ^16^ ^]^ was monoprotected using Boc_2_O in CHCl_3_ under DMAP catalysis to give **9** in 60% yield. Dialdehyde **7** and monoamine **9** were then condensed in CHCl_3_ using trifluoroacetic acid (TFA) as catalyst to form the corresponding imine, which was reduced with NaBH_4_ in CH_3_OH to yield amine **10** (35% over two steps). Deprotection with TFA in CH_2_Cl_2_ at 0 °C afforded **11** in 40% yield. Macrocyclization was then attempted via condensation with 9,10‐diformylanthracene **12** in MeOH under TFA catalysis, followed by NaBH_4_ reduction. However, only traces of the desired macrocycle **13** were obtained. The low yield observed in the final step was attributed to the poor stability of the imine macrocycle during the reduction, which led to the partial hydrolysis of the imines and formation of a complex mixture, most likely consisting of oligomeric byproducts. The formation of the macrocyclic structure, albeit in trace amounts, validated the design but prompted the development of an alternative synthetic route.

In the alternative strategy, the monoprotected diaminocarbazole **9** was condensed with 9,10‐diformylanthracene **12** in refluxing CHCl_3_ under acetic acid catalysis to afford the corresponding imine, which was then reduced with NaBH_4_ in MeOH to yield the amine **14**. The crude was used without further purification in the deprotection step, using TFA in CH_2_Cl_2_ at 0 °C, to afford the intermediate **15** in 74% yield over three steps. Macrocyclization was then performed by condensation with dialdehyde **7** in CH_3_OH/CHCl_3_ mixture with TFA catalysis under reflux, yielding the corresponding imine in 74% yield. Reduction with NaBH_4_ in CH_3_OH gave the lipophilic receptor **13** in quantitative yield. The final hydrolysis of the phosphonate esters was achieved using trimethylbromosilane, followed by treatment with CH_3_OH, to give receptor **3** in acidic form with 86% yield over two steps. The route described enabled the preparation of receptor **3** in only six steps, starting from readily available building blocks, with an overall yield of 45%.

Analogous to its progenitor, receptor **3** was freely soluble in water over a wide range of pH, from physiological (pH 7.4) to alkaline (pH 11), whereas it precipitated under acidic conditions, likely due to extensive protonation of the phosphonate groups. The pH‐dependent behavior of receptor **3** was further investigated by ^1^H‐NMR spectroscopy (Figure S22, Supporting Information). Spectra recorded at different pH values revealed differences between alkaline and neutral conditions. At pH 11, where phosphonate groups are fully deprotonated, the spectrum showed a single set of sharp signals. In contrast, at pH 7.5, partial protonation led to the appearance of multiple broad signals, suggesting the onset of strong self‐association. Dilution experiments carried out at pH 11, revealed appreciable chemical shift variations only at concentrations above 1 mM (Figure S28, Supporting Information). The data fit a self‐association model involving trimeric and hexameric species, with weak self‐association constants: log*β*_trim_ = 4.15 ± 0.07 and log*β*_hexa_ = 10.57 ± 0.08. These low values indicate that self‐association is negligible within the concentration range used for binding measurements, where the monomer remains the predominant species.

**Scheme 2:**
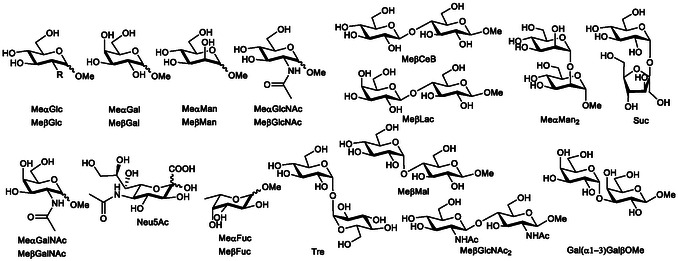
Structure of the investigated saccharides and their abbreviations.

A preliminary ^1^H NMR screening has been carried out to find the most interesting guests with potential good affinities for further quantitative investigation (Figure S23‐S24, Supporting Information). Binding ability of **3** was qualitatively assessed by monitoring the chemical shift variations and line broadening of carbohydrate proton signals upon addition of an equimolar amount of the receptor. The experiments were performed at pH 11 toward a set of monosaccharides (**Scheme** **2**), including sialic acid (Neu5Ac) and the α and β methyl glycosides of glucose (Glc), galactose (Gal), mannose (Man), N‐acetyl glucosamine (GlcNAc), N‐acetyl galactosamine (GalNAc), and fucose (Fuc). Additionally, a series of disaccharides (Scheme 2) with diverse glycosidic linkages were tested: 1 → 1 linked disaccharides such as sucrose (Suc) and trehalose (Tre); the 1 → 2 linked mannobioside (Me*α*Man_2_); 1 → 3 linked Gal(α1–3)GalβOMe; and 1 → 4 linked disaccharides, including MeβCeB, MeβMal, MeβLac, and MeβGlcNAc_2_. Little or no chemical shift changes (Δ*δ* < 0.05 ppm) were observed for monosaccharides MeαMan, MeβGlcNAc, MeαGalNAc, MeβGalNAc, and Neu5Ac and disaccharides Suc, Tre, and MeαMan_2_, indicating a very weak or negligible binding with receptor **3**. A moderate chemical shift change (0.05 < Δ*δ* < 0.2 ppm) was observed for all the other investigated monosaccharides as well as for the 1→3 linked disaccharide Gal(*α*1–3)GalβOMe, suggesting a weak to moderate binding interaction. In contrast, a marked upfield shift (Δ*δ* > 0.3 ppm) for most of the carbohydrate signals, particularly evident for the anomeric protons, was detected for all the 1 → 4 linked disaccharides investigated, pointing to a selective binding preference, analogous to that previously reported for the tweezers‐shaped receptor **2**. Additionally, significant line broadening of proton signals was observed for all the 1 → 4 linked disaccharides, providing evidence of slow chemical exchange, typically associated to strong binding,. This effect was particularly prominent for the all‐equatorial disaccharides MeβCeB and MeβGlcNAc_2_. Results were summarized in Table S1 in Supporting Information.

**Figure 2:**
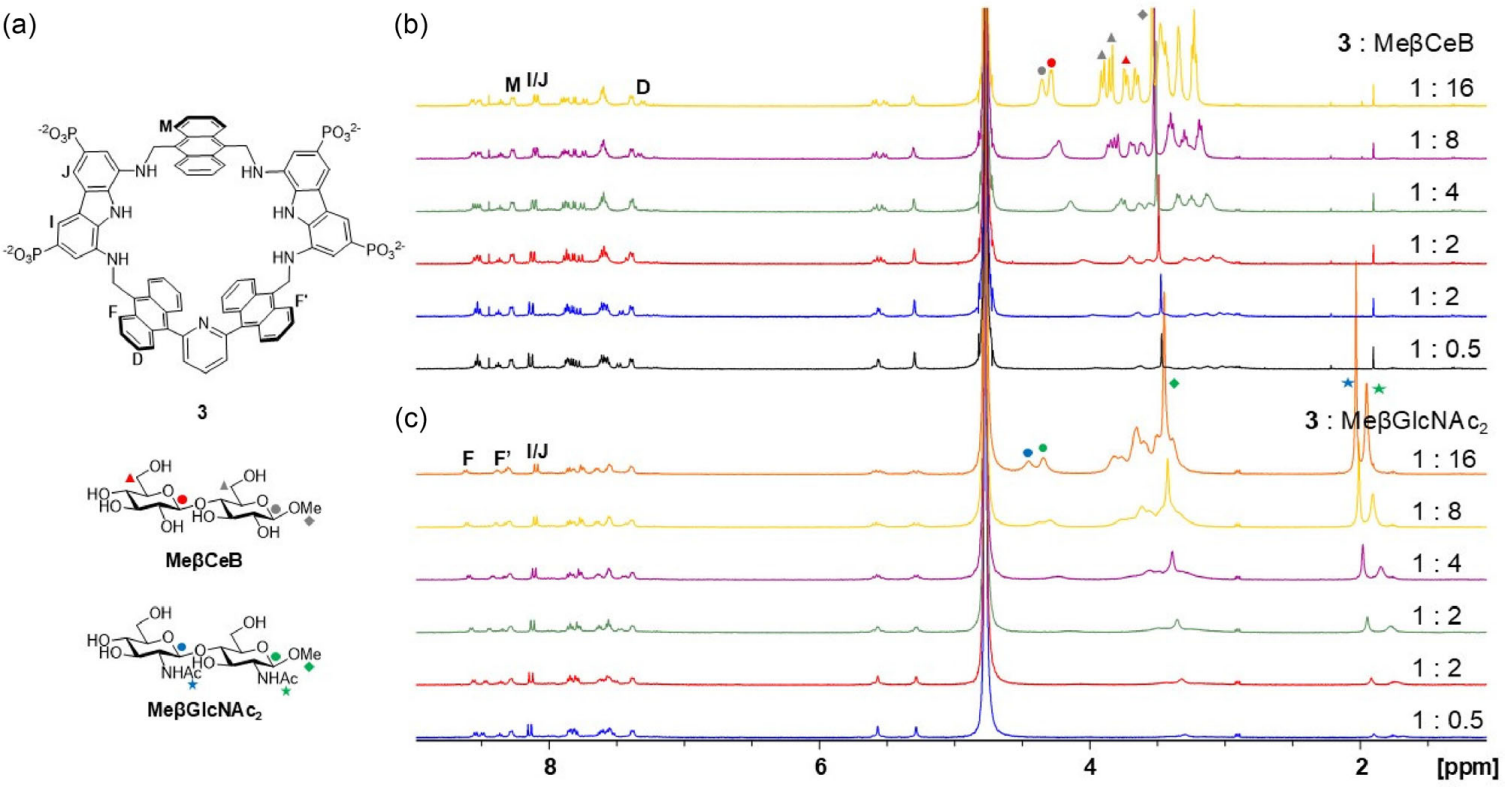
Selected spectra from the ^1^H‐NMR spectroscopic titrations (500 MHz, D_2_O, pD11, 298 K) of receptor **3** b) 0.410 mM; c) 0.461 mM with MeβCeB (b) and MeβGlcNAc_2_ (c). Proton signals followed for binding analysis are labeled in (b) and (c) as shown in panel a): receptor **3** signals are indicated by letters and MeβCeB and MeβGlcNAc_2_ signals are represented by color‐coded symbols.

A quantitative investigation was then carried out by ^1^H NMR titrations in D_2_O at pH 11 toward the most promising guests: MeβCeB and MeβGlcNAc_2_ (**Figure** **2**). For both disaccharides, the binding data were well‐described by a simple 1:1 association model with receptor **3**, resulting in association constants of log*β*_1:1_ = 2.96 ± 0.01 for MeβCeB and log*β*_1:1_ = 3.10 ± 0.02 for MeβGlcNAc_2_. These values correspond to dissociation constants (*K*_d_) of 1.09 mM and 0.80 mM, respectively. In contrast to receptor **1**, receptor **3** shows the ability to selectively recognize disaccharides and to accommodate larger guests, such as MeβGlcNAc_2_, within its binding pocket. Indeed, like the open‐chain receptor **2**, receptor **3** exhibits distinct selectivity toward MeβGlcNAc_2_, albeit with somewhat lower binding affinity. The millimolar binding affinity for MeβGlcNAc_2_ was further confirmed by ITC in H_2_O at pH 11 resulting in a log*β*_1:1_ = 2.79 ± 0.01 corresponding to a dissociation constant (*K*_d_) of 1.64 mM (Supporting Information, p. 55–59).

**Figure 3:**
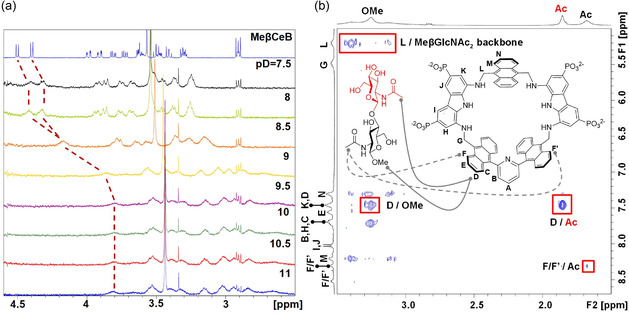
NMR binding studies. a) ^1^H NMR spectra (500 MHz, D_2_O, 298 K) of **3** (1 mM) in presence of MeβCeB (1 mM) at different pD values. Reference spectrum of MeβCeB at pD 7.5 on top. Dotted lines show variation in chemical shifts for the anomeric protons. b) NOESY (500 ms mixing time, 500 MHz, D_2_O, pD 11, 298 K) spectrum of **3** (1 mM) and MeβGlcNAc_2_ (3 mM). NOE contacts are indicated as solid gray lines; alternative NOE contacts are indicated as dashed gray lines; intermolecular cross peaks are indicated in red squares.

Surprisingly, when ^1^H‐NMR binding studies were performed to pH 7.4, no significant changes were observed in the proton signals of either MeβCeB or MeβGlcNAc_2_ upon addition of receptor **3**, indicating substantial loss of binding under neutral conditions. This behavior was also observed for monosaccharides (Figure S25, Supporting Information). A stepwise pH variation experiment from pH 11–7.5 was carried out on an equimolar solution of **3** and disaccharide, either MeβCeB (**Figure** **3**a) or MeβGlcNAc_2_ (Figure S26, Supporting Information). The results revealed that the ^1^H‐NMR signals of both, host and guest, remained relatively unperturbed down to pH 9. Below this threshold, between pH 9 and pH 8, the sharp signals of the receptor progressively turned into multiple broad signals. Correspondingly, the proton signals of the disaccharide gradually shifted toward the chemical shifts of the unbound species. This observation indicates that no significant binding occurs below pH 8. This was further confirmed by titration experiments with MeβCeB and MeβGlcNAc_2_ at pD 7.4 (Figures S30 and S32, Supporting Information), where no significant changes in the chemical shifts of the saccharides were observed throughout the experiment. Additionally, ITC measurements at pH 7.4 showed little or no enthalpy changes upon addition of MeβGlcNAc_2_ to receptor **3** (Figure S33, Supporting Information), supporting the loss of binding ability at neutral pH observed by NMR. As previously observed with other members of the diaminocarbazole receptors family bearing phosphonate solubilizing groups, the pH range below 8 induces changes in self‐association behavior, likely due to partial protonation of the phosphonate groups that stabilize the associated structure. However, in contrast to previously studied systems, self‐association appears to show concomitant loss of carbohydrate recognition. It is reasonable to assume that the pyridine moiety may be responsible for the changes in the receptor's binding properties at neutral pH, either through hydrogen bonding to partially protonated phosphonate groups, or through the occurrence of protonation equilibria. Unfortunately, strong receptor signal overlap and pronounced line broadening prevented a detailed description of the self‐associated species by NMR. Nevertheless, the millimolar binding affinity observed above pH 9, which is nearly lost below pH 8 but fully restored under alkaline conditions (Figure S27, Supporting Information), makes receptor **3** a pH‐responsive molecular switch for saccharide recognition.

To gain insight into the origin of the binding properties, a 3D description of the complex between receptor **3** and MeβGlcNAc_2_ was attempted at pH 11 by combining NMR data with molecular modeling calculations. Nuclear overhauser effect spectroscopy (NOESY) spectra (Figure 3b) of a mixture of receptor **3** and MeβGlcNAc_2_ revealed several nuclear overhauser effect (NOE) contacts; however, only few could be assigned unambiguously due to significant signal overlap; specifically, those involving the saccharide methyl groups and the anthracene protons within the 2,6‐dianthracenylpyridine moiety. One NOE contact was observed between the methyl protons of the *N*‐acetyl group on the methyl glycosidic unit and one of the diastereotopic protons H‐F or H‐F’ of the receptor, which became nonequivalent upon binding due to the chiral environment in the complex. Two additional NOE contacts were identified between the H‐D protons of the receptor and the methyl protons of the second *N*‐acetyl group, as well as with the methyl aglycone. Furthermore, a series of NOE interactions were found between the methylene protons L and various aliphatic protons of the disaccharide. These contacts were unexpected, because methylene protons were expected to point outwards from the cavity, therefore not in close proximity to the saccharide.

**Figure 4:**
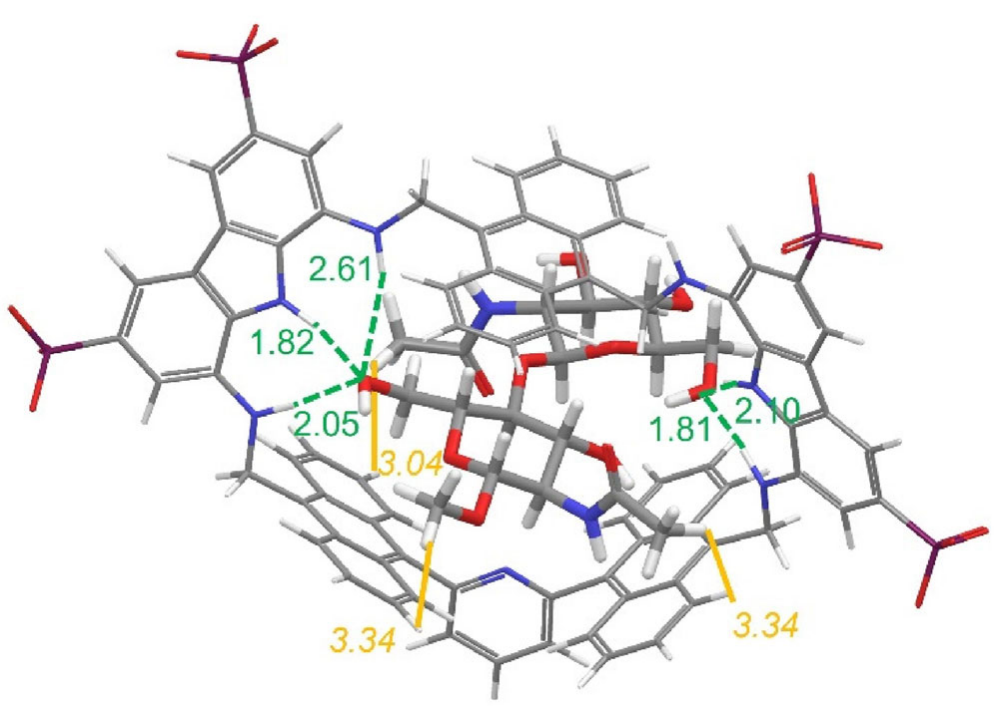
Global minimum structure of the **3‐**meβGlcNAc_2_ complex. The strongest intermolecular NOEs found between **3** and MeβGlcNAc_2_ are indicated as solid yellow lines, with corresponding distances [Å] calculated from the lowest energy conformer. Intermolecular hydrogen‐bonding interactions found in the calculated structure are indicated as dashed green lines with corresponding oxygen/hydrogen distances [Å].

A conformational search was run on the 1:1 host–guest complex using the tetraphosphonate anionic receptor at alkaline pH. The calculation yielded a family of minimum energy structures consistent with the NOE data (**Figure** **4** and S35, Supporting Information). Notably, the macrocyclic structure of receptor **3** slightly collapses upon complexation with MeβGlcNAc_2_, adopting a binding conformation reminiscent of that observed for receptor **2**, in which two opposing anthracene units squeeze over the disaccharide bound within the cleft. This binding conformation is achieved through rotation of one of the methylene groups, which reorients the H‐L protons inward, in agreement with the observed NOE contacts. Based on the above model, all O···H interatomic distances shorter than the sum of the van der Waals radii and consistent with hydrogen bonding criteria were calculated. Five hydrogen bonding interactions were found between **3** and MeβGlcNAc_2_ involving both the carbazole units and the OH‐6 of each saccharide unit. Although receptor **3** establishes a larger number of hydrogen bonding interactions with MeβGlcNAc_2_ compared to receptor **2**, the smaller binding affinity is most likely attributed to the energetic cost associated with receptor folding upon complexation.

## 3 Conclusion

This study presents compound **3** as a pH‐responsive synthetic receptor selectively recognizing 1 → 4 linked disaccharides under alkaline conditions. Receptor selectivity toward all‐equatorial disaccharides, such as MeβCeB and MeβGlcNAc_2_, reveals a binding profile reminiscent of the previously reported tweezers‐shaped receptor **2**, although both selectivity and affinity for MeβGlcNAc_2_ are slightly less pronounced. Quite interestingly, the most significant novelty with respect to previous receptors stems from the remarkable loss of binding ability at physiological pH. Indeed, under the concentration conditions used for binding studies (1 mM), receptor **3** is predominantly monomeric at pH above 9, whereas below pH 8 self‐association is accompanied by a concomitant substantial loss of binding ability, in contrast to previously studied diaminocarbazole‐based receptors, for which self‐association did not affect guest recognition. This unique feature makes receptor **3** a remarkable pH‐sensitive molecular tool for on–off switching of saccharide recognition. Molecular modeling, supported by NMR data under alkaline conditions, suggests that in the 1:1 complex between **3** and MeβGlcNAc_2_, the receptor adopts a slightly collapsed conformation, squeezing the disaccharide into the binding cavity. Overall, receptor **3** offers a promising scaffold for the development of stimuli‐responsive carbohydrate receptors, with potential applications in sensing, molecular recognition, and smart material design.

## Supporting Information

The authors have cited additional references within the Supporting Information.^[^ ^33^ ^,^ ^34^ ^]^

## Conflict of Interest

The authors declare no conflict of interest.

## Supporting information

Supplementary Material

## Data Availability

The data that support the findings of this study are available in the supplementary material of this article.
