# Supplementary material for: A pH‐Responsive Synthetic Receptor for Switchable Binding of Carbohydrates
Source: Chempluschem. 2025 Oct 23;90(12):e202500447. doi: 10.1002/cplu.202500447 (PMC12701286; doi:10.1002/cplu.202500447)
Supplement: Supplementary file 1 — Supplementary Material [file CPLU-90-e202500447-s001.pdf]

SUPPORTING INFORMATION

**Synthesis and characterization of chemical materials**	**p. S2**
**General**	**p. S2**
**Materials**	**p. S2**
**NMR Spectra**	**p. S10**
**Binding studies**	**p. S31**
**NMR investigation of the effect of pH on the ^{1}H-NMR spectra of receptor 3**	**p. S31**
**NMR preliminary screening**	**p. S32**
**NMR investigation of the effect of pH on binding**	**p. S37**
**NMR titrations and data analysis**	**p. S39**
**Calorimetric titrations and data analysis.**	**p. S55**
**Structural studies**	**p. S61**
**NMR studies**	**p. S61**
**In Silico Docking Study**	**p. S62**

Synthesis and characterization of chemical materials.

General. ESI-MS analyses were performed in positive and negative ion mode and were recorded on an LCQ-Fleet Ion Trap equipped with a standard Ionspray interface. HRMS were performed on a LTQ-IT-Orbitrap with a spray voltage of 2.10 kV and a resolution of 100000 (FWHM). ^{1}H NMR spectra were obtained at 500 MHz. Chemical shifts are reported in part per million (δ), using the central solvent line as internal reference. ^{13}C NMR spectra were obtained at 125 MHz and 175 MHz. Chemical shifts are reported in δ, using the central solvent line as internal reference.

Materials. Reagents were purchased from commercial suppliers and used without purification. Compounds **4** and **8** were prepared according to known methods.[16,32]

Synthesis of receptor 3.

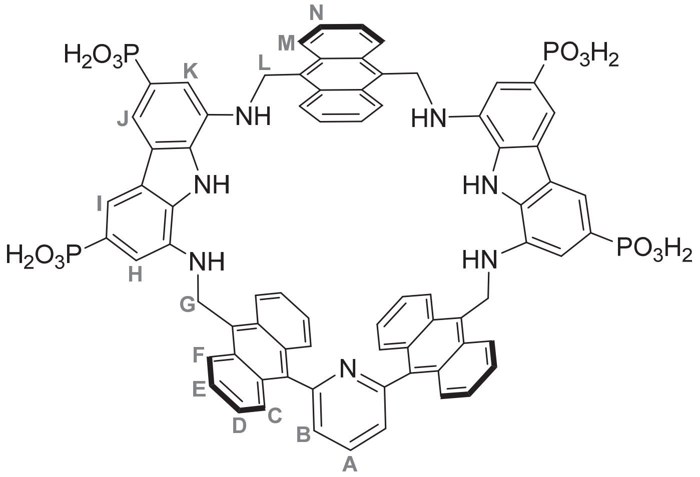

In anhydrous conditions and under N_2 atmosphere, **13** (99 mg, 0.062 mmol) was dissolved in dry DCM (700 µL) and Et_3N (140 µL, 0.988 mmol). The solution was cooled to 0°C and TMSBr was added (130 µL, 0.988 mmol). The solution was let return to RT and stirred for 3h. After 3h the solution was cooled to 0°C and MeOH was slowly added (1 mL). After the addition the solution was stirred at RT for 0.5h and the solvent was evaporated to afford 236 mg of crude as a yellow solid. Crude was suspended in HCl 0.05 M (10 mL) e centrifuged (10' x 4000 rpm x 8). The supernatant was removed, and the pellet was suspended in MeOH (2 mL) and centrifuged (10' x 4000 rpm x 2). The supernatant was removed, and the pellet dried under high vacuum to get 73 mg of pure **3** as a yellow solid (86%). ^{1}H NMR (500 MHz, D_2O + NaOD, δ = 4.79): δ 8.54 (d, J = 8.9 Hz, 4H, H-F); 8.39 (t, J = 7.7 Hz, 1H, H-A); 8.31-8.29 (m, 4H, H-M); 8.17 (dd, J_1 = 12.2 Hz, J_2 =

0.8 Hz, 4H, H-I, H-J); 7.88-7.82 (m, 8H, H-B, H-H, H-C); 7.64-7.56 (m, 10H, H-K, H-E, H-D); 7.42-7.39 (m, 4H, H-N); 5.58 (s, 4H, CH_2-G); 5.31 (s, 4H, CH_2-L). ^{13}C NMR (125 MHz, D_2O + NaOD): ∂ 157.56 (C-A); 135.09; 133.53 (d, J = 20.2 Hz); 133.38; 133.25 (d, J = 16.8 Hz); 132.50 (d, J = 39.5 Hz); 130.94; 130.49; 130.19; 130.05; 129.83; 129.74; 129.53; 126.57; 126.48; 126.36 (C-C); 126.22 (C-D, C-N); 124.67 (C-M); 124.50 (C-F); 123.65 (d, J = 15.5 Hz); 122.98 (d, J = 15.8 Hz); 113.96 (d, J = 150.9 Hz, C-I, C-J); 112.90 (d, J = 9.8 Hz, C-H); 110.59 (d, J = 11.9 Hz, C-K); 42.59 (C-L); 41.16 (C-G). ^{31}P NMR (202 MHz, D_2O): ∂ 13.28; 12.96. HRMS (*m/z*): $[M-H]^-$, calcd. for $C_{75}H_{56}N_7O_{12}P_4^-$, 1370.29429; found, 1370.29387. Mp: > 218°C (dec.).

Synthesis of 5.

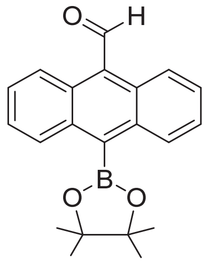

Under N_2 flux, **4** (752 mg, 2.64 mmol), bis(pinacolato)diboron (802 g, 3.17 mmol), KOAc (829 mg, 3.17 mmol) and $PdCl_2(PPh_3)_2$ (56 mg, 0.080 mmol) were suspended in 1,4-dioxane (12 mL). After three vacuum/N_2 cycles, the suspension was heated at 90°C and stirred for 18h. After 18h the mixture was cooled to RT and poured into THF (10 mL). The solution was evaporated to get 5.10 g of a green solid. The solid was dissolved in DCM (100 mL) and the organic layer washed with water (2 x 70 mL). The organic layer was then collected, dried over anhydrous Na_2SO_4, filtered and evaporated to get 1.28 g of crude. Crude was purified by flash-chromatography on silica gel with 30% petroleum ether in DCM as eluent (R_f = 0.60) to get 785 mg of pure **5** as a yellow solid (90%). ^1H NMR (500 MHz, $CDCl_3$, δ = 7.26): δ 11.55 (s, 1H,); 8.95-8.89 (m, 2H); 8.39-8.33 (m, 2H); 7.70-7.63 (m, 2H); 7.60-7.55 (m, 2H): 1.62 (s, 12H); ^{13}C NMR (125 MHz, $CDCl_3$, ∂ = 77.16): ∂ 194.02; 134.70; 130.70; 129.17; 128.38; 126.79; 125.82; 123.81; 85.16; 25.30. ESI-MS (*m/z*) %:333.25 (100%) $[M + H]^+$, 347.33 (35%) $[M + Na]^+$. mp: > 215°C (dec.).

Synthesis of 7.

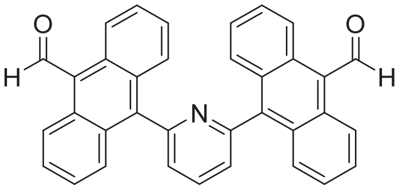

Under N_2 flux, **5** (1.13 g, 3.40 mmol), 2,6-dibrompyridine **6** (366 mg, 1.55 mmol), $CsCO_3$ (1.11 g, 3.40 mmol) and $PdCl_2(PPh_3)_2$ (120 mg, 0.171 mmol) were suspended in 1,4-dioxane (20 mL). After three vacuum/N_2 cycles, the suspension was heated at 100°C and stirred for 72h. After 72h the mixture was cooled to RT and evaporated to get 4.15 g of a dark solid. The solid was suspended in water (20 mL) and filtered. The solid was washed again with water (2 x 10 mL). and dried at high vacuum to get 850 mg of crude. Crude was suspended in $CHCl_3$ (20 mL) and the suspension was filtered. The filtrate was evaporated and the obtained solid was suspended in AcOEt (35 mL) and filtered. The solid was washed again with AcOEt (2 x 35 mL) and then dried at high vacuum to get 470 mg of pure **7** as a light-yellow solid (60%). ^1H NMR (500 MHz, $CDCl_3$, δ = 7.26): δ 11.58 (s, 2H); 8.99 (d, J = 9.0 Hz, 4H); 8.28 (t, J = 7.8 Hz, 1H), 7.85 (d, J = 8.8, 4H); 7.78 (d, J = 7.43 Hz, 2H); 7.72-7.66 (m, 4H); 7.58-7.53 (m, 4H). ^{13}C NMR (125 MHz, $CDCl_3$, ∂ = 77.16): ∂ 193.47; 158.14; 142.12; 137.05; 131.62; 129.77; 128.56; 126.93; 126.27; 126.22; 125.69; 123.82. ESI-MS (*m/z*) %:488.25 (100%). Mp: > 234°C (dec.).

Synthesis of 9.

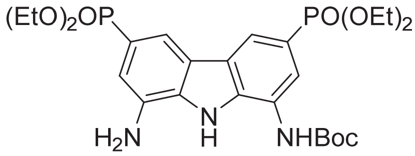

In anhydrous conditions and N_2 atmosphere, to a solution of **8** (2.00 g, 4.26 mmol) and Boc_2O (2.00 g, 9.16 mmol) in dry DCM (75 mL), DMAP was added (50 mg, 0.413 mmol). The solution was stirred for 72h at RT. After 72h the solution was evaporated to get 2.90 g of crude as a brown foamy solid. Crude was purified by flash-chromatography on silica gel with 10% MeOH in $CHCl_3$ as eluent (R_f = 0.55) to obtain 1.40 of pure **9** as an amorphous brown solid (60%). ^1H NMR (500 MHz, $CDCl_3$, δ = 7.26): δ 11.67 (s, 1H); 8.51 (s, 1H); 8.43 (d, J = 12.0 Hz, 1H); 8.28 (d, J = 13.6 Hz, 1H); 7.97 (d, J = 13.9 Hz, 1H); 7.15 (d, J = 13.3 Hz, 1H); 4.38 (s, 2H); 4.20-4.04 (m, 8H); 1.35 (s, 9H);

1-32-1.27 (m, J = 12H). ^{13}C NMR (125 MHz, $CDCl_3$, ∂ = 77.16): ∂ 153.38; 133.71; 133.00 (d, J = 18.9 Hz); 132.25 (d, J = 2.66 Hz); 124.91 (d, J = 20.4 Hz); 124.31 (d, J = 18.2 Hz); 123.55 (d, J = 19.2 Hz); 120.14 (d, J = 10.9 Hz); 118.58; 118.18; 116.88 (d, J = 53.6 Hz): 115.39 (d, J = 10.1 Hz); 112.87 (d, J = 13.5 Hz); 80.60; 62.44 (t, J = 5.5 Hz); 80.21; 16.32 (d, J = 3.2 Hz); 16.28 (d, J = 3.1 Hz). ESI-MS (*m/z*) %: 592.42 (100%) $[M + Na]^+$; 608.42 (45%) $[M + K]^+$; 1161.42 (45%) $[2M + Na]^+$.

Synthesis of 10.

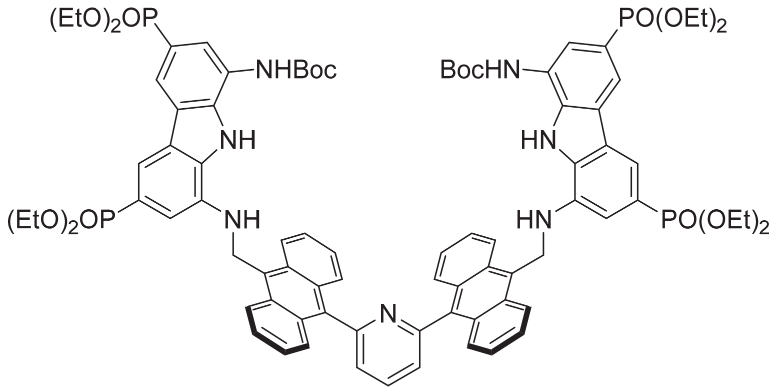

To a suspension of **9** (818 mg, 1.44 mmol) and **7** (100 mg, 0.205 mmol) in $CHCl_3$ (7 mL), TFA was added (5 µL, 0.065 mmol). After 4h at RT, the reaction was quenched with Et_3N (50 µL) and diluted with $CHCl_3$ (20 mL). The organic layer was washed with $NaHCO_3$ (1 x 20 mL) and water (1 x 20 mL). Organic layer was collected, dried over anhydrous Na_2SO_4, filtered and evaporated to get 1.20 g of crude as a red solid. To the solid, a freshly-prepared suspension of $NaBH_4$ (117 mg, 3.09 mmol) in MeOH (10 mL) was added. The mixture was stirred for 0.5h, then diluted with DCM (20 mL) and washed with water (2 x 15 mL). Organic layer was collected, dried over anhydrous Na_2SO_4, filtered and evaporated to get 1.09 g of crude. Crude was purified by flash-chromatography on silica gel with 5% MeOH in $CHCl_3$ as eluent (R_f = 0.30) to get 99 mg of pure **14** as a yellow solid (35%). ^{1}H NMR (500 MHz, MeOD, δ = 3.31): δ 8.43 (d, J = 9.0 Hz, 4H); 8.37 (t, J = 7.7 Hz, 1H); 8.30 (d, J = 14.5 Hz, 2H); 8.04 (d, J = 14.5 Hz, 2H); 7.93 (bs, 2H); 7.81 (d, J = 8.7 Hz, 4H); 7.77 (d, J = 7.7 Hz, 2H); 7.53-7.45 (m, 10H); 5.42 (s, 4H); 4.24-4.16 (m, 8H); 4.14-4.06 (m, 8H); 1.39 (t, J = 6.9 Hz, 12H); 1.31 (t, J = 6.9 Hz, 12H); 1.26 (s, 18H). ^{13}C NMR (125 MHz, MeOD, ∂ = 49.00): ∂ 159.84; 155.32; 139.36; 136.77; 136.50 (d, J = 17.7 Hz); 132.83 (d, J = 2.8 Hz); 132.02; 131.52; 131.34; 127.73; 127.45; 127.01; 125.70; 124.92 (d, J = 20.1 Hz); 123.85 (d, J = 21.4 Hz); 122.06 (bs); 120.77; 119.40 (d, J = 37.8 Hz); 117.99; 115.65 (d, J = 10.1 Hz); 108.84 (d, J = 14.9 Hz); 81.51; 79.45; 63.69 (t, J = 4.9 Hz); 28.51; 16.81 (d, J = 6.3 Hz); 16.65 (d, J = 6.2 Hz).

Synthesis of 11.

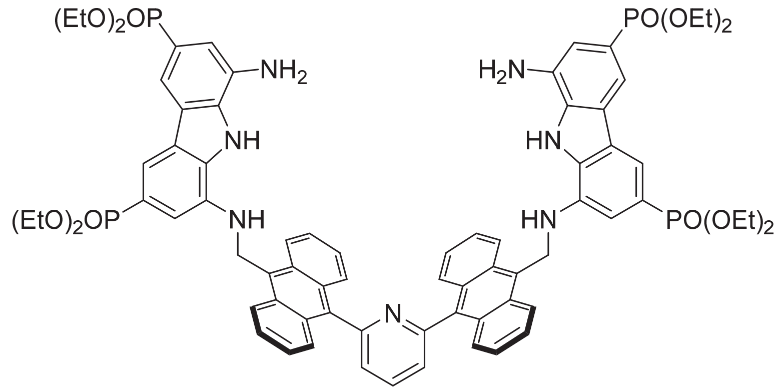

To a solution of **10** (130 mg, 0.082 mmol) in DCM (2 mL), cooled to 0°C, TFA (437 µL, 5.70 mmol) was slowly added. The solution was stirred at 0°C for 2h, then the solution was let return to RT and diluted with DCM (10 mL). The organic layer was washed with $NaHCO_{3ss}$ (2 x 10 mL) and water (1 x 10 mL). Organic layer was collected, dried over anhydrous Na_2SO_4, filtered and evaporated to get 105 mg of crude. Crude was solubilized in 2 mL of $CHCl_3$ and AcOEt was added. The obtained suspension was centrifuged (10’ x 4000 rpm). The pellet was collected and dried under high vacuum to get 46 mg of pure **11** (40%). ^{1}H NMR (500 MHz, MeOD, δ = 3.31): δ 8.46-8.41 (m, 5H); 8.00 (d, J = 15.3 Hz, 2H); 7.93 (dd, J_1 = 14.4 Hz, J_2 = 1.1 Hz, 2H); 7.85-7.82 (m, 6H); 7.57-7.50 (m, 8H); 7.45 (d, J = 13.3Hz, 2H); 7.06 (dd, J_1 = 13.5 Hz, J_2 = 1.1 Hz, 2H); 5.47 (s, 4H); 4.22-4.15 (m, 8H); 4.12-4.06 (m, 8H); 1.41 (t, J = 6.9 Hz, 12H); 1.31 (t, J = 6.9 Hz, 12H).

Synthesis of 13.

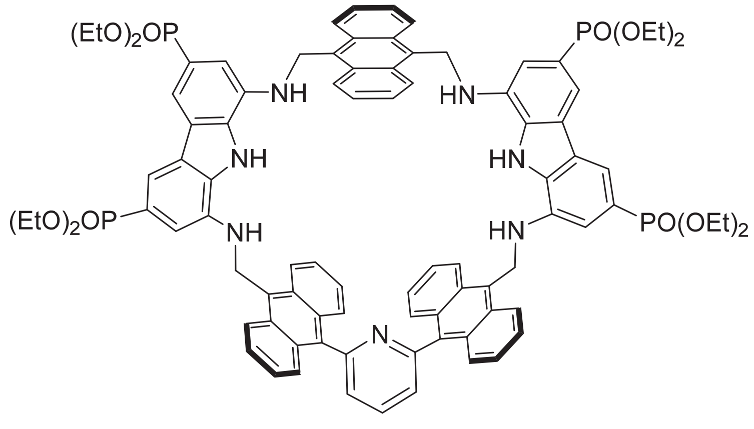

To a suspension of **7** (200 mg, 0.410 mmol) and **15** (570 mg, 0.410 mmol) in a 1:1 mixture of MeOH:$CHCl_3$ (80 mL), a 0.8 M solution of TFA in $CHCl_3$ (213 µL, 0.164 mmol) was added. The

resulting solution was refluxed for 21h, and then it was let return to RT, neutralized with Et_3N and evaporated. The resulting solid was dissolved in $CHCl_3$ (150 mL) and the organic layer was washed with $NaHCO_{3ss}$ (2 x 100 mL) and then with water (1 x 100 mL). Organic layer was collected, dried over anhydrous Na_2SO_4, filtered and evaporated to get 810 mg of crude. Crude was purified by flash-chromatography on silica gel with 20% THF in DCM + 5% MeOH as eluent (R_f = 0.50) to get 481 mg of pure imine **16** as a red solid (74%). To a solution of **16** (460 mg, 0.289 mmol) in $CHCl_3$ (15 mL), cooled to 0°C, a freshly prepared suspension of $NaBH_4$ in MeOH (133 mg, 3.52 mmol, 40 mL of MeOH) was slowly added and the mixture was then vigorously stirred at RT for 0.5h. After 0.5h the mixture was poured into water (150 mL) and the aqueous layer was extracted with $CHCl_3$ (3 x 50 mL). The organic layers were collected and washed with water (1 x 100 mL). Organic layer was collected, dried over anhydrous Na_2SO_4, filtered and evaporated to get 460 mg of crude. Crude was purified by flash-chromatography on silica gel with 20% THF in $CHCl_3$ + 5% MeOH as eluent (R_f = 0.50) to get 445 mg of pure **13** as a yellow solid (> 90%). **16**: ^{1}H NMR (500 MHz, $CDCl_3$ + 10% MeOD, δ = 7.26): δ 10.05 (s, 2H); 9.83 (s, 2H); 8.59-8.52 (m, 6H); 8.29 (t, J = 7.5 Hz, 1H); 8.25 (d , J = 14.2 Hz); 8.19-8.12 (m, 4H); 8.00 (d, J = 13.4 Hz, 2H); 7.84 (d, J = 7.5 Hz, 2H); 7.74 (d, J = 8.7 Hz, 4H); 7.53-7.47 (m, 4H); 7.46-7.36 (m, 10H); 5.10-5.04 (m, 4H); 4.91-4.86 (m, 2H); 4.36-4.16 (m, 16H); 1.49-1.39 (m, 24H). ^{13}C NMR (125 MHz, $CDCl_3$ + 10% MeOD, ∂ = 77.16): ∂ 160.55; 158.19; 139.00 (d, J = 2.3 Hz); 137.79 (d, J = 2.9 Hz); 136.79; 136.66; 134.82 (d, J = 17.6 Hz); 131.67 (d, J = 3.4 Hz); 130.84; 130.11; 129.65; 129.53; 128.84; 126.84; 126.80; 126.12; 125.76; 125.02; 124.89; 124.73; 124.60; 123.51 (d, J = 8.5 Hz); 123.33; 120.60 (d, J = 83.1 Hz); 119.05 (d, J = 83.1 Hz); 116.10 (d, J = 10.5 Hz); 115.74 (d, J = 13.0 Hz); 110,04 (d, J = 14.2 Hz); 62.33 (d, J = 5.5 Hz); 62.14 (d, J = 5.7 Hz); 43.05; 16.55 (d, J = 2.4 Hz); 16.50 (d, J = 2.3 Hz). ESI-MS (*m/z*) %: 421.33 (100%) [M + 4Na]$^{4+}$; 818.75 (70%) [M + 2Na]$^{2+}$; 1615.25 (100%) [M + Na]$^{+}$, 1591.25 (100%) [M + H]$^{+}$. Mp: > 250°C (dec.). **13**: ^{1}H NMR (500 MHz, $CDCl_3$ + 5% MeOH, δ = 7.26): δ 10.76 (s, 2H); 8.34 (d, J = 8.9 Hz, 4H); 8.25 (t, J = 7.6 Hz, 1H); 8.14-8.07 (m, 8H); 7.71 (d, J = 7.7 Hz, 2H); 7.63 (d, J = 9.0 Hz, 4H); 7.52-7.37 (m, 12H); 7.30-7.24 (m, 4H); 5.39 (d, J = 3.9 Hz, 4H); 5.02 (s, 4H); 4.97-4.92 (m, 2H); 4.92-4.86 (m, 2H); 4.32-4.07 (m, 16H), 1.43 (t, J = 7.3 Hz, 12H); 1.34 (t, J = 7.3 Hz, 12H). ^{13}C NMR (125 MHz, $CDCl_3$ + 5% MeOH, ∂ = 77.16): ∂ 158.51; 138.23; 135.0; 134.88 (d, J = 3.0 Hz); 134.85; 131.41 (dd, J_1 = 24.6 Hz, J_2 = 3.0 Hz); 130.95; 130.67; 130.25; 129.91; 129.76; 126.38; 126.28; 126.23; 126.03; 126.00; 124.82; 124.65; 123.85 (d, J = 2.0 Hz); 123.70 (d, J = 2.0 Hz); 123.58 (d, J = 2.0 Hz); 123.43 (d, J = 2.7 Hz); 119.41 (d, J = 4.9 Hz); 117.88 (d, J = 5.1 Hz); 115.96 (d, J = 11.3 Hz); 115.45 (d, J = 11.0 Hz); 108.72 (d, J = 14.7 Hz); 107.62 (d, J = 14.4 Hz); 62.41 (d, J = 5.5 Hz); 62.29 (d, J = 4.5 Hz); 43.2;

41.2; 16.50 (d, J = 6.7 Hz); 16.42 (d, J = 6.7 Hz). ESI-MS (m/z) %: 1595.33 (100%) $[M - H]^-$. Mp: > 248°C (dec.).

Synthesis of 15.

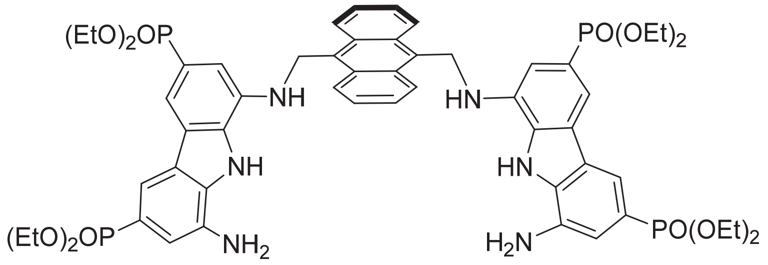

To a solution of **9** (1.30 g, 2.28 mmol) and 9,10-anthracenedicarboxaldehyde **12** (265 mg, 1.13 mmol) in $CHCl_3$ (21 mL), AcOH (750 µL, 13.00 mmol) was added. The solution was heated to reflux for 17h. After 17h the mixture was let return to RT, neutralized with Et_3N and diluted with 150 mL of $CHCl_3$. The organic layer was washed with $NaHCO_{3ss}$ (1 x 100 mL) and then with water (1 x 100 mL). Organic layer was collected, dried over anhydrous Na_2SO_4, filtered and evaporated to get 1.59 g of crude as a red solid. To the solid, a freshly prepared suspension of $NaBH_4$ (513 mg, 13.6 mmol) in MeOH (20 mL) was added. The mixture was vigorously stirred for 1h. Then, the mixture was evaporated and the obtained solid suspended in water (25 mL) and filtered. The resulting yellow solid was washed again with water (2 x 20 mL) and dried under high vacuum to get 2.00 g of crude. Crude was dissolved in DCM (10 mL) and the solution was cooled to 0°C, then, TFA (6.00 mL, 78 mmol) was slowly added and the solution was stirred at 0°C for 2h. The solution was let return to RT and diluted with DCM (150 mL). The organic layer was washed with $NaHCO_{3ss}$ (2 x 100 mL) and then with water (2 x 100 mL). Organic layer was collected, dried over anhydrous Na_2SO_4, filtered and evaporated to get 1.22 g of crude. Crude was suspended in MeOH (10 mL) and the suspension centrifuged (10' x 4000 rpm x 3). The pellet was dried under high vacuum to get 960 mg of pure **15** as a light-brown solid (74%). ^{1}H NMR (500 MHz, DMSOd$_6$, δ = 2.50): δ 11.05 (s, 2H); 8.53-8.47 (m, 4H) ; 7.95 (d, J = 14.2 Hz, 2H); 7.84 (d, J = 14.0 Hz, 2H); 7.67-7.63 (m, 4H); 7.36 (d, J = 13.5 Hz, 2H); 7.00 (d, J =13.4 Hz, 2H); 5.83 (m, 2H); 5.43 (d, J = 3.5 Hz, 4H); 5.12 (s, 4H); 4.16-4.10 (m, 8H); 4.04-4.95 (m, 8H); 1.35 (t, J = 7.1 Hz, 12H); 1.25 (t, J = 7.0 Hz, 12H). ^{13}C NMR (125 MHz, DMSOd$_6$, δ = 39.52): ∂ 134.93 (d, J = 18.2 Hz); 134.09 (d, J = 18.6 Hz); 131.05; 130.56; 130.53; 130.17; 126.39; 125.17; 122.69 (d, J = 19.8 Hz); 122.38 (d, J = 18.8 Hz); 119.85; 119.58; 118.35; 118.08; 114.17 (d, J = 11.7 Hz); 113.71 (d, J = 10.8 Hz); 111.51 (d, J = 12.1 Hz); 106.57 (d, J = 14.5 Hz); 61.39 (d, J = 5.2 Hz); 61.17 (d, J = 5.2 Hz); 30.67; 16.37

(d, *J* = 5.8 Hz); 16.24 (d, *J* = 5.8 Hz). ESI-MS (*m/z*) %: 593.58 (100%) $[M+2Na]^{2+}$; 1163.67 (88%) $[M+Na]^{+}$; 1179.67 (25%) $[M+K]^{+}$. Mp: > 225°C (dec.).

NMR spectra.

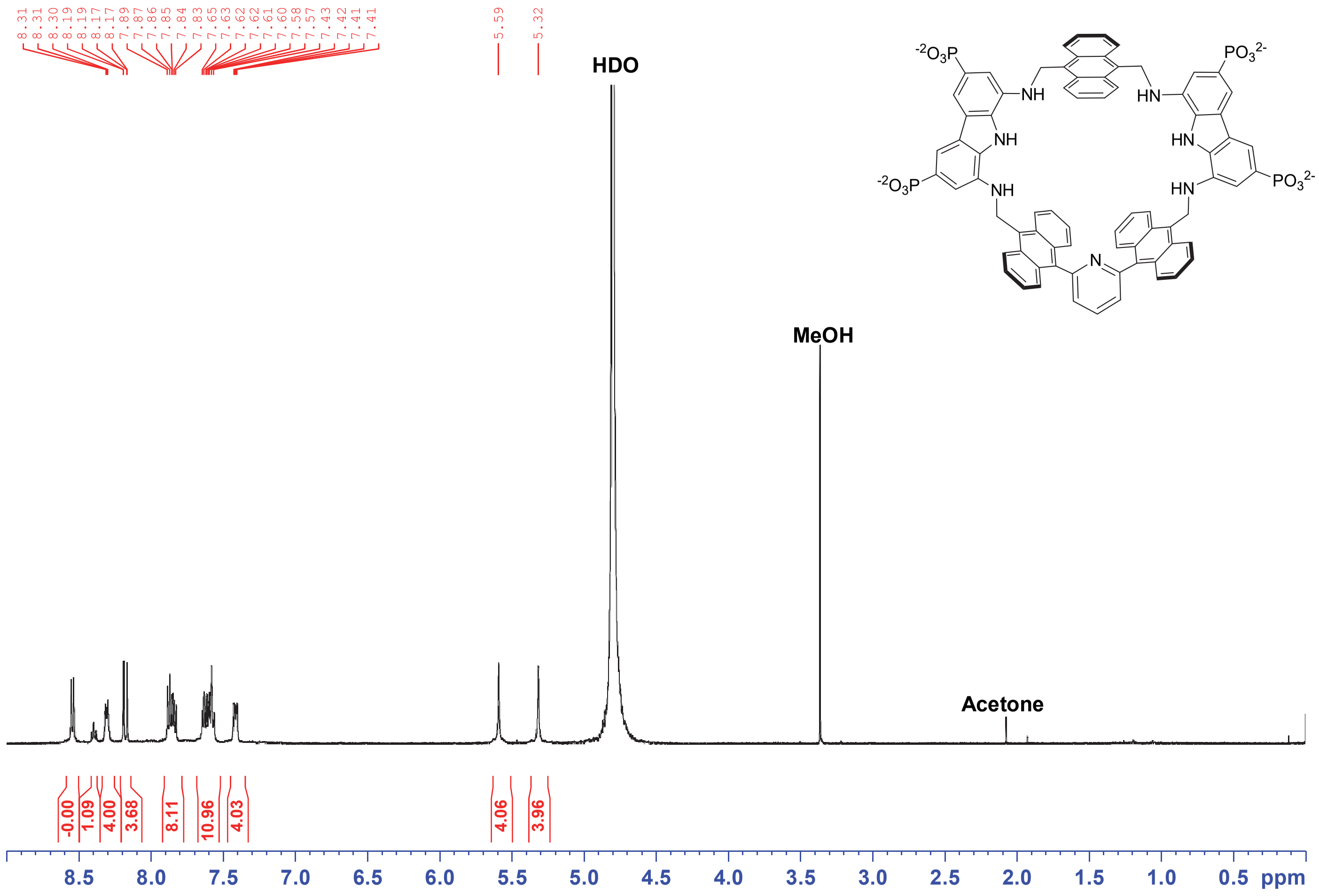

Figure S1. ^{1}H NMR spectrum of **3** (D_2O + NaOD - 500 MHz).

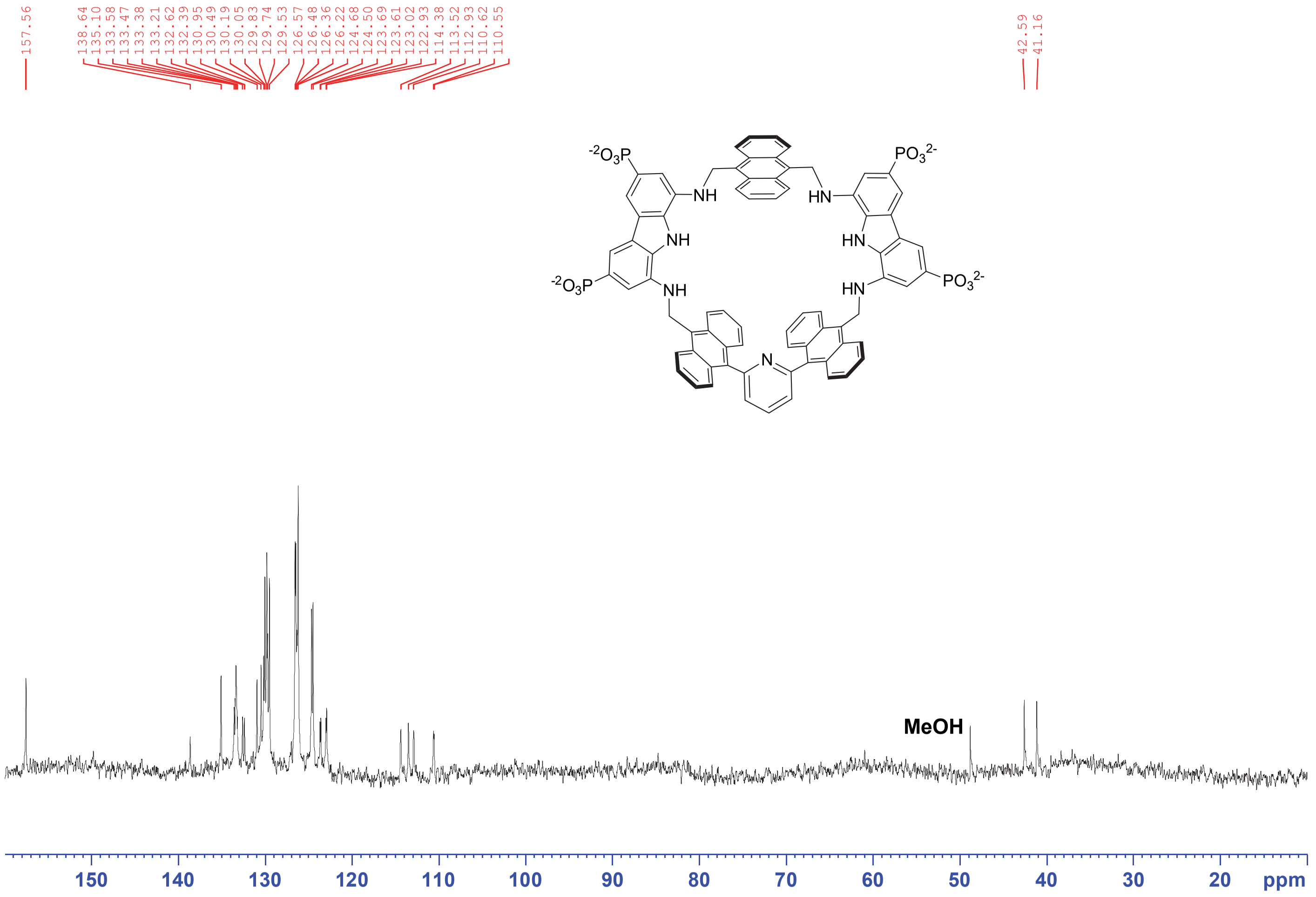

Figure S2. ^{13}C NMR spectrum of **3** (D_2O + NaOD – 175 MHz).

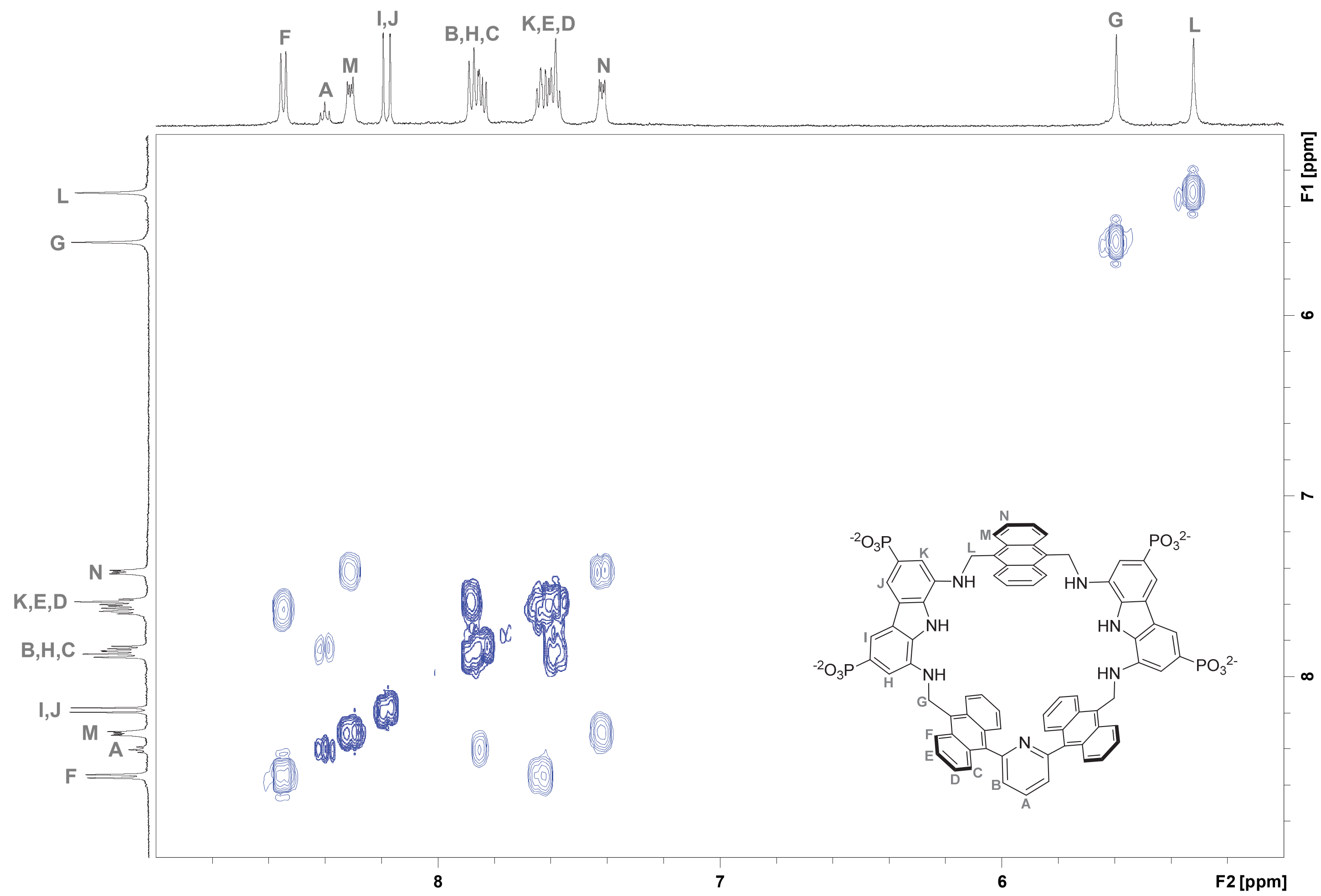

Figure S3. COSY spectrum of **3** (D_2O + NaOD - 500 MHz).

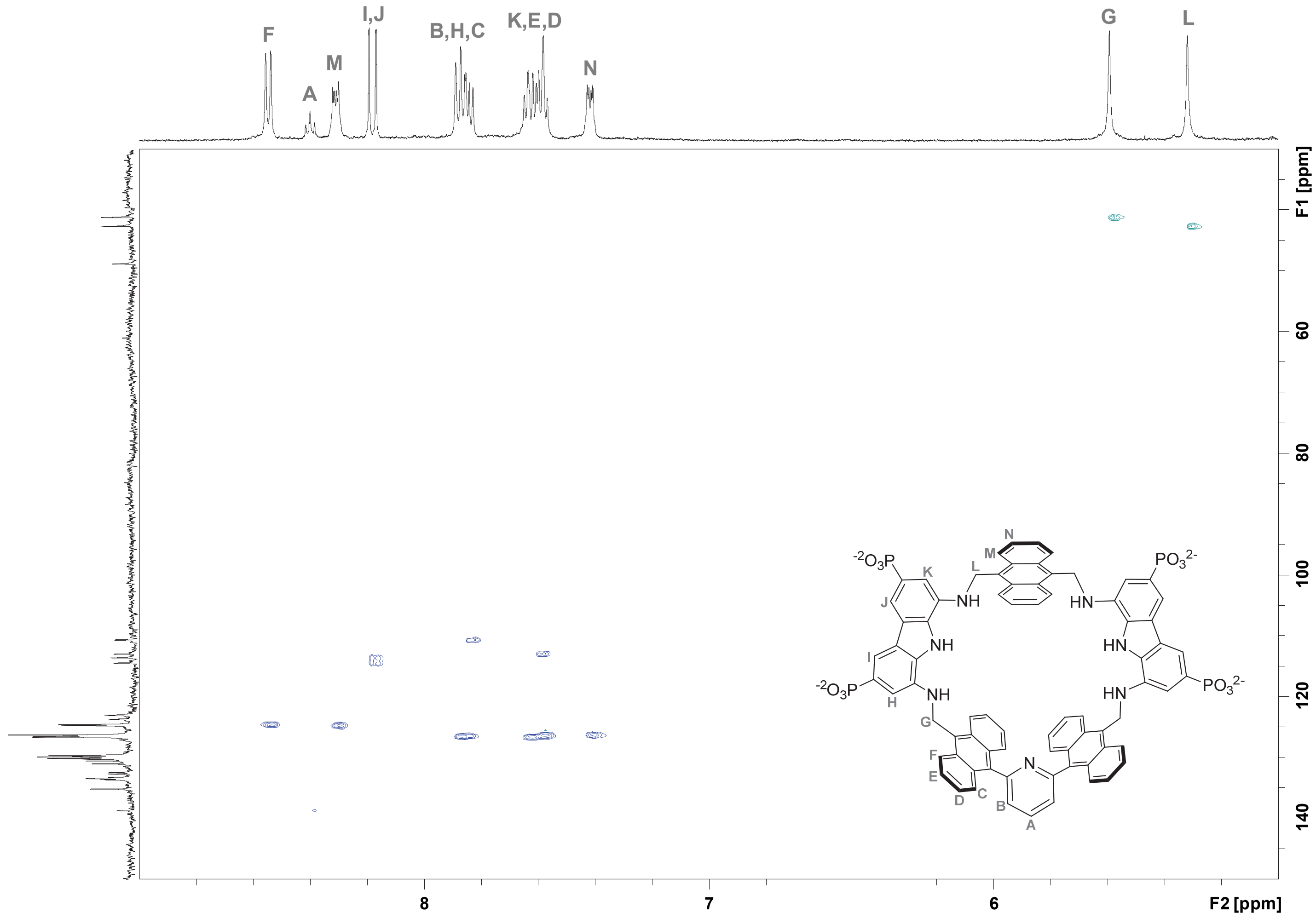

Figure S4. HSQC spectrum of **3** (D_2O + NaOD - 500 MHz).

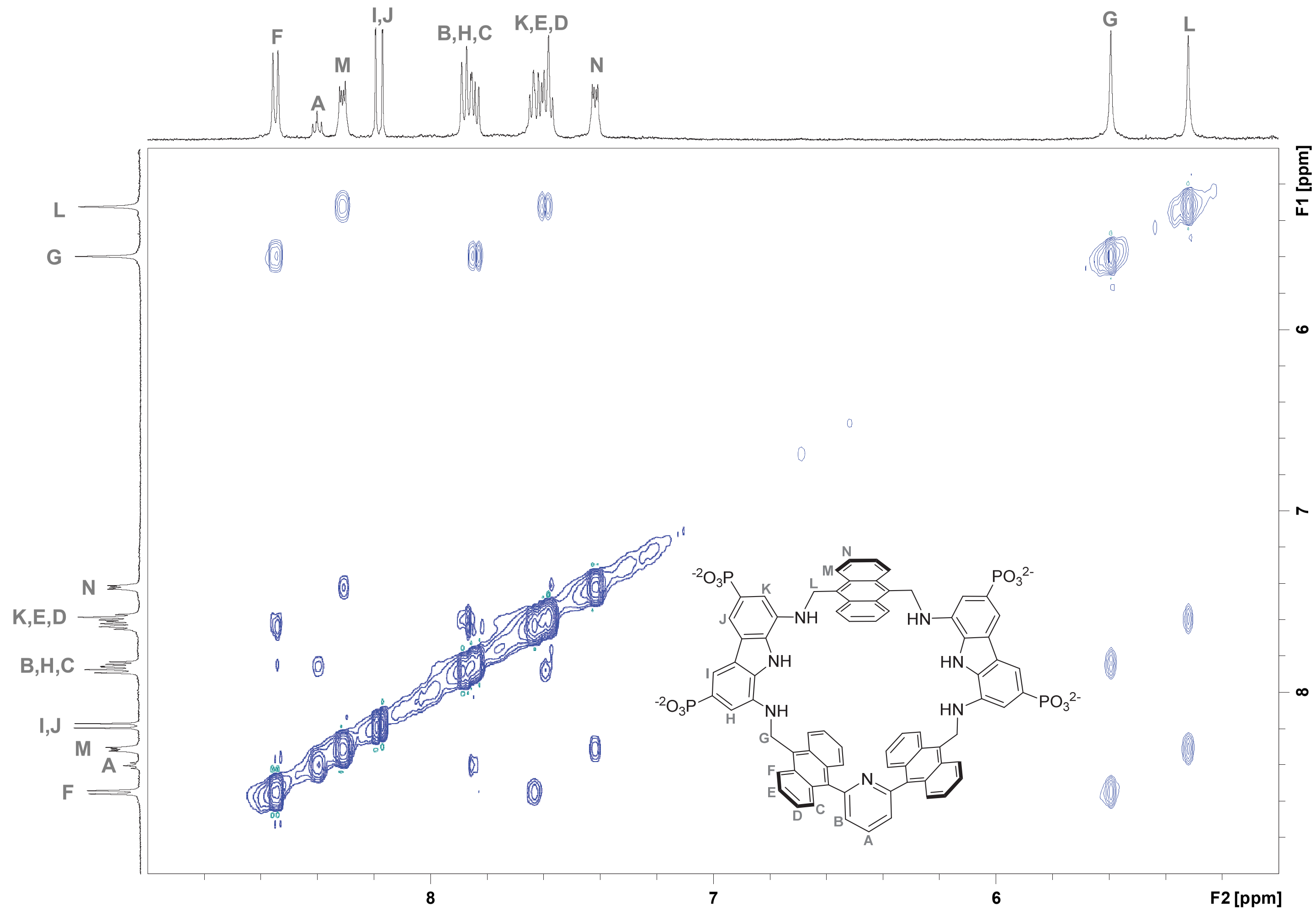

Figure S5. NOESY spectrum of **3** (D_2O + NaOD - 500 MHz).

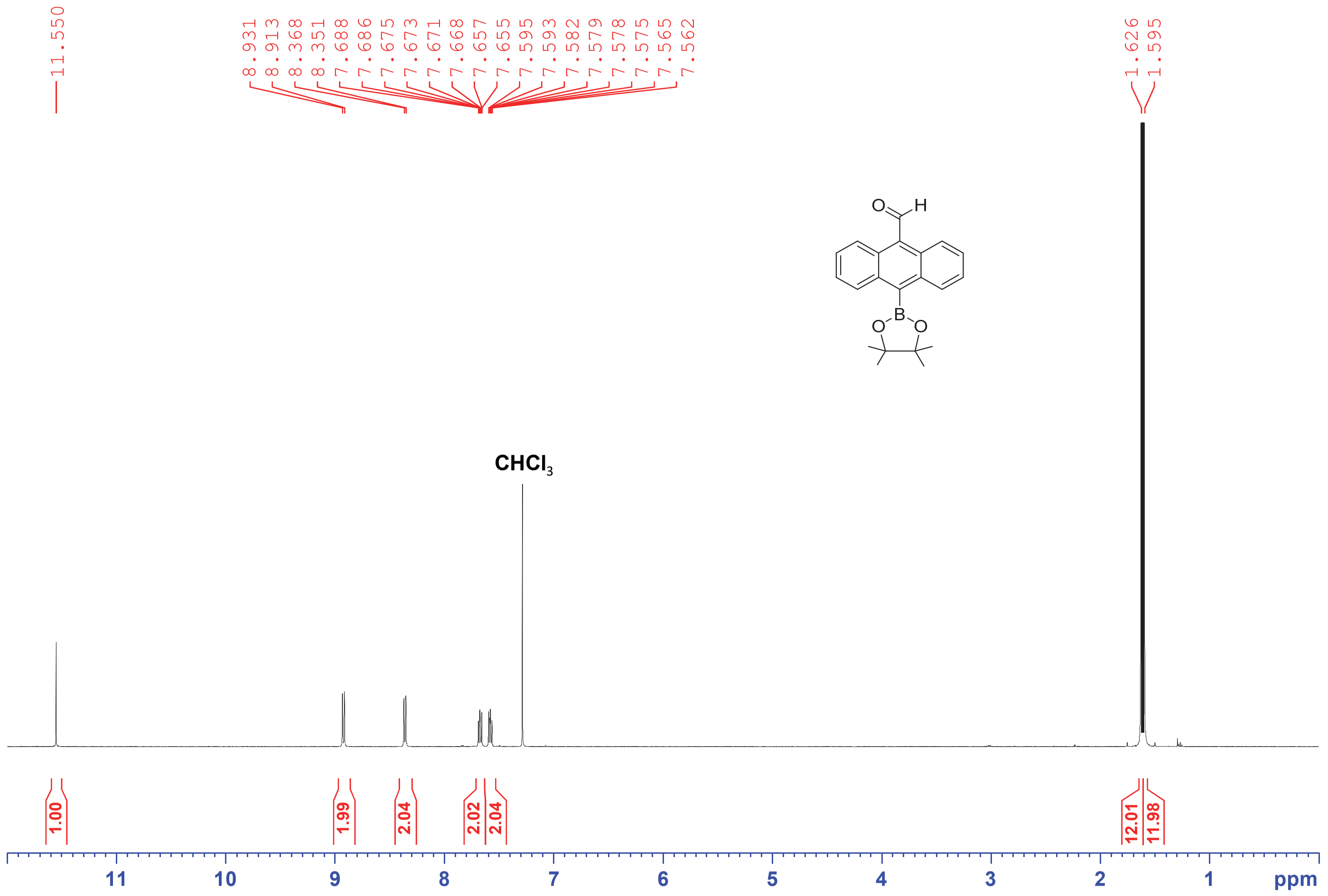

Figure S6. ^{1}H NMR spectrum of **5** ($CDCl_3$ - 500 MHz).

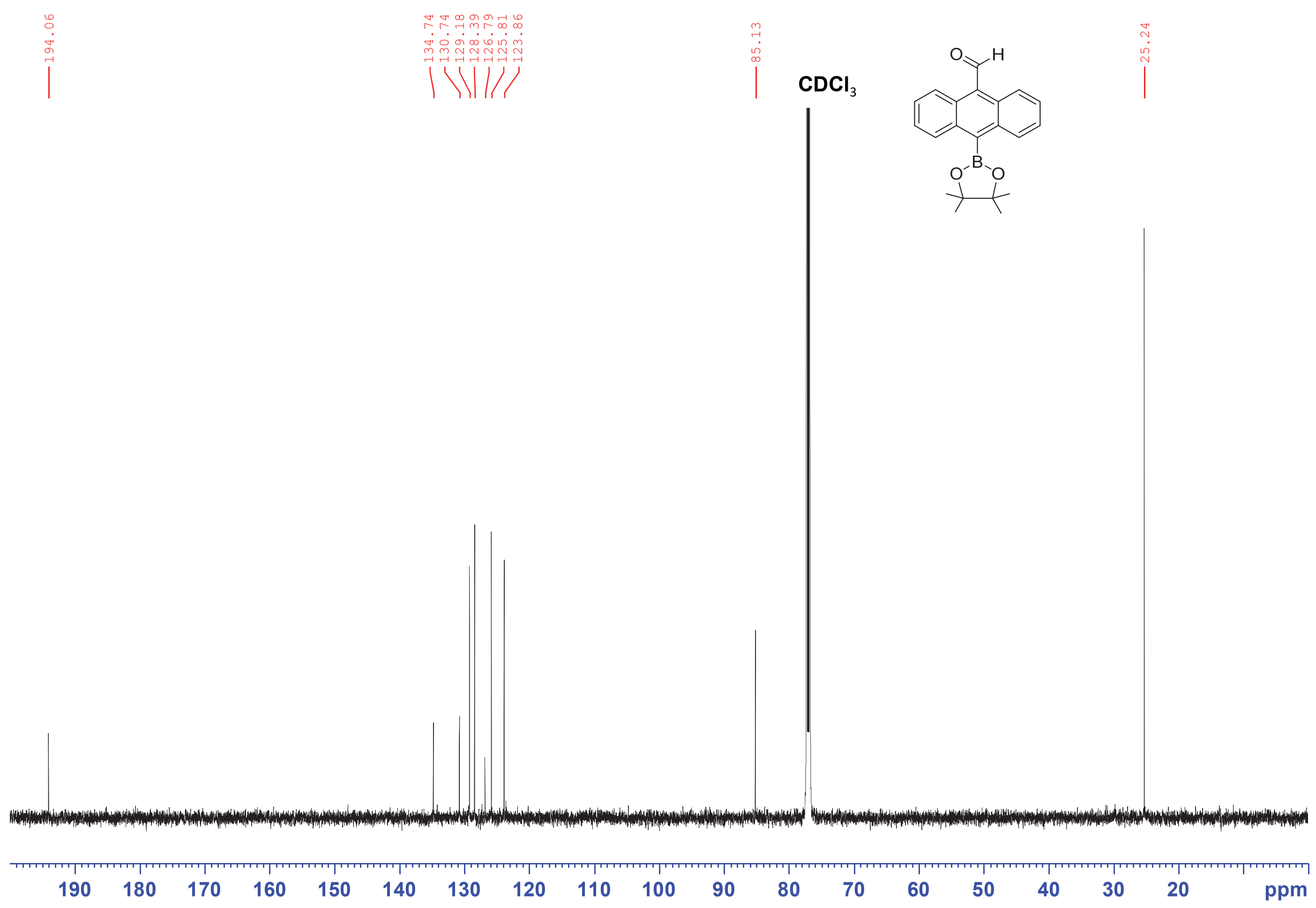

Figure S7. ^{13}C NMR spectrum of **5** ($CDCl_3$ – 125 MHz).

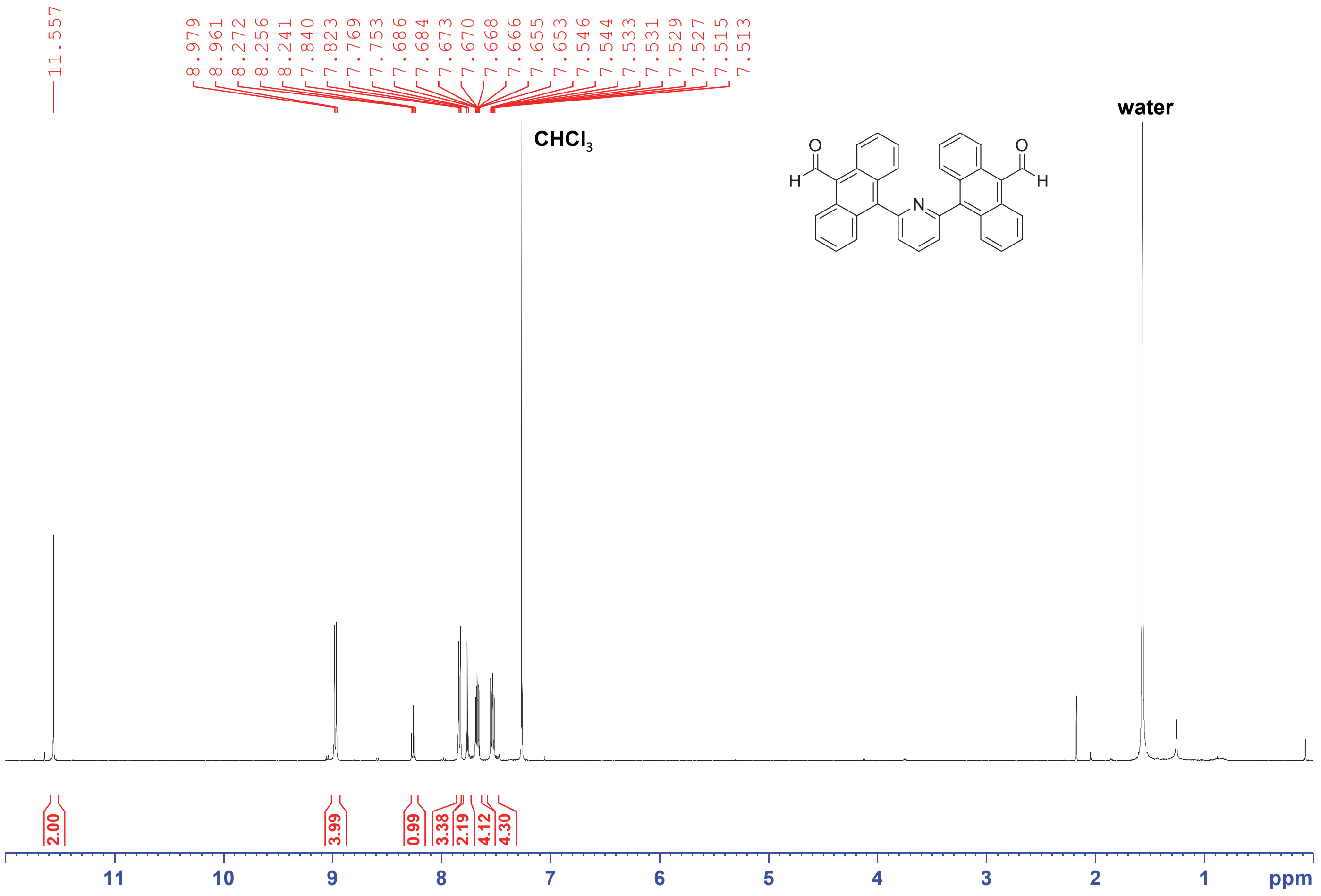

Figure S8. ^{1}H NMR spectrum of **7** ($CDCl_3$ - 500 MHz).

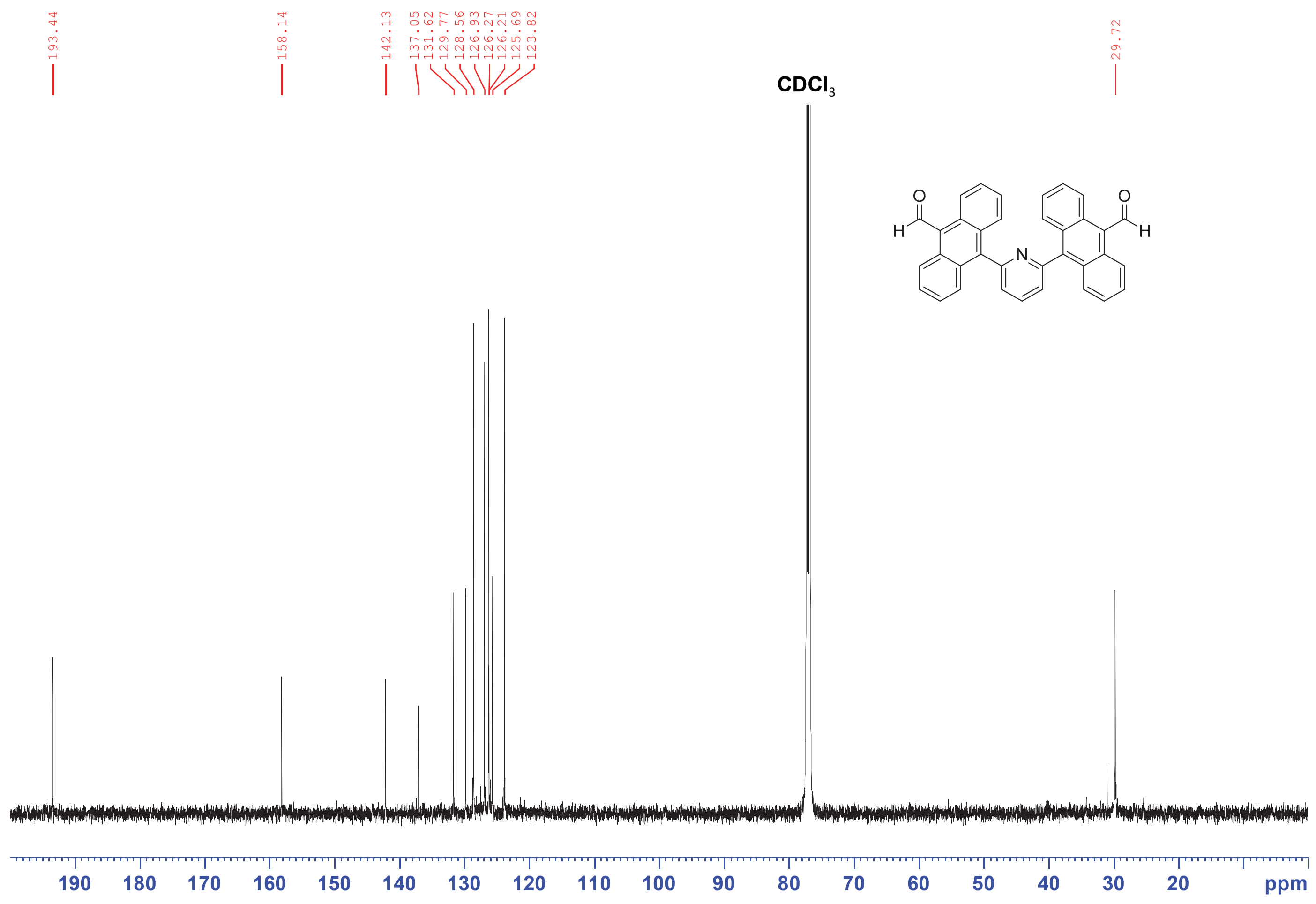

Figure S9. ^{13}C NMR spectrum of **7** ($CDCl_3$ – 125 MHz).

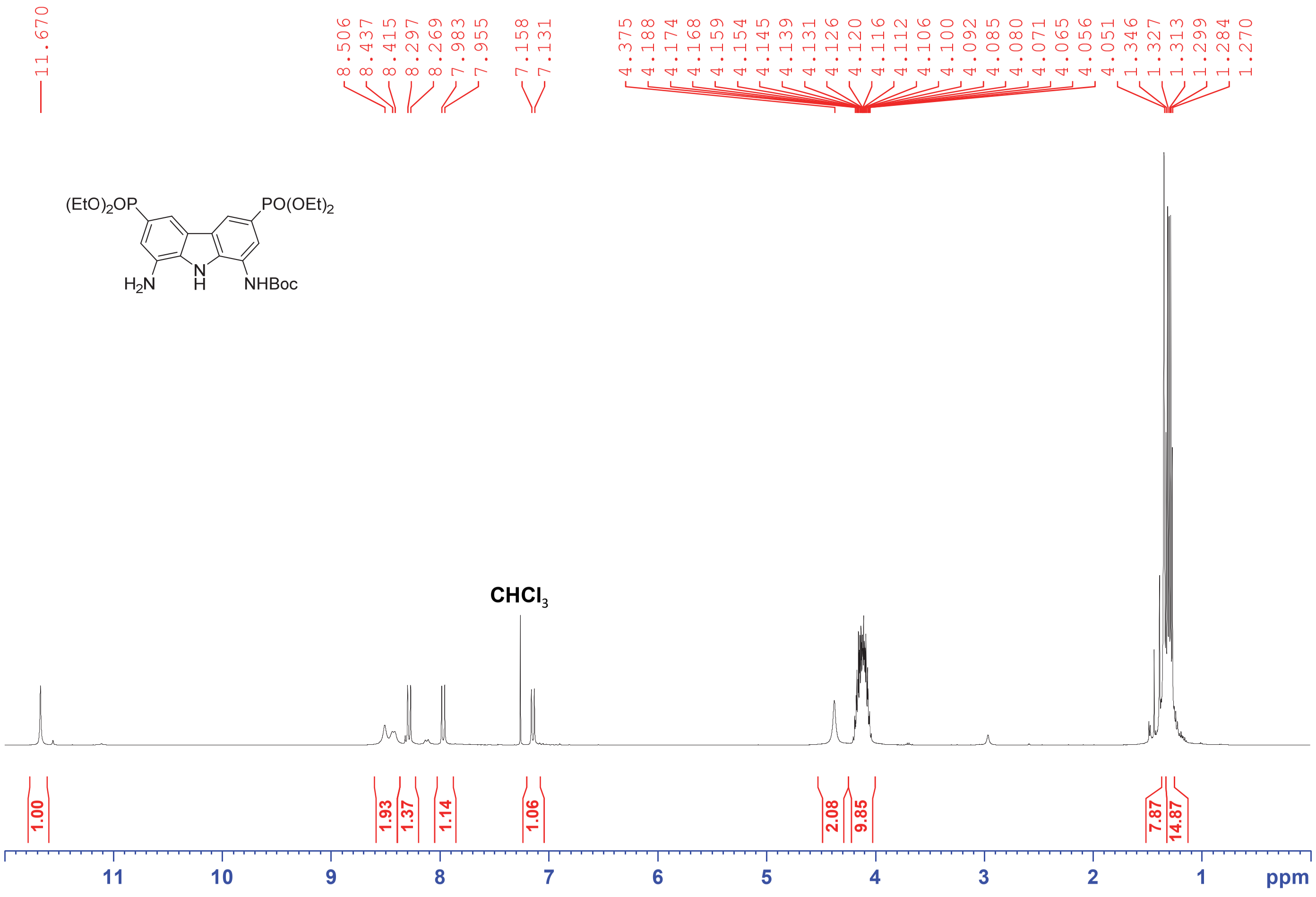

Figure S10. ^{1}H NMR spectrum of **9** ($CDCl_3$ - 500 MHz).

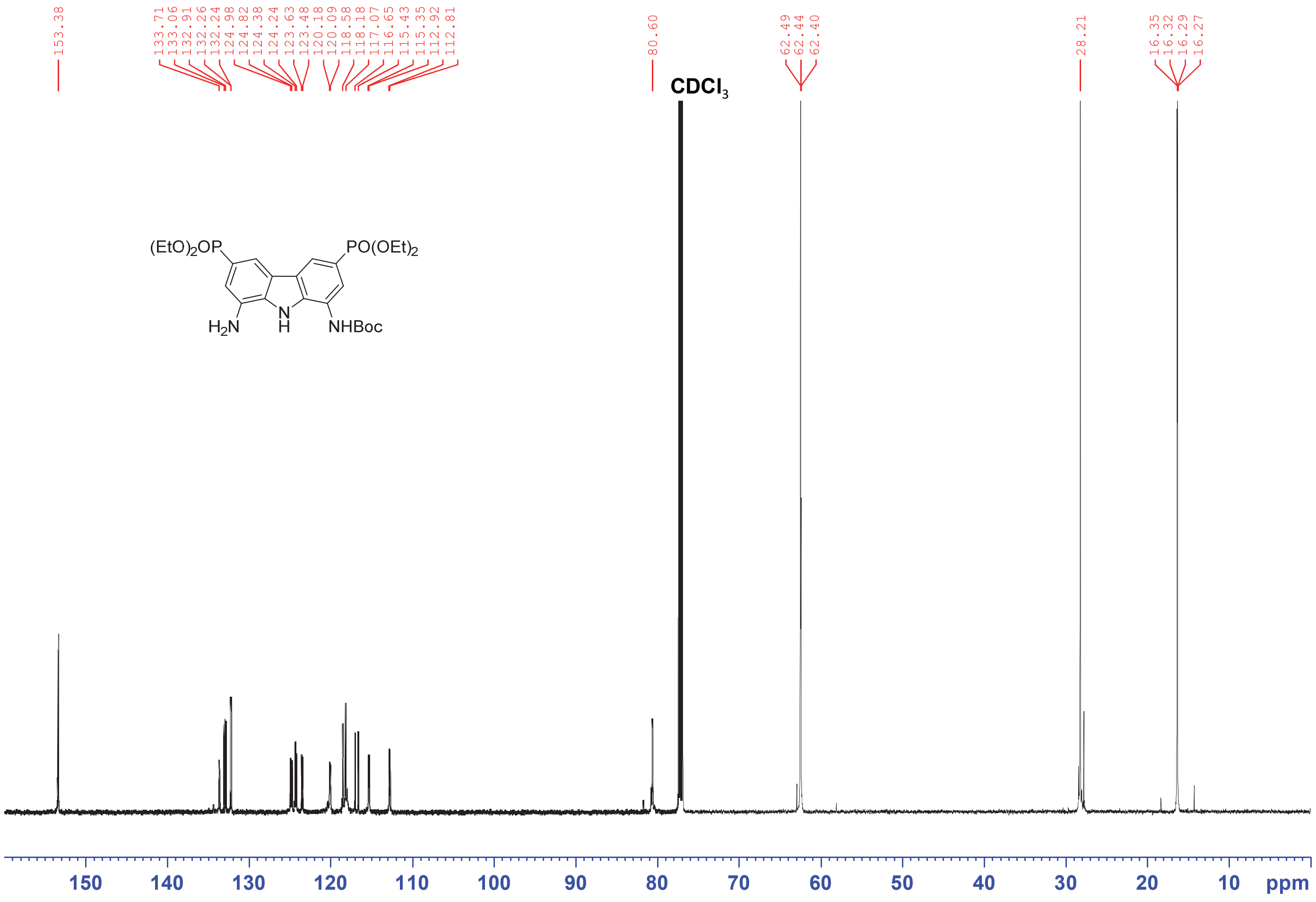

Figure S11. ^{13}C NMR spectrum of **9** ($CDCl_3$ – 125 MHz).

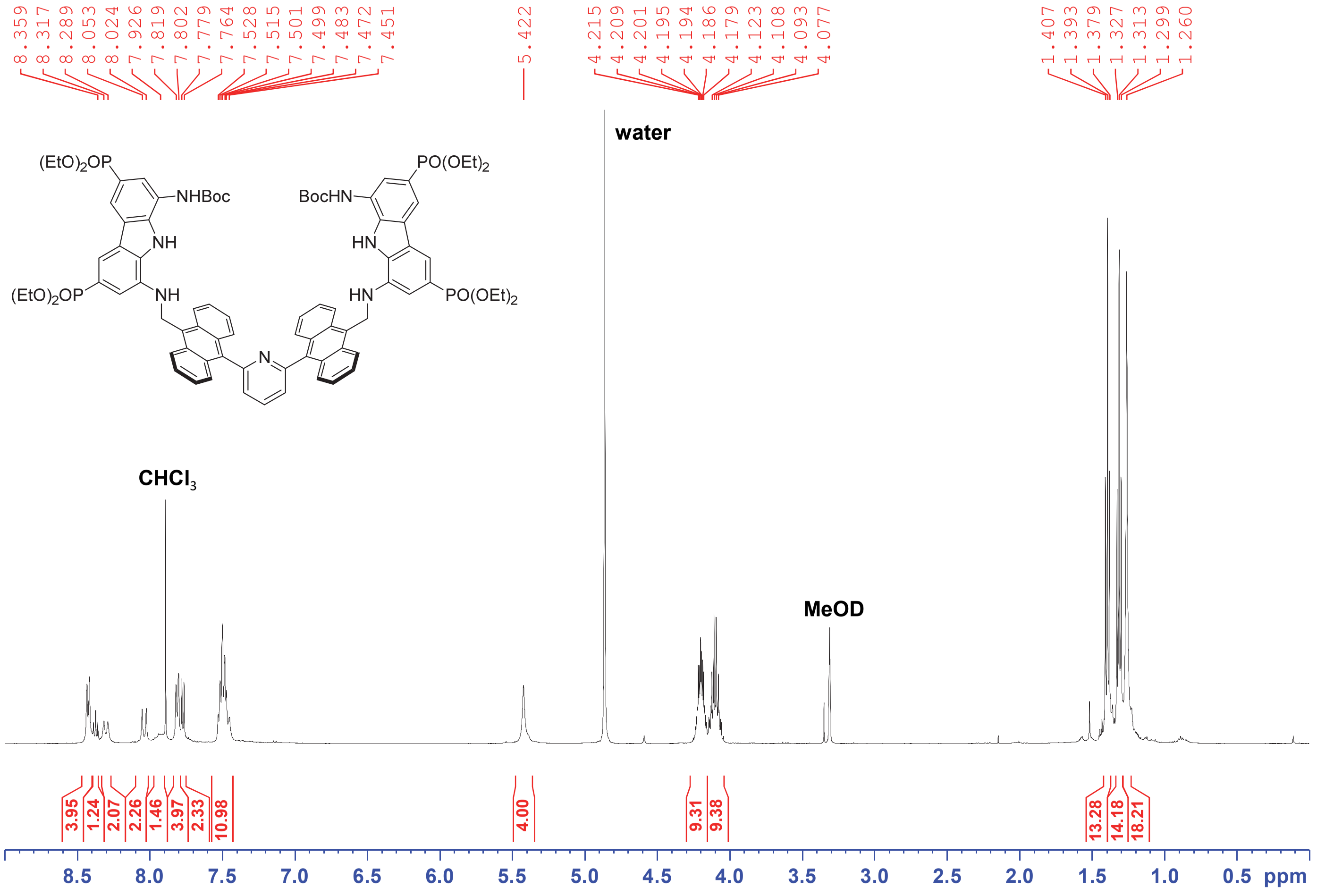

Figure S12. ^{1}H NMR spectrum of **10** (MeOD - 500 MHz).

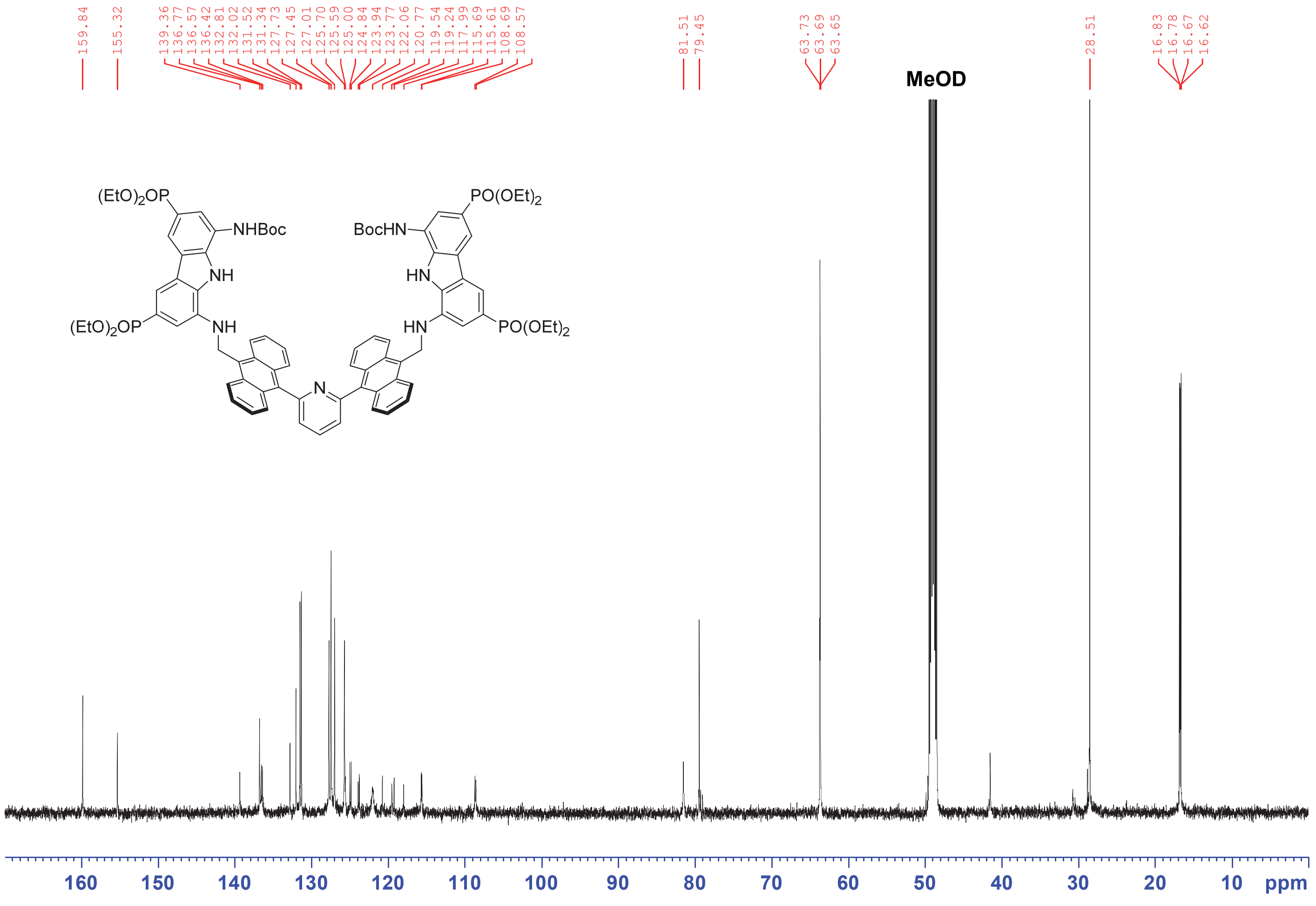

Figure S13. ^{13}C NMR spectrum of **10** (MeOD – 125 MHz).

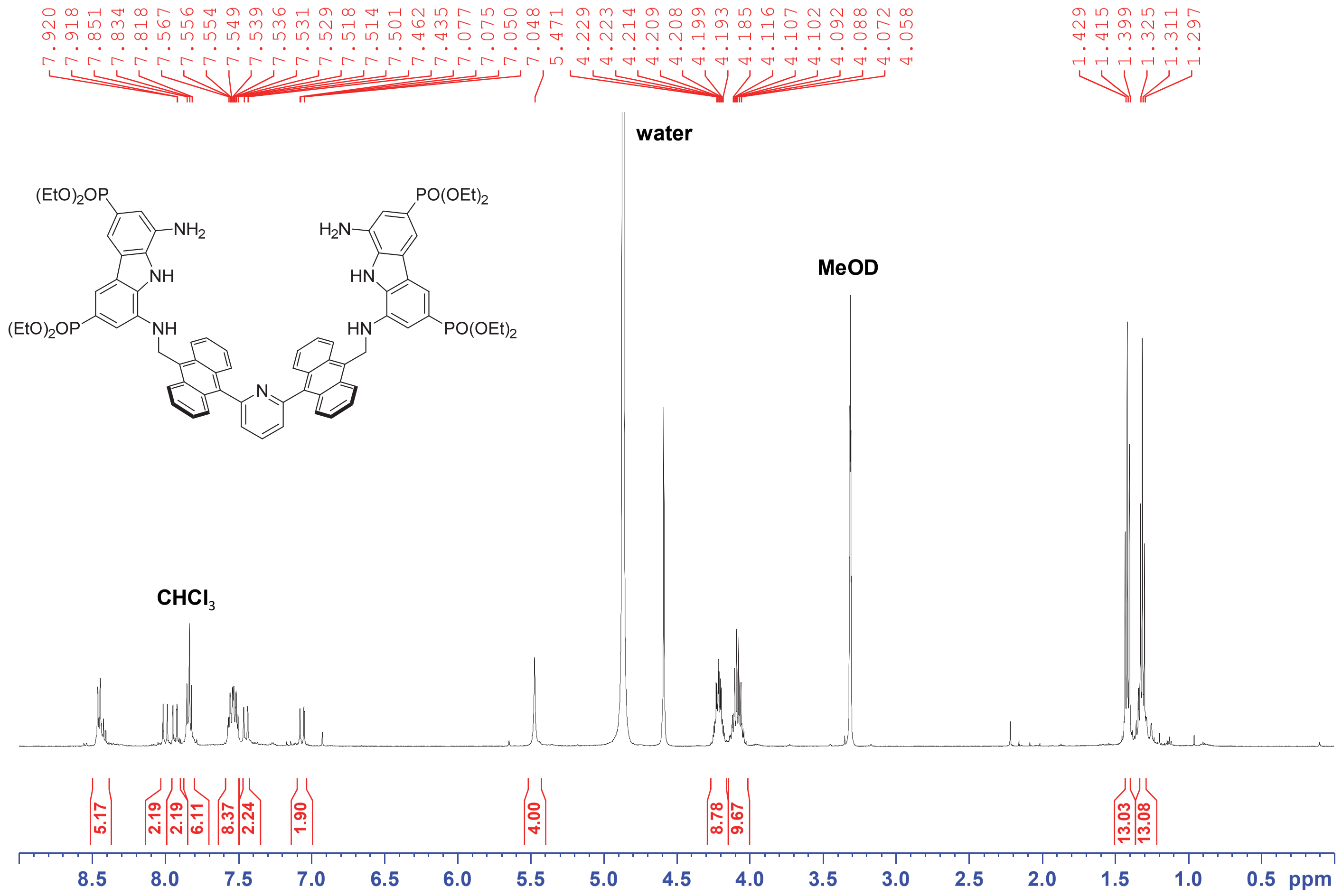

Figure S14. ^{1}H NMR spectrum of **11** (MeOD - 500 MHz).

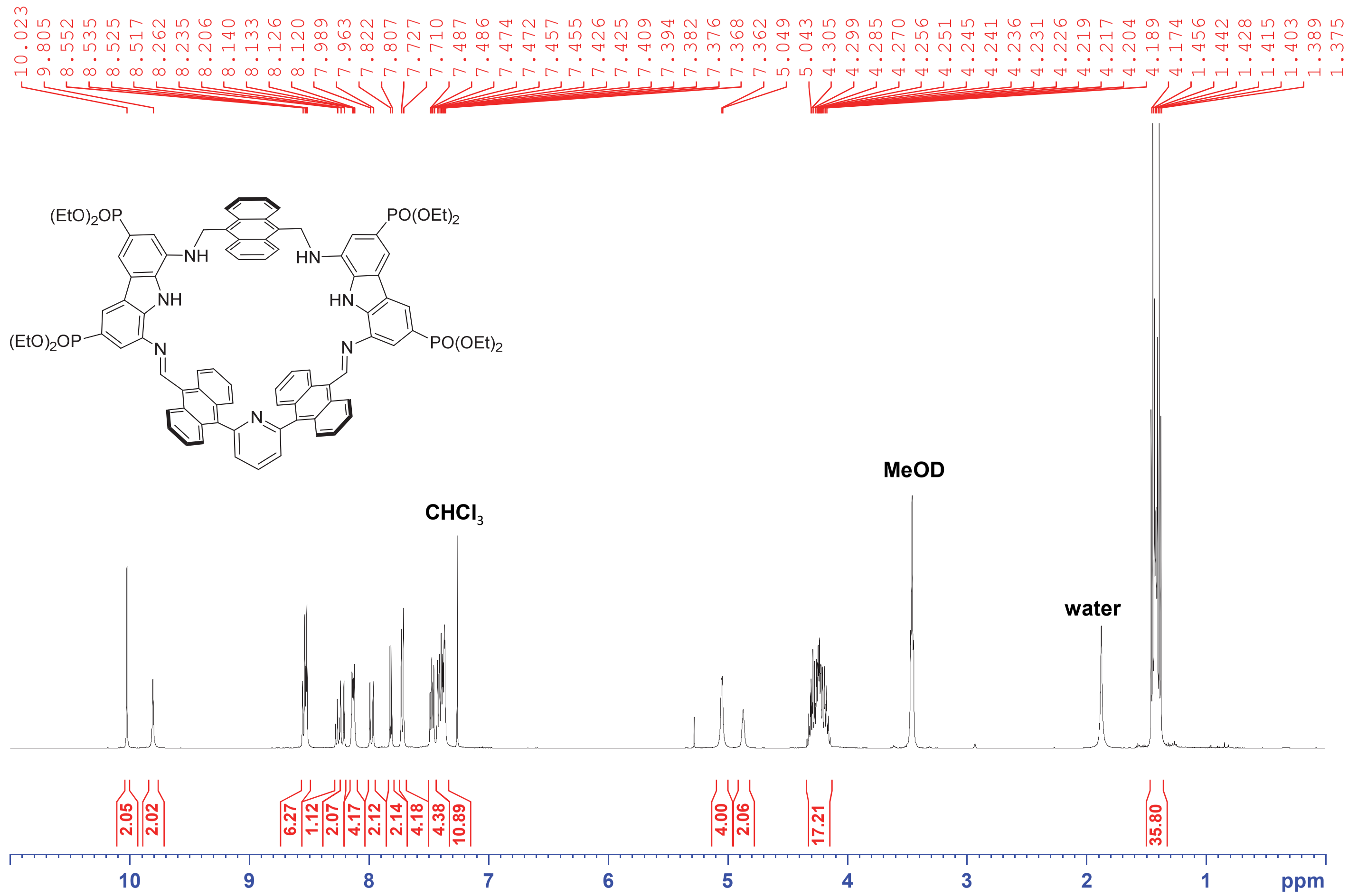

Figure S15. ^{1}H NMR spectrum of **16** ($CDCl_3$ + 10% MeOD - 500 MHz).

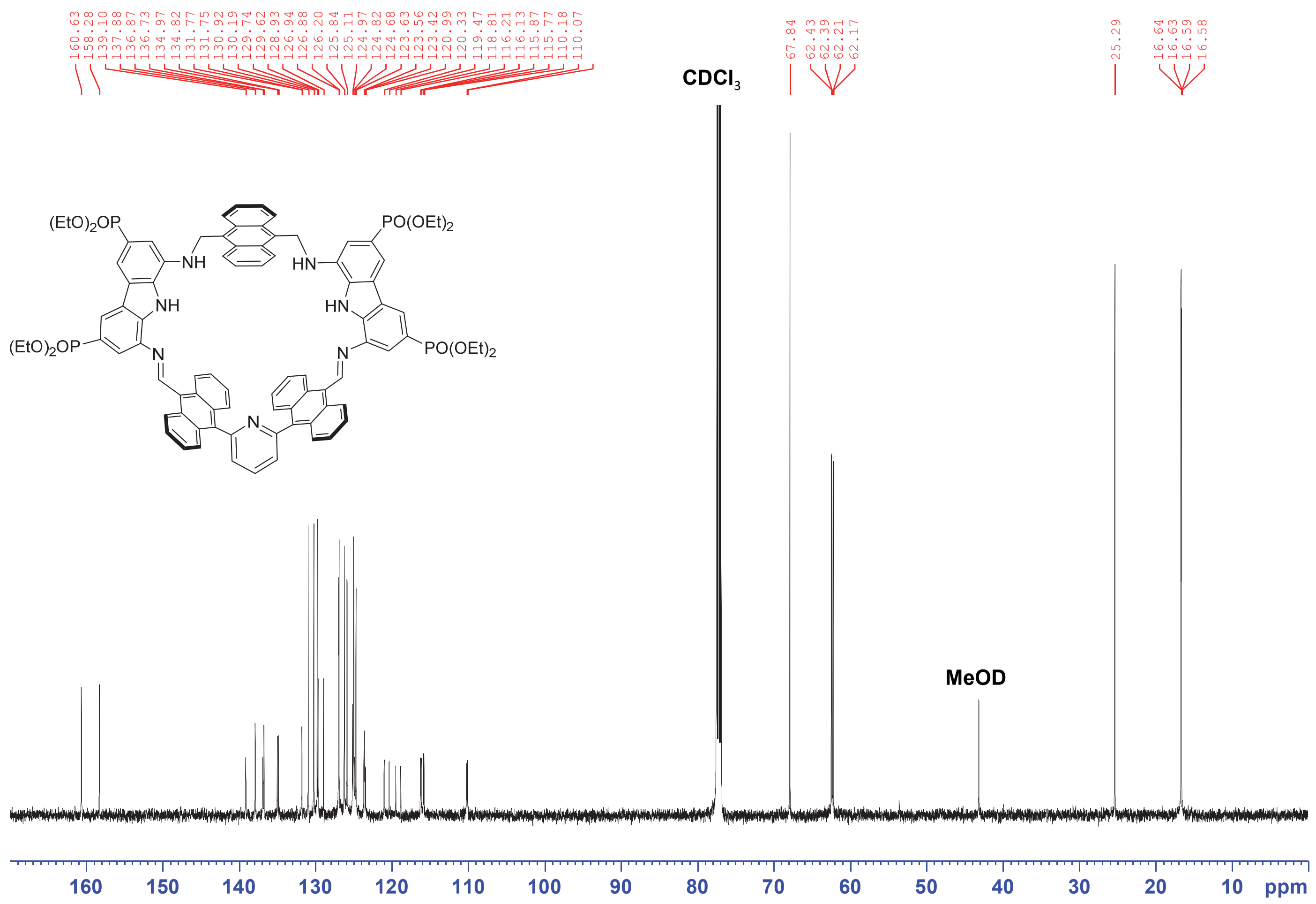

Figure S16. ^{13}C NMR spectrum of **16** ($CDCl_3$ + 10% MeOD - 125 MHz).

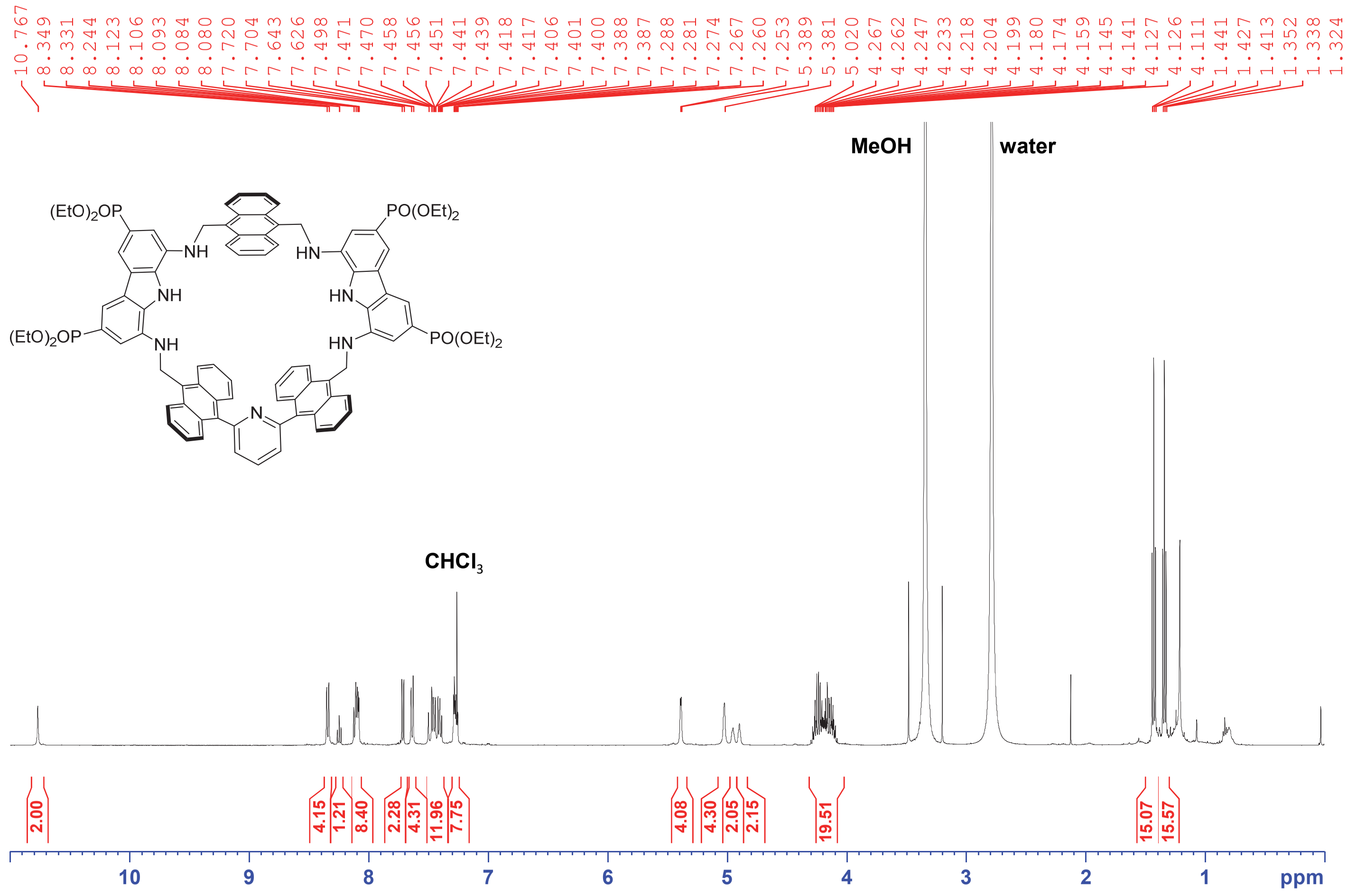

Figure S17. ^{1}H NMR spectrum of **13** ($CDCl_3$ + 5% MeOH - 500 MHz).

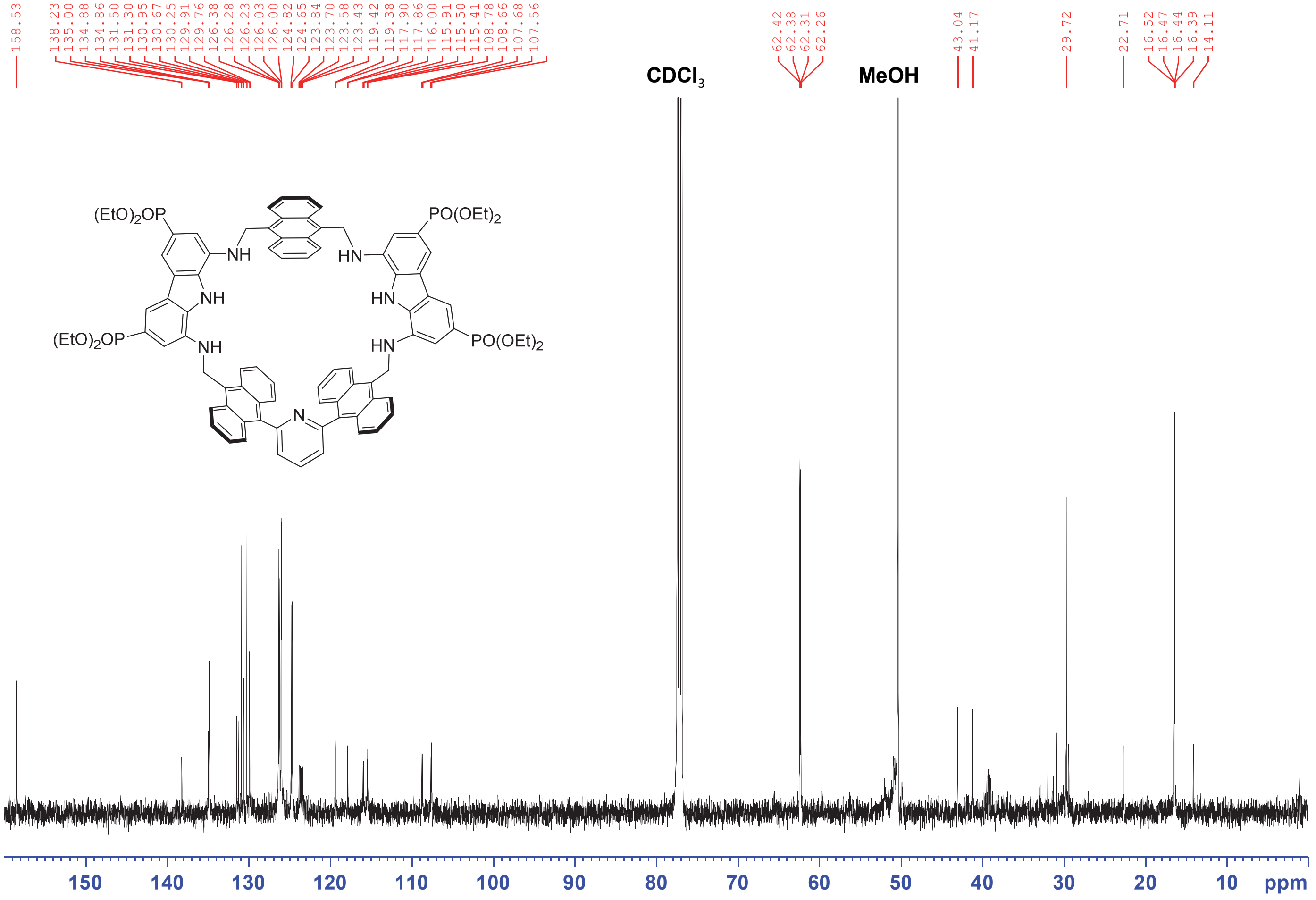

Figure S18. ^{13}C NMR spectrum of **16** ($CDCl_3$ + 5% MeOH - 125 MHz).

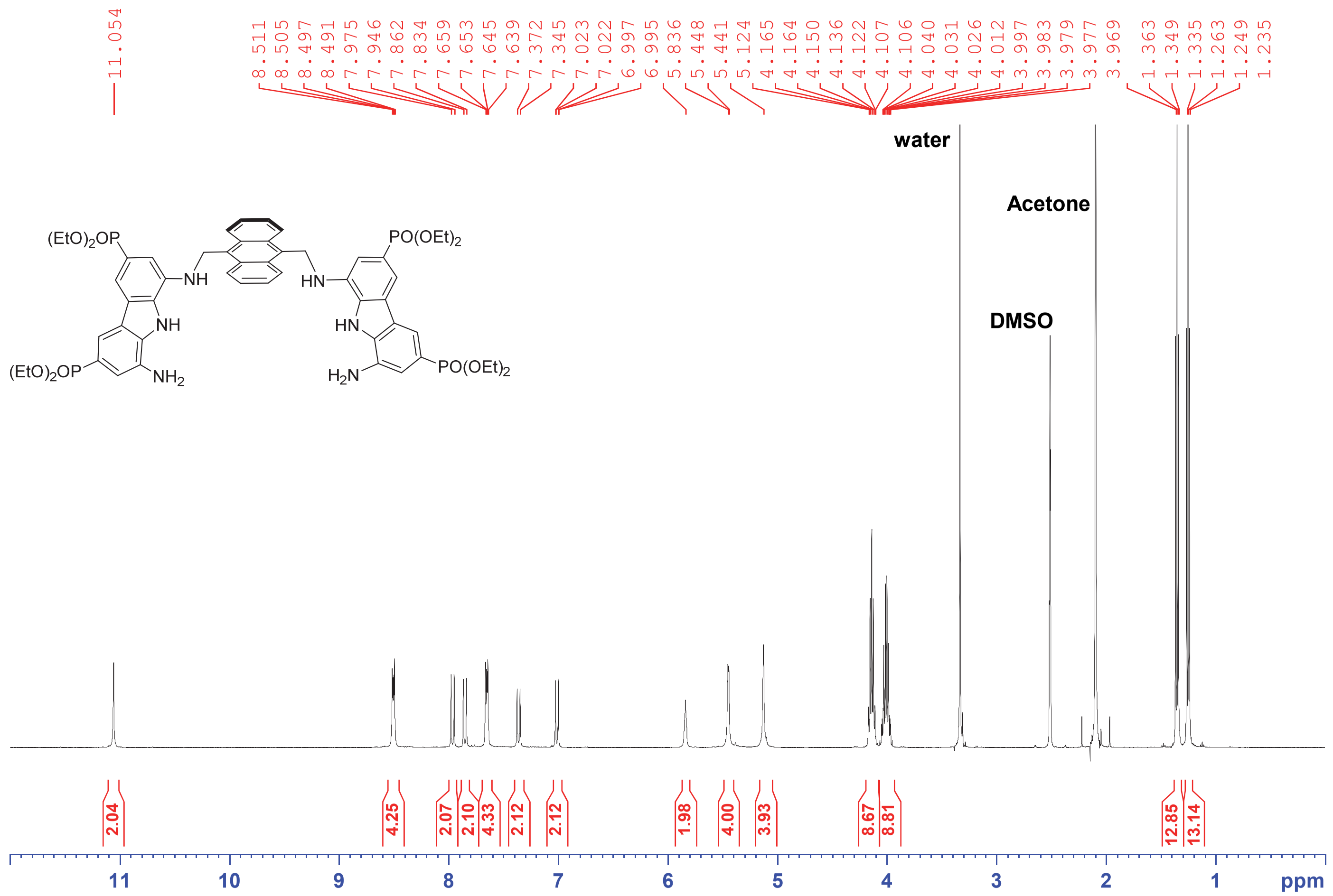

Figure S19. ^{1}H NMR spectrum of **15** (DMSO$_{d6}$ - 500 MHz).

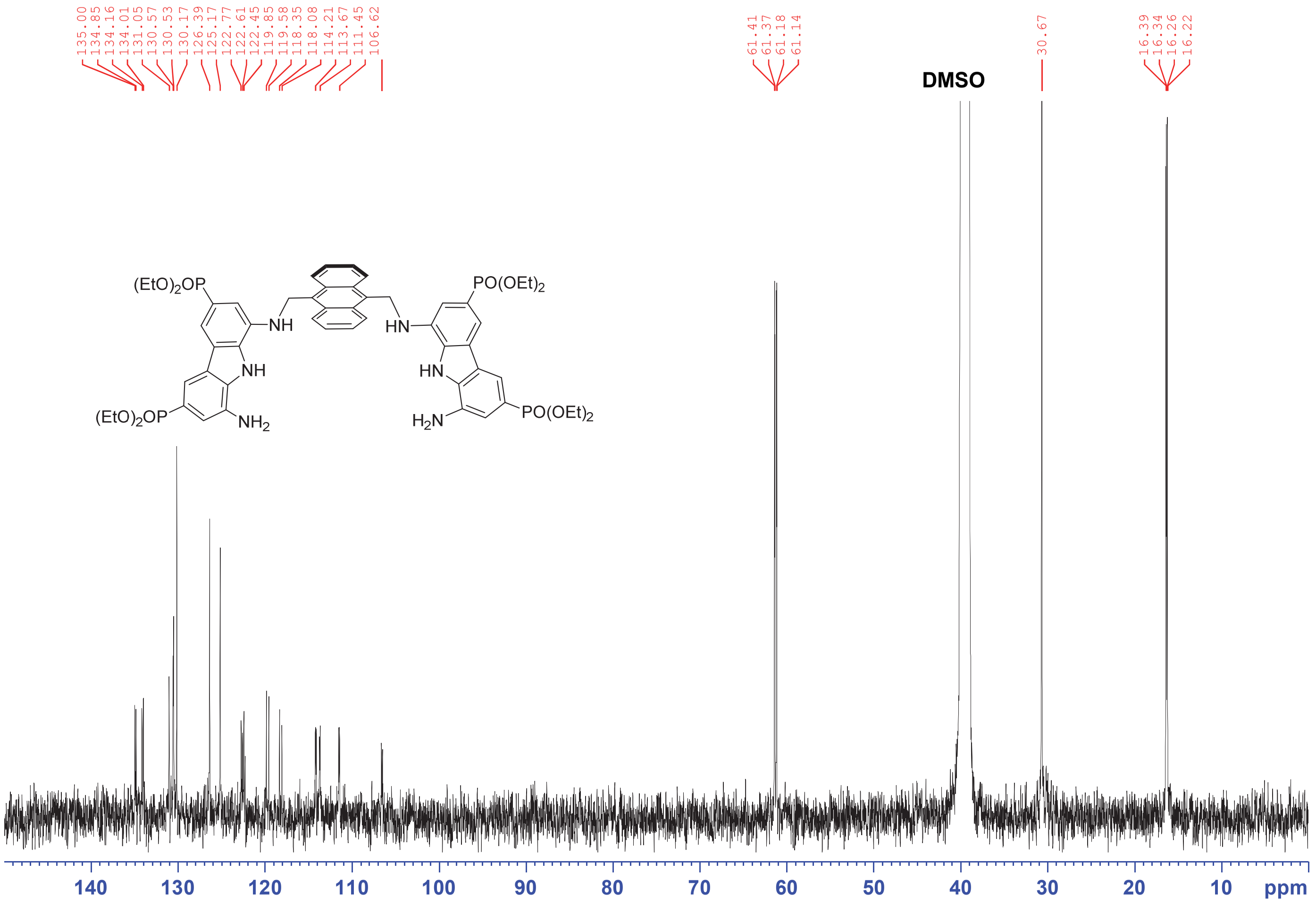

Figure S20. ^{13}C NMR spectrum of **15** (DMSO$_{d6}$ - 125 MHz).

ESI - HRMS spectra.

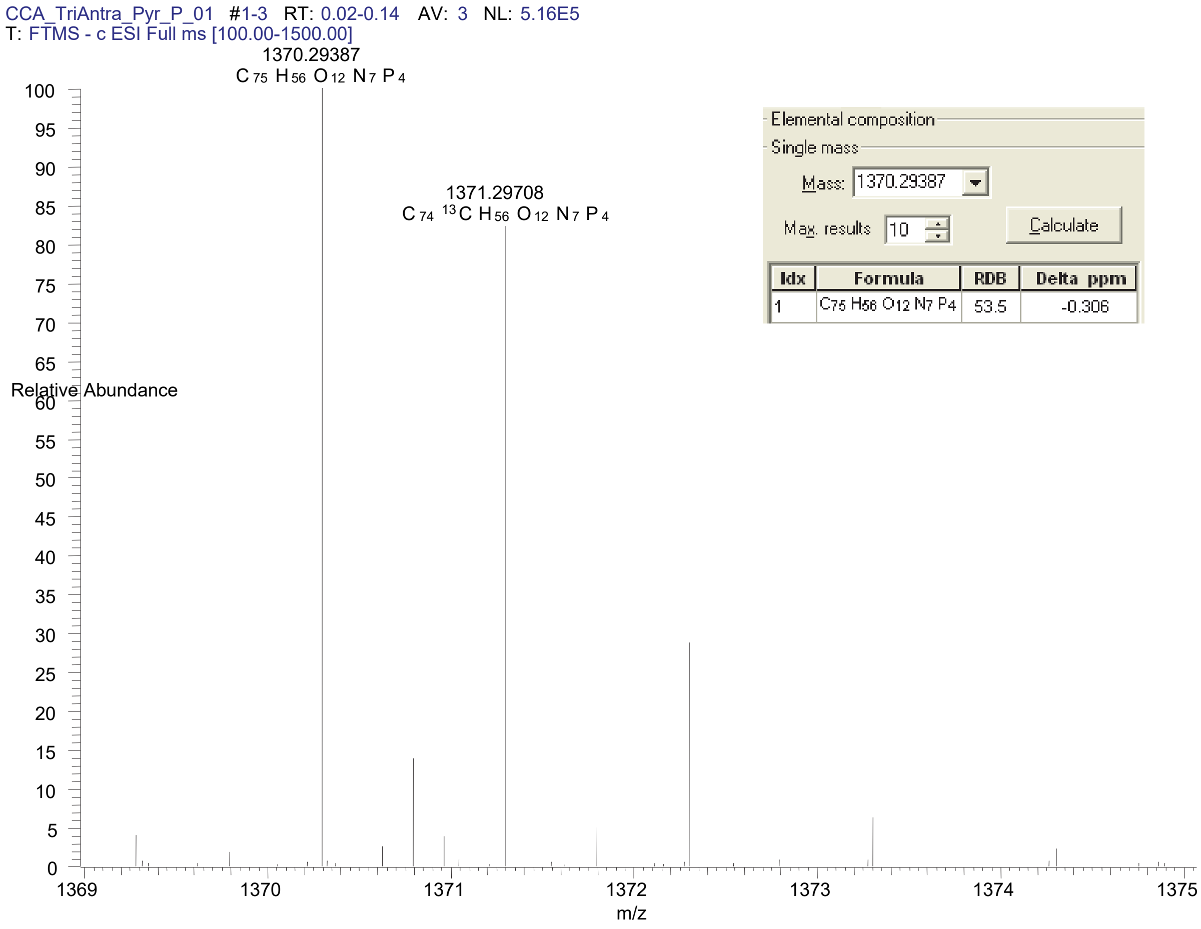

Figure S21. ESI – HRMS of **3** (negative ion mode).

Binding studies.

NMR studies of the effect of pH on the ^{1}H-NMR spectra of receptor 3.

A 1 mM solution of receptor **3** was obtained in D_2O at pD 11 and spectra were acquired for different pD values obtained for incremental additions of a diluted DCl solution in D_2O.

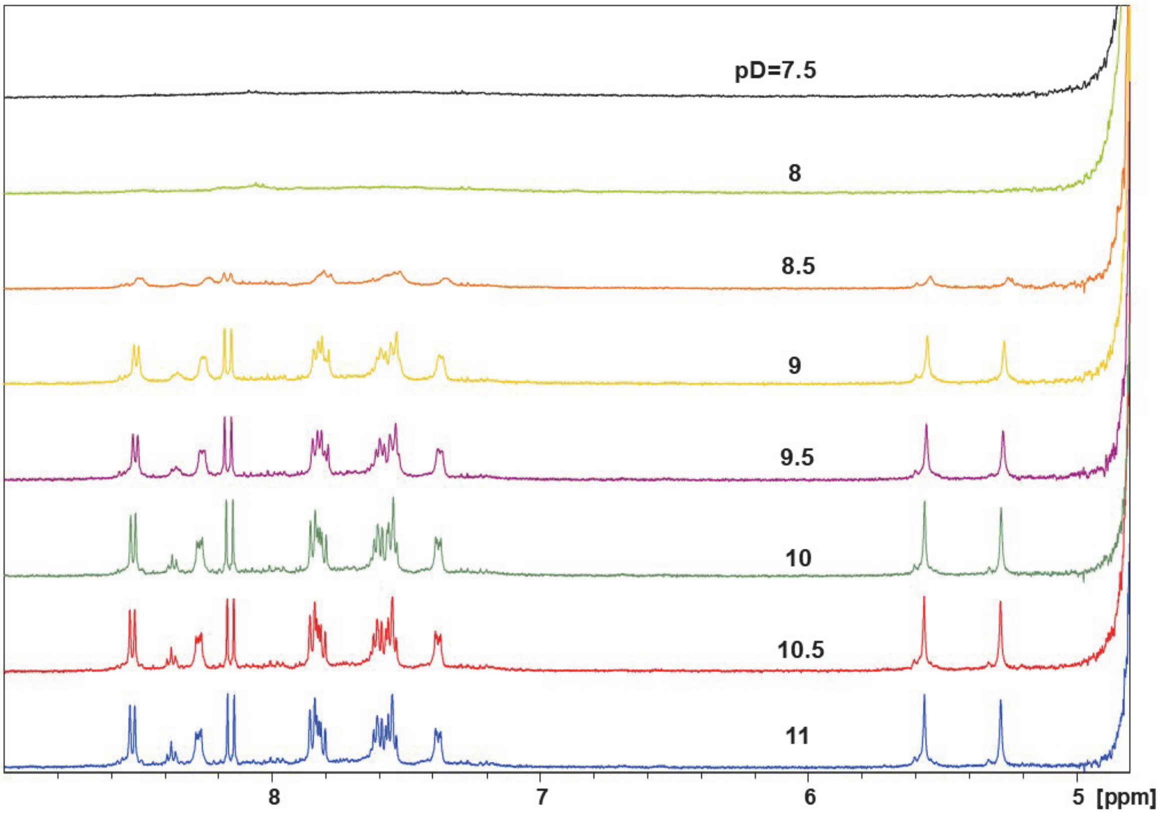

Figure S22. ^{1}H-NMR spectra (500 MHz, D_2O) of receptor **3** (1 mM) at different pD values.

NMR preliminary screening.

Preliminary screenings (298 K, 500 MHz) were performed in D_2O at pD 11 in presence of DSS as internal reference. The spectra of the free sugars at 1 mM concentration were compared to the spectra of the equimolar mixture of sugars with receptor **3** (1 mM each) and chemical shift differences were evaluated.

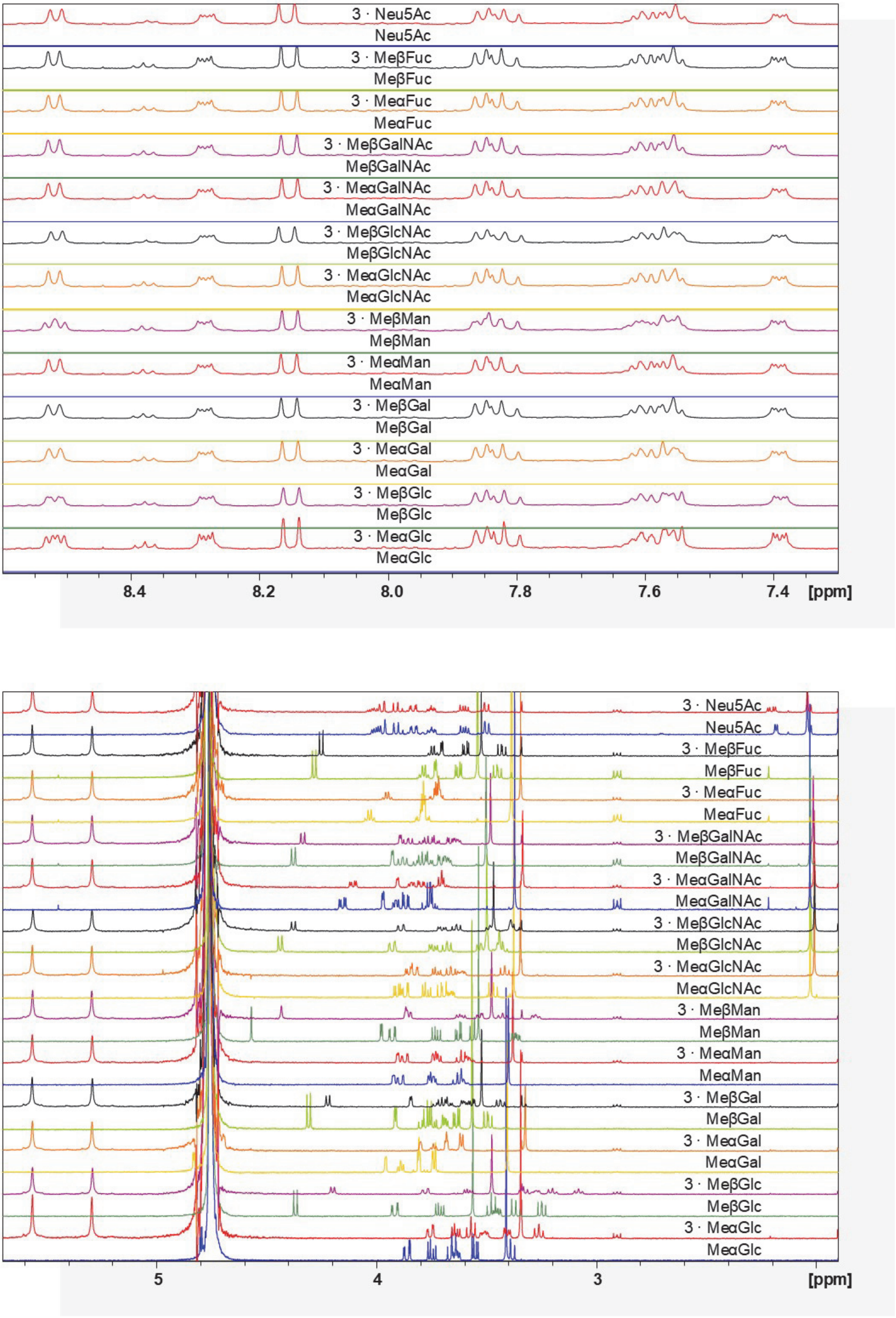

Figure S23. ^{1}H NMR spectra (500 MHz, D_2O) of a 1 mM solution of methyl glycosides and of an equimolar mixture of methyl glycosides and **3** (1 mM each) at pD 11.

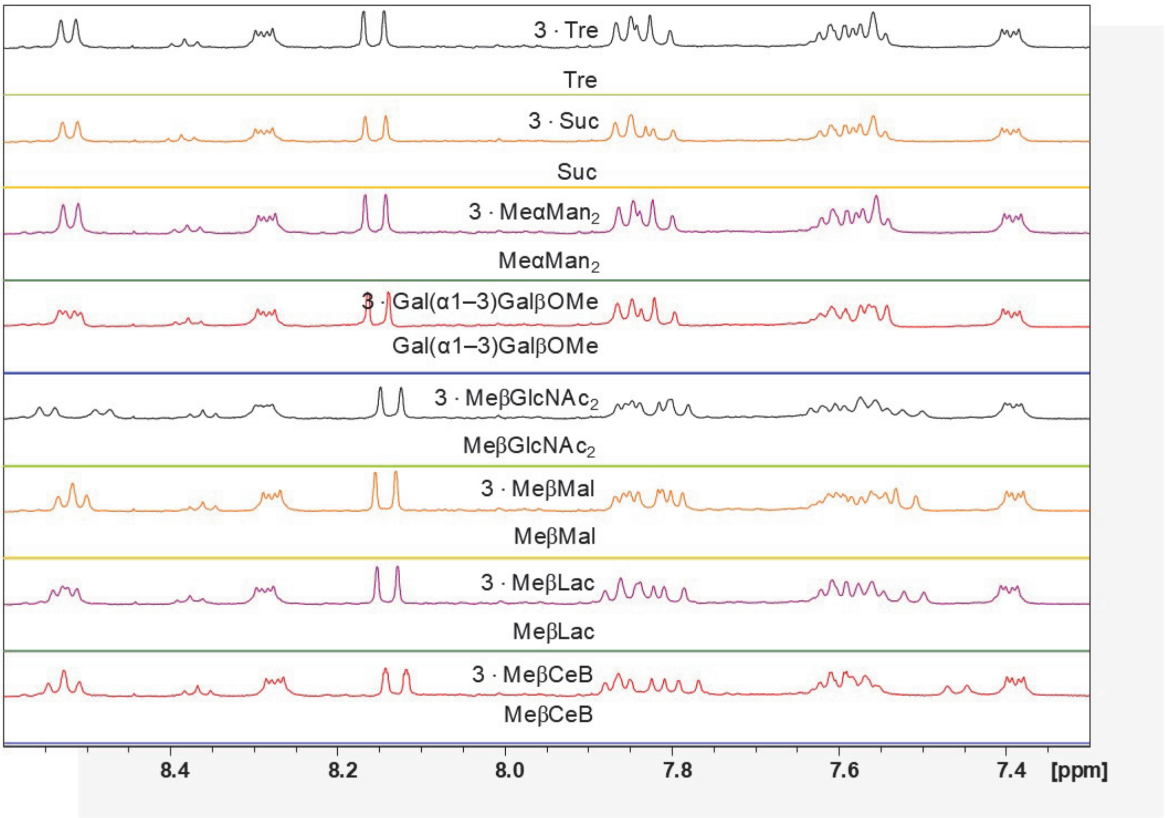

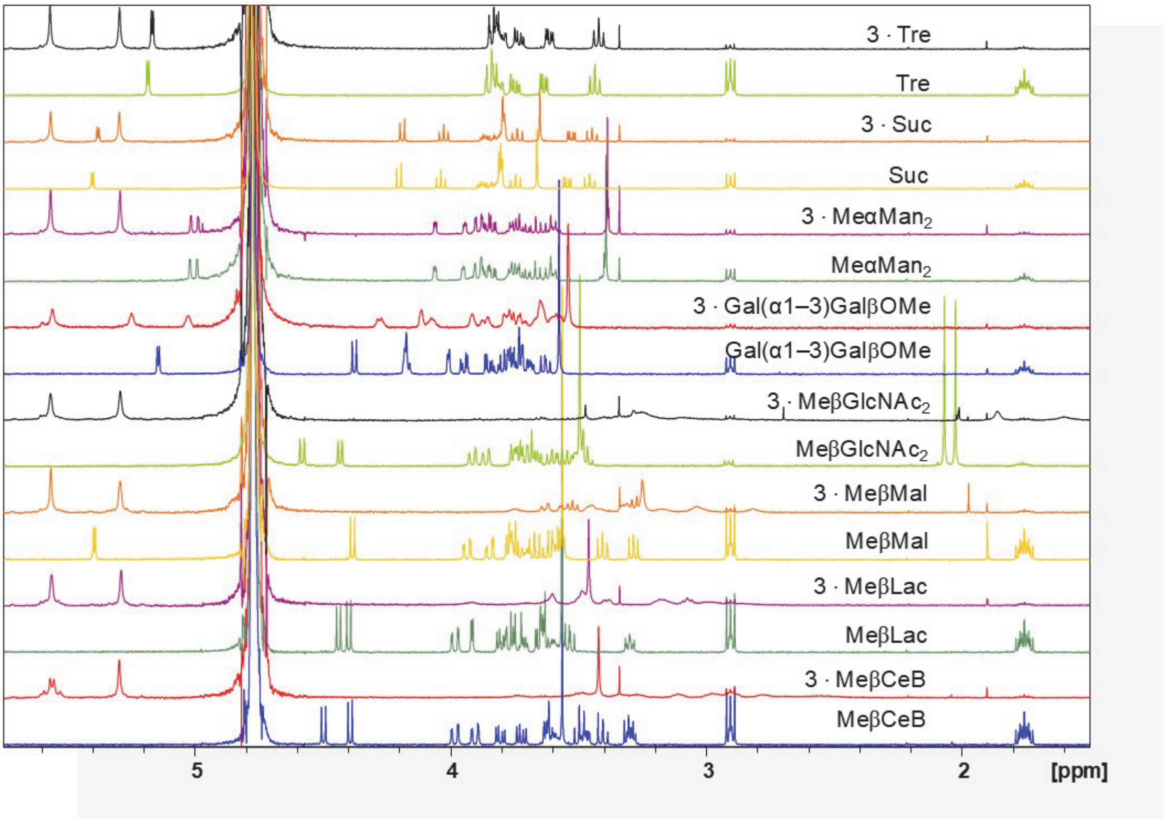

Figure S24. ^{1}H NMR spectra (500 MHz, D_2O) of a 1 mM solution of methyl glycosides and of an equimolar mixture of methyl glycosides and **3** (1 mM each) at pD 11.

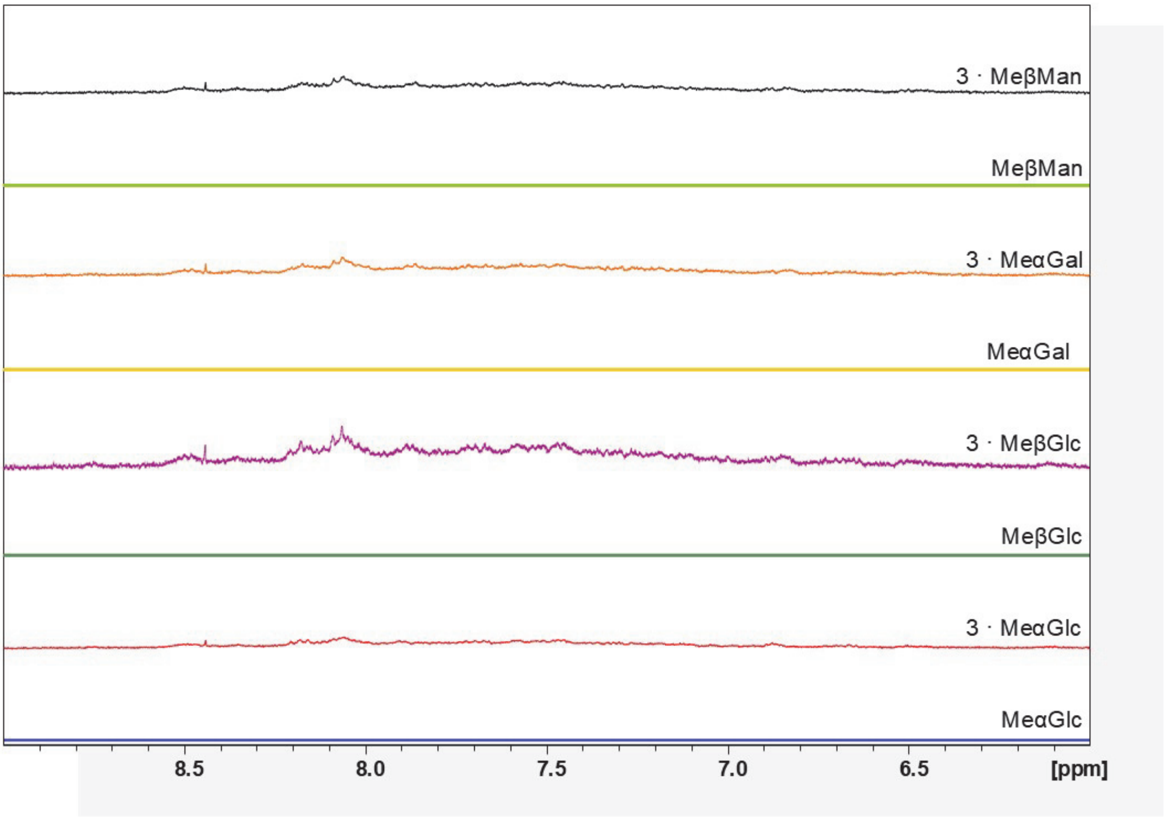

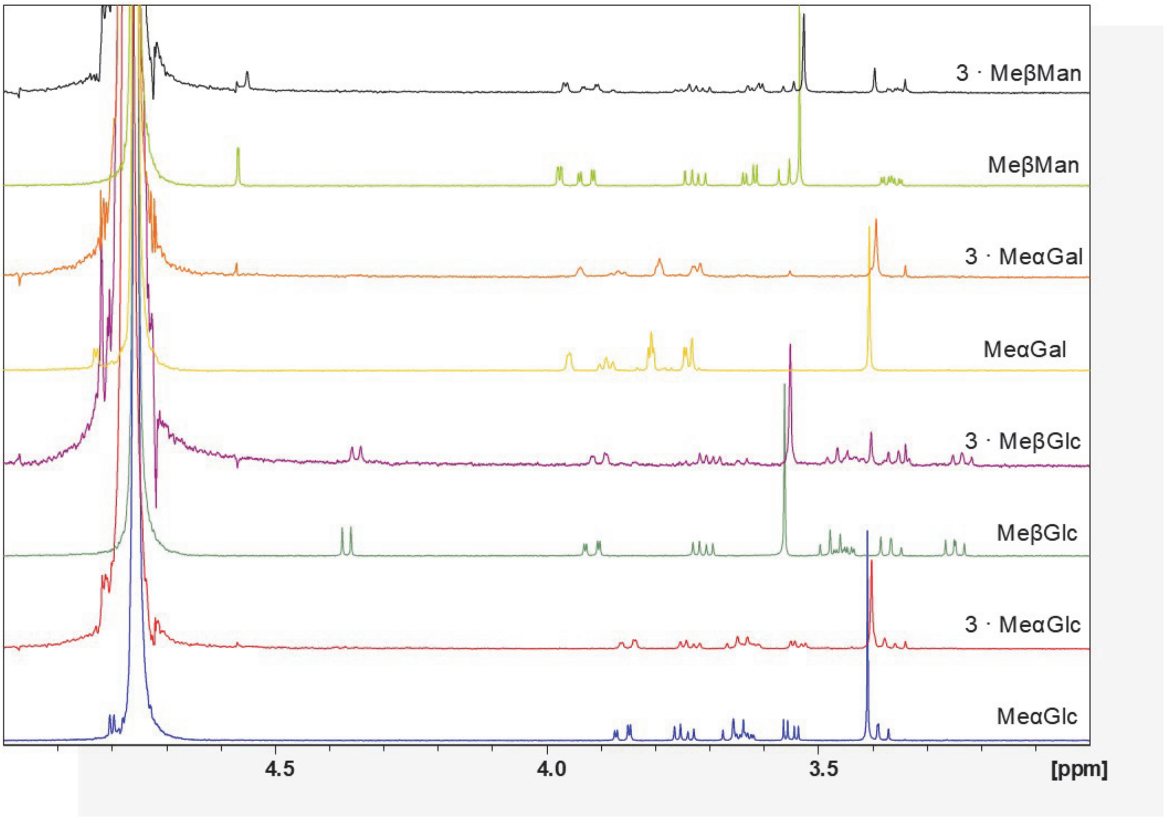

Figure S25. ^{1}H NMR spectra (500 MHz, D_2O) of a 1 mM solution of methyl glycosides and of an equimolar mixture of methyl glycosides and **3** (1 mM each) at pD 7.4.

Table S1. Chemical shift differences (Δδ) obtained by ^{1}H NMR spectra at 298 K (pD 11) of saccharide signals[a] at 1 mM upon addition of an equimolar amount of receptor **3**.

Saccharide	Δδ<0.05	0.05<Δδ<0.1	0.1<Δδ<0.2	Δδ>0.3	LB[b]
MeαGlc	-	X	-	-	-
MeβGlc	-	-	X	-	-
MeαGal	-	-	X	-	-
MeβGal	-	X	-	-	-
MeαMan	X	-	-	-	-
MeβMan	-	-	X	-	-
MeαGlcNAc	-	X	-	-	-
MeβGlcNAc	X	-	-	-	-
MeαGalNAc	X	-	-	-	-
MeβGalNAc	X	-	-	-	-
MeαFuc	-	X	-	-	-
MeβFuc	-	-	-	-	-
Neu5Ac	X	-	-	-	-
Suc	X	-	-	-	-
Tre	X	-	-	-	-
MeαMan$_2$	X	-	-	-	-
Gal(α1–3)GalβOMe	-	X	-	-	-
MeβCeB	-	-	-	X	X
MeβLac	-	-	-	X	X
MeβMal	-	-	-	X	X
MeβGlcNAc$_2$	-	-	-	X	X

[a] first available downfield signal of the carbohydrate. [b] Line broadening.

NMR studies of the effect of pH on the binding.

Starting from a solution of receptor **3** and MeβGlcNAc2 in D_2O at pD 11 spectra were acquired for different pD values by incremental additions of a diluted DCl solution in D_2O.

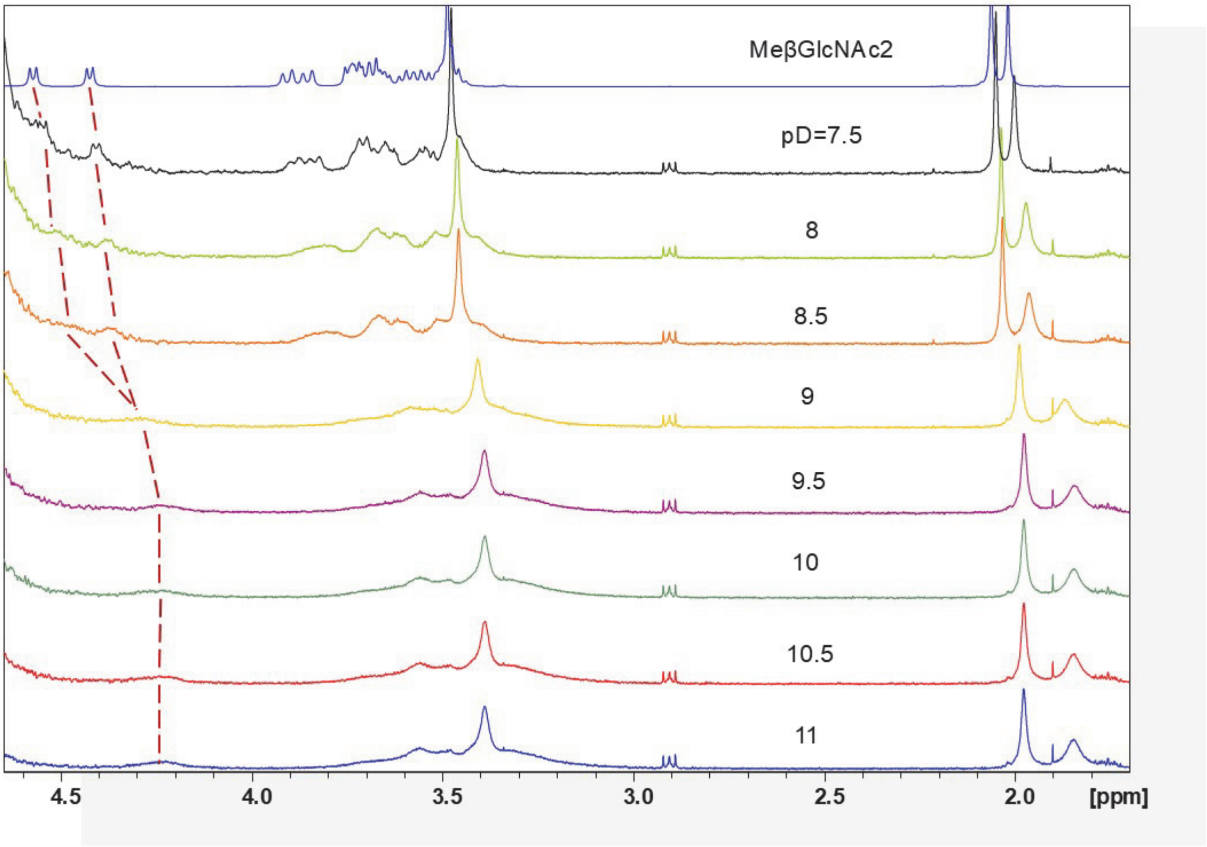

Figure S26. ^{1}H NMR spectra (500 MHz, D_2O, 298 K) of **3** (1mM) in presence of MeβGlcNAc2 (1 mM) at different pD values. Reference spectrum of MeβGlcNAc2 at pD 7.5 on top. Dotted lines show variation in chemical shifts for the anomeric protons.

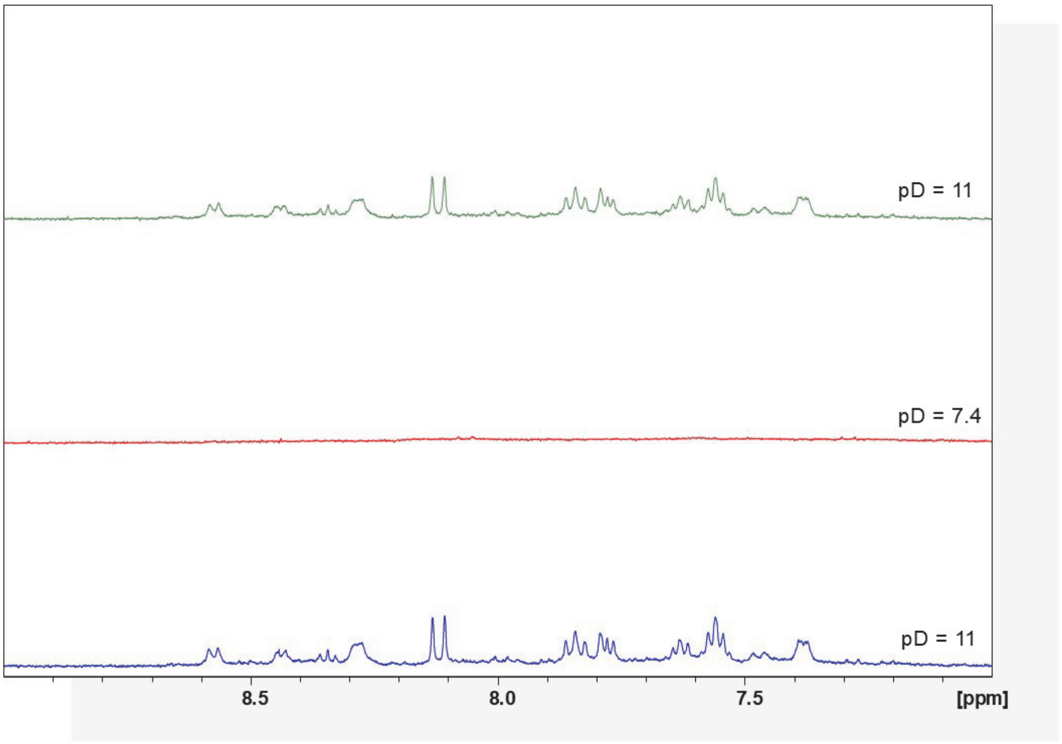

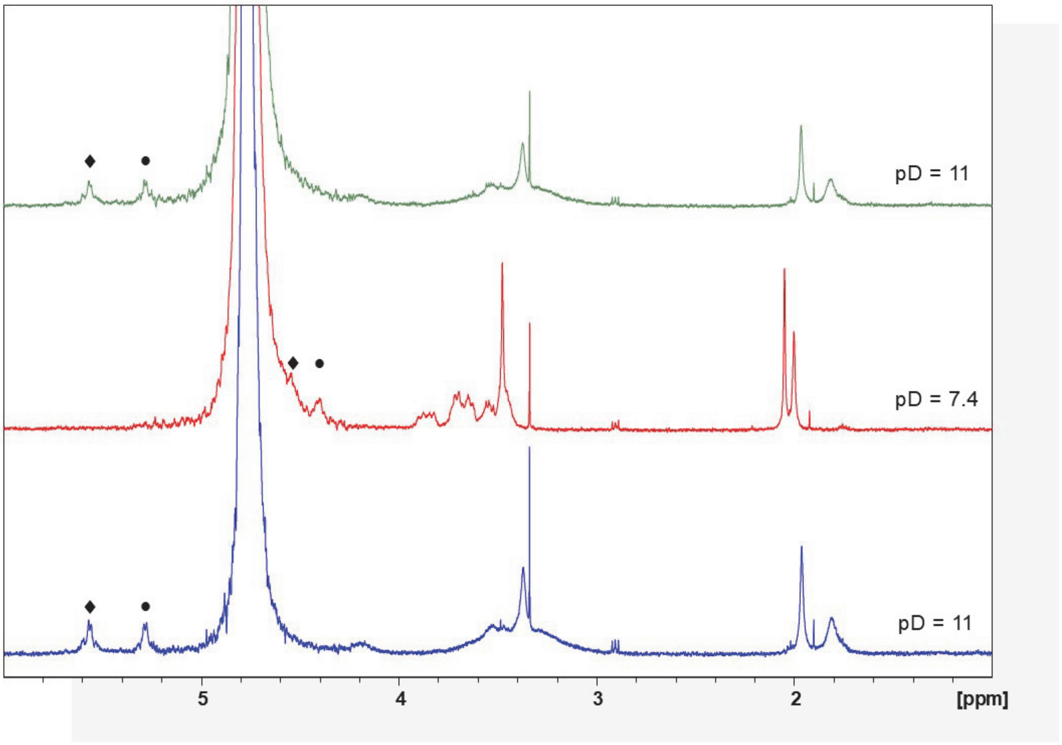

Figure S27. Binding–release–rebinding cycle followed by ^{1}H NMR spectra (500 MHz, D_2O, 298 K) of an equimolar (1mM) solution of **3** and MeβGlcNAc$_2$ changing the pD from pD 11 (top), to pD 7.4 (middle), to pD 11 (bottom). Anomeric proton signals are labelled.

NMR titrations and data analysis

Titrations were performed at 298 K, 500 MHz in 5 mm NMR tubes using microsyringes, following a previously described technique.[33] The stock solutions of **3** were prepared in D_2O adjusting the pD to a value of 7.4 with a diluted solution of NaOD. A correction factor of +0.4 was applied to the pH values measured by the pH meter to determine the pD values (pD = pH + 0.4). DSS was used as internal reference. A dilution experiment of the free receptor **3** was performed at pD 11. Data were fitted through a nonlinear least-square regression analysis, including in the fit all the available signals from both reactants. Mathematical analysis of data and graphic presentation of results was performed using the HypNMR 2006 program.[33]

Dilution of receptor 3 (D_2O, pD 11, 298 K, 500 MHz).

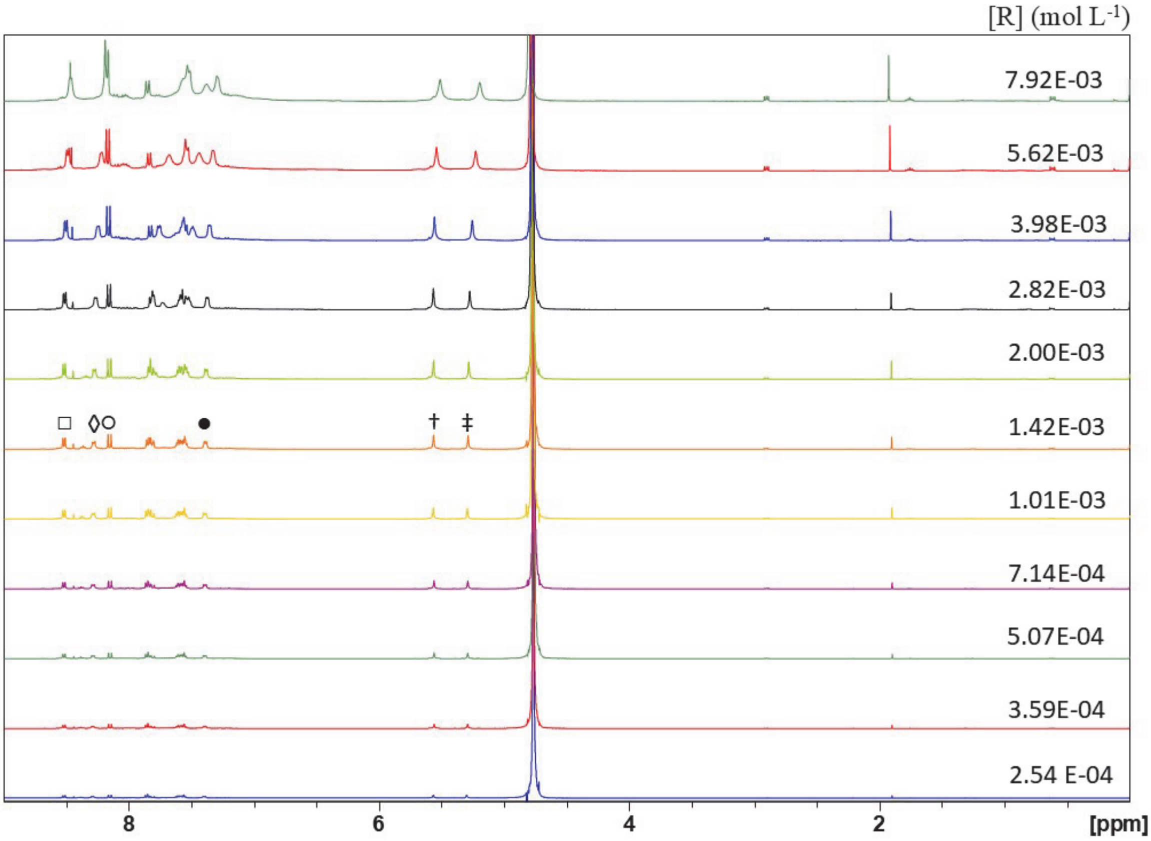

Figure S28. ^{1}H NMR spectroscopic spectra (500 MHz, D_2O, pD 11, 298 K) of receptor **3** (R) at different concentrations in dilution experiment. Followed signals are labelled (CH-F, □; CH-M, ◊; CH-I/J, ○; CH-N, ●; CH2-G, †; CH2-L, ‡).

Data Table

R = **3**

δ (ppm) *vs.* [R] (mol L^{-1})

[R]	CH-F R	CH-M R	CH-I/J R	CH-N R	CH2-G R	CH2-L R
2.54E-04	8.5207	8.2917	8.1408	7.3973	5.5646	5.2984
3.59E-04	8.5210	8.2908	8.1408	7.3969	5.5650	5.2979
5.07E-04	8.5214	8.2902	8.1408	7.3961	5.5655	5.2974
7.14E-04	8.5216	8.2891	8.1414	7.3949	5.5661	5.2963
1.01E-03	8.5216	8.2863	8.1423	7.3931	5.5669	5.2944
1.42E-03	8.5207	8.2830	8.1438	7.3901	5.5676	5.2913
2.00E-03	8.5195	8.2773	8.1458	7.3848	5.5677	5.2857
2.82E-03	8.5146	8.2663	8.1487	7.3745	5.5659	5.2749
3.98E-03	8.5057	8.2490	8.1527	7.3569	5.5593	5.2573
5.62E-03	8.4896	8.2219	8.1584	7.3299	5.5424	5.2292
7.92E-03	8.4680	-	8.1666	7.2940	5.5112	5.1936

Results page

```
 no. of spectra            11
 no. of resonance values   65
 no. of resonant nuclei     6

Chi-squared =   5.03

   sigma =   0.00048559347          RMS weighted residual =   0.00040403819

      stoich          value   relative    log    standard
      coeff                   std devn    beta   deviation

 Beta  3 refined  1.4076E+004  0.1641    4.1485   0.0713  (   R3   )
 Beta  6 refined  3.7664E+010  0.1927   10.5759   0.0837  (   R6   )

 Individual chemical shifts

                  R                       3
 =========================================================
       +       value    error         value    error
CH-F   +      8.5217   0.0002        8.5063   0.0047
CH-M   +      8.2913   0.0002        8.1840   0.0147
CH-I   +      8.1408   0.0002        8.1776   0.0047
CH-N   +      7.3972   0.0002        7.3039   0.0127
CH2-G  +      5.5648   0.0003        5.6098   0.0032
CH2-L  +      5.2985   0.0002        5.2008   0.0131
       +
                  6
 =================================
       +       value    error
CH-F   +      8.1437   0.0320
CH-M   +      7.7817   0.0573
CH-I   +      8.2363   0.0127
CH-N   +      6.8546   0.0488
CH2-G  +      5.0270   0.0426
CH2-L  +      4.7532   0.0493

 Correlation coefficients*1000


      1    2
   1
   2  856

 Parameters are numbered as follows
    1 beta 3
    2 beta 6
```

Titration Plots

Chemical shifts (δ, ppm) *vs*. concentration of R (mol L^{-1})

experimental (symbols) and calculated (lines) values

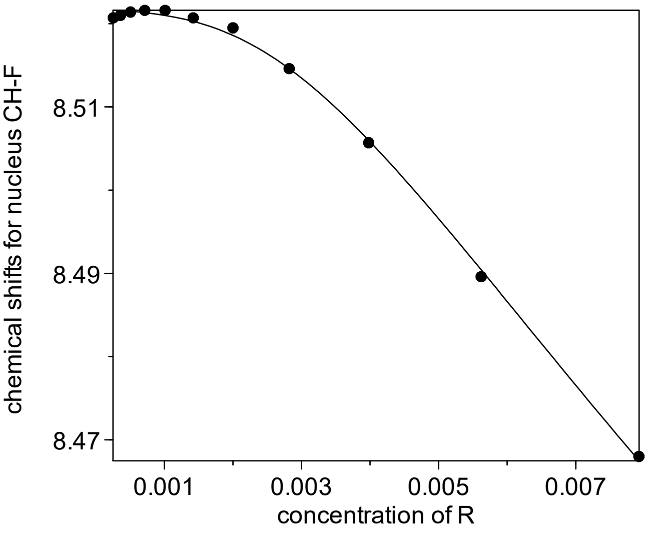

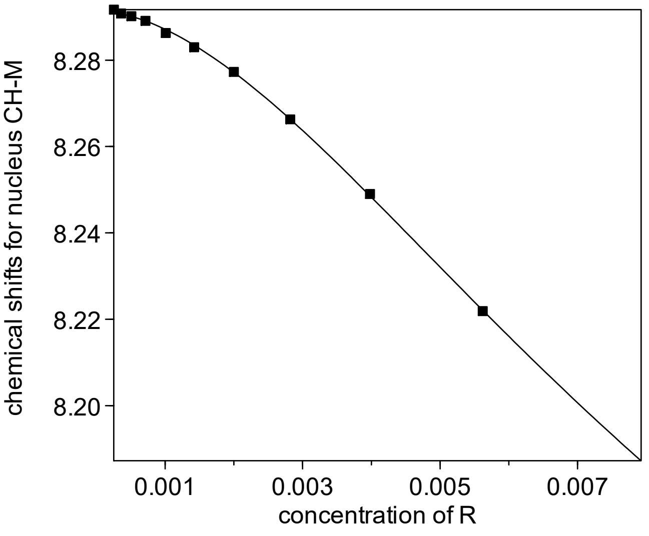

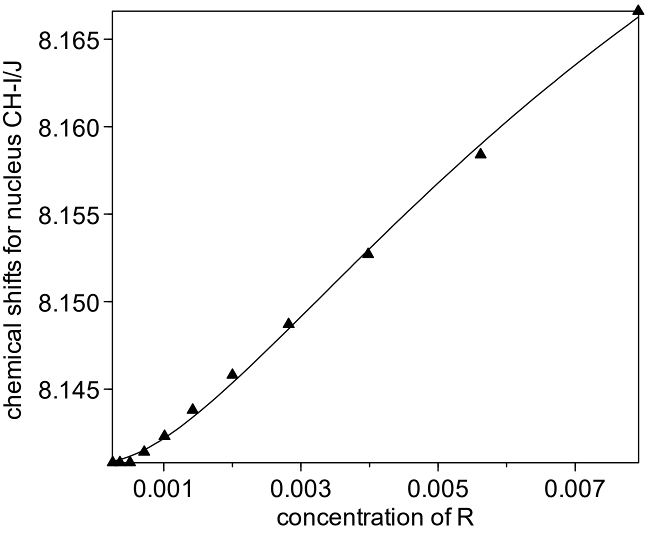

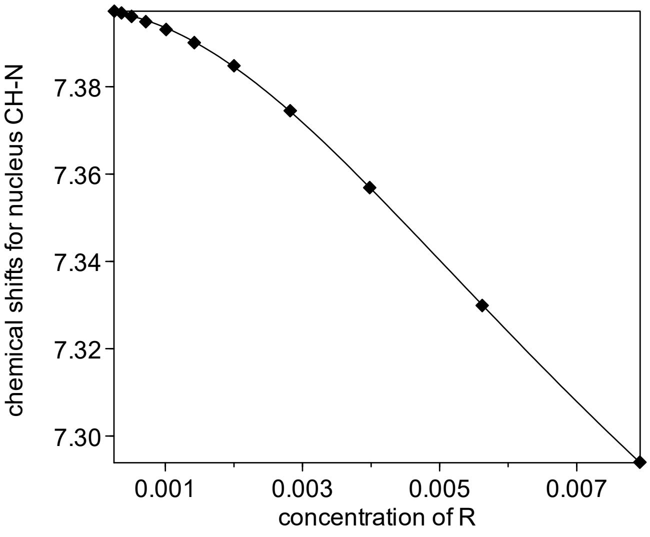

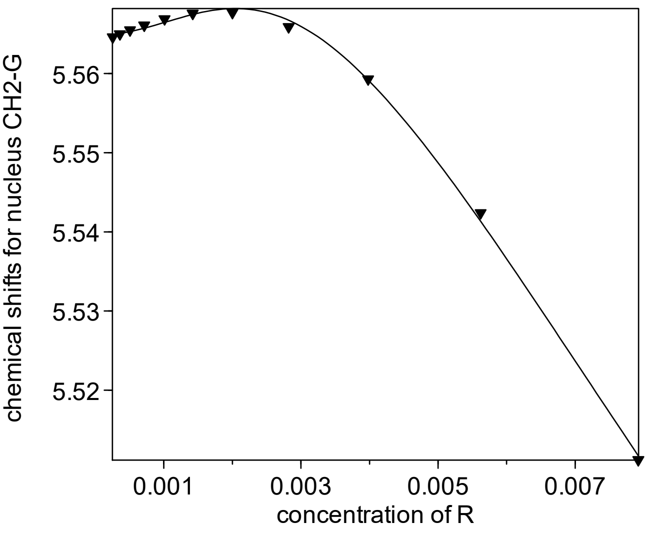

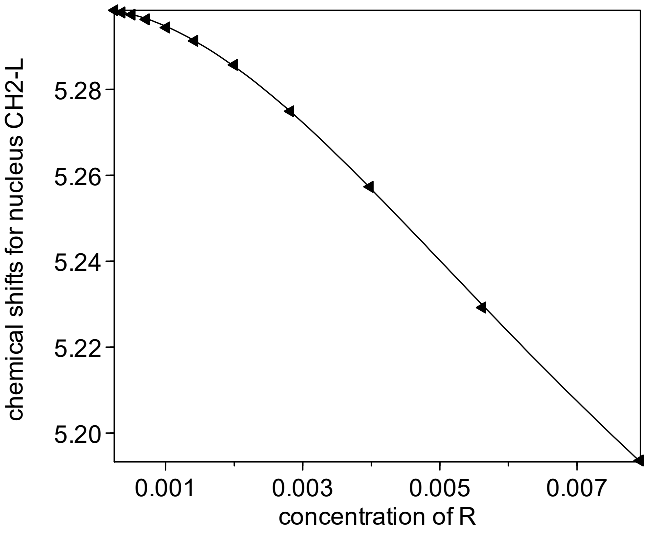

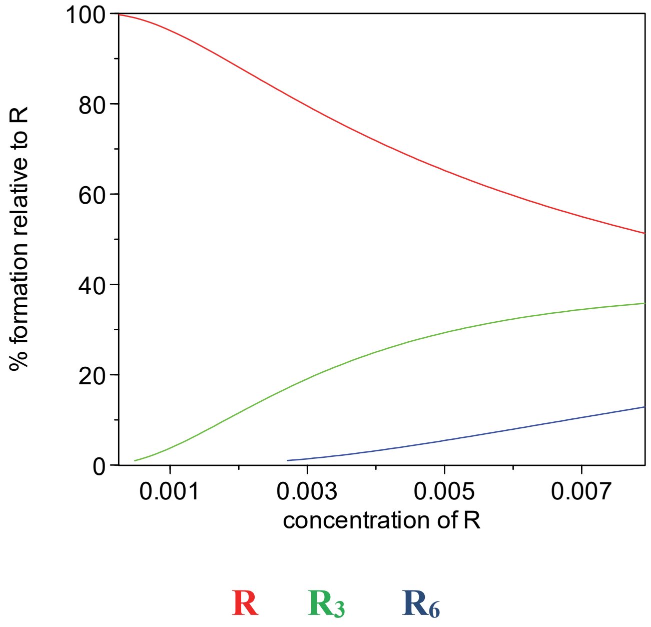

100
80
60
40
20
0
% formation relative to R
0.001
0.003
0.005
0.007
concentration of R
R
R3
R6

3 + MeβCeB (D_2O, pD 11, 298 K, 500 MHz).

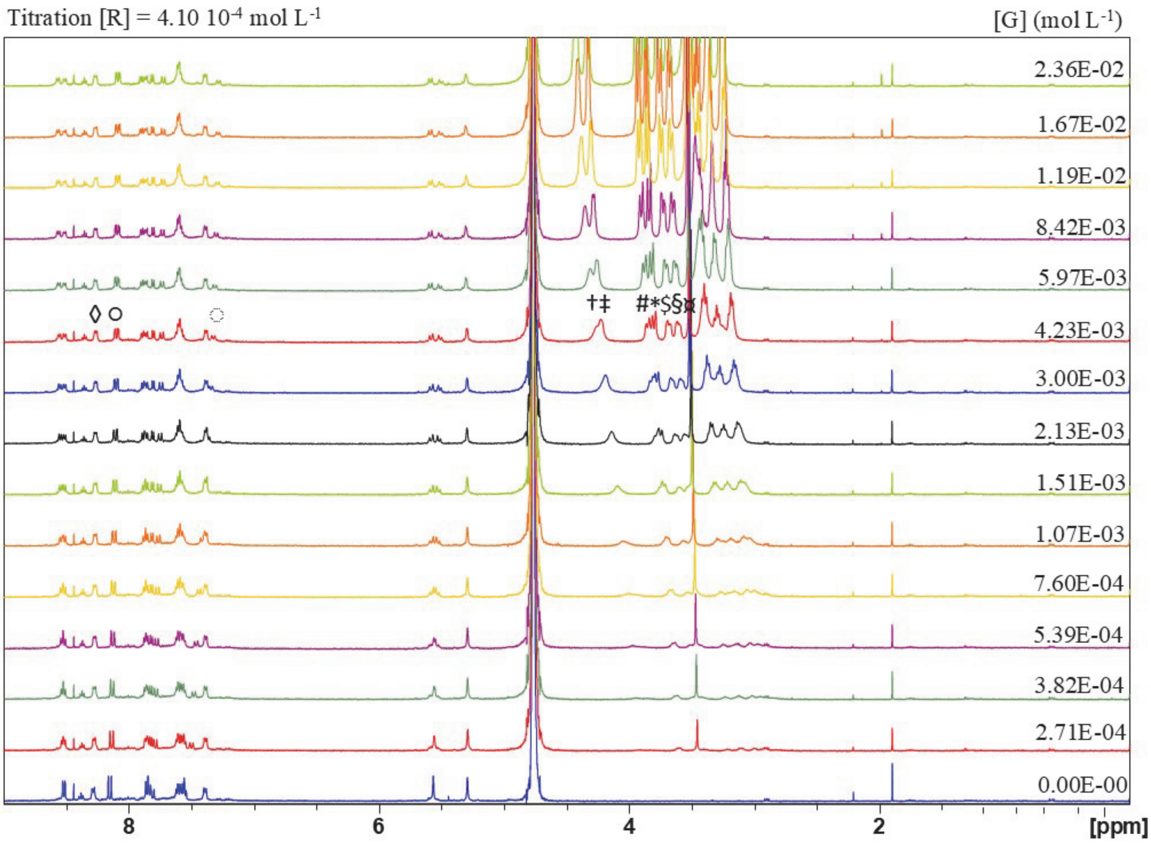

Figure S29. ^{1}H NMR spectroscopic titration (500 MHz, D_2O, pD 11, 298 K) of receptor **3** (4.10 10^{-4} mol L^{-1}) with incremental concentrations of MeβCeB (G). Followed signals are labelled (CH-M, ◊; CH-I/J, ○; CH-D, ◌; CH-1, †; CH-1', ‡; CH'-6, #; CH-6, *; CH'-6', $; CH-6', §; CH3, ¤).

Data Table

R = **3** G = MeβCeB

δ (ppm) *vs*. [G] (mol L^{-1})

Titration [R] = 4.10 10^{-4} mol L^{-1}

[G]	CH-1<br>G	CH-1'<br>G	CH'-6<br>G	CH-6<br>G	CH'-6'<br>G	CH-6'<br>G	CH3<br>G	CH-M<br>R	CH-I/J<br>R	CH-D<br>R
0.00E+00	-	-	-	-	-	-	-	8,2859	8,1662	-
2.71E-04	3,8289	3,9177	3,6203	3,5995	-	3,4023	3,4613	8,2793	8,1524	7,5141
3.82E-04	3,8661	3,9444	3,6394	3,6205	3,4955	3,4274	3,4672	8,2774	8,1481	7,4945
5.39E-04	3,9201	3,9675	3,6567	3,6384	3,5117	-	3,4730	8,2757	8,1438	7,4771
7.60E-04	3,9517	4,0092	3,6793	3,6633	3,5347	-	3,4804	8,2736	8,1385	7,4532
1.07E-03	4,0388	4,0524	3,7067	3,6914	3,5646	-	3,4894	8,2719	8,1328	7,4282
1.51E-03	4,0971	4,0955	3,7493	3,7248	3,5977	3,5297	3,4987	8,2702	8,1272	-
2.13E-03	4,1435	4,1435	3,7828	3,7536	3,6276	3,5514	3,5079	8,2688	8,1224	-
3.00E-03	4,2100	4,1897	3,8244	3,7790	3,6587	3,5847	3,5172	8,2678	8,1172	7,3593
4.23E-03	4,2625	4,2277	3,8559	3,8050	3,6871	3,6108	3,5257	8,2670	8,1127	7,3399
5.97E-03	4,3126	4,2601	3,8833	3,8257	3,7120	3,6348	3,5337	8,2667	8,1096	7,3266
8.42E-03	4,3538	4,2859	3,9049	3,8428	3,7325	3,6547	3,5395	8,2667	8,1066	7,3151
1.19E-02	4,3849	4,3089	3,9232	3,8569	3,7482	3,6696	3,5444	8,2669	8,1043	7,3062
1.67E-02	4,4118	4,3273	3,9377	3,8686	3,7612	3,6821	3,5486	8,2678	8,1031	7,3000
2.36E-02	4,4314	4,3418	3,9485	3,8777	3,7712	3,6914	3,5518	8,2691	8,1022	7,2975

Results page

```
no. of spectra            15
no. of resonance values  136
no. of resonant nuclei    10

  sigma =   0.00404315014          RMS weighted residual =   0.00371791395

      stoich            value   relative    log    standard
      coeff                     std devn    beta   deviation

Beta  1  1 refined  9.1448E+002  0.0222    2.9612   0.0096  (   GR   )

Individual chemical shifts

                  G                       R
=========================================================
       +       value    error         value    error
CH-1   +      4.4690   0.0025
CH-1'  +      4.3699   0.0023
CH'-6  +      3.9684   0.0022
CH-6   +      3.8972   0.0021
CH'-6' +      3.7887   0.0022
CH-6'  +      3.7092   0.0021
CH3    +      3.5569   0.0020
CH-M   +                              8.2813   0.0022
CH-I   +                              8.1628   0.0022
CH-D   +                              7.5522   0.0030
       +
                 1,1
=================================
       +       value    error
CH-1   +      1.8445   0.0416
CH-1'  +      2.4970   0.0309
CH'-6  +      2.4942   0.0255
CH-6   +      2.6836   0.0221
CH'-6' +      2.4842   0.0234
CH-6'  +      2.4488   0.0242
CH3    +      3.1604   0.0137
CH-M   +      8.2646   0.0018
CH-I   +      8.0986   0.0018
CH-D   +      7.2826   0.0020
```

Titration Plots

Chemical shifts (δ, ppm) *vs*. concentration of G (mol L^{-1})

experimental (symbols) and calculated (lines) values

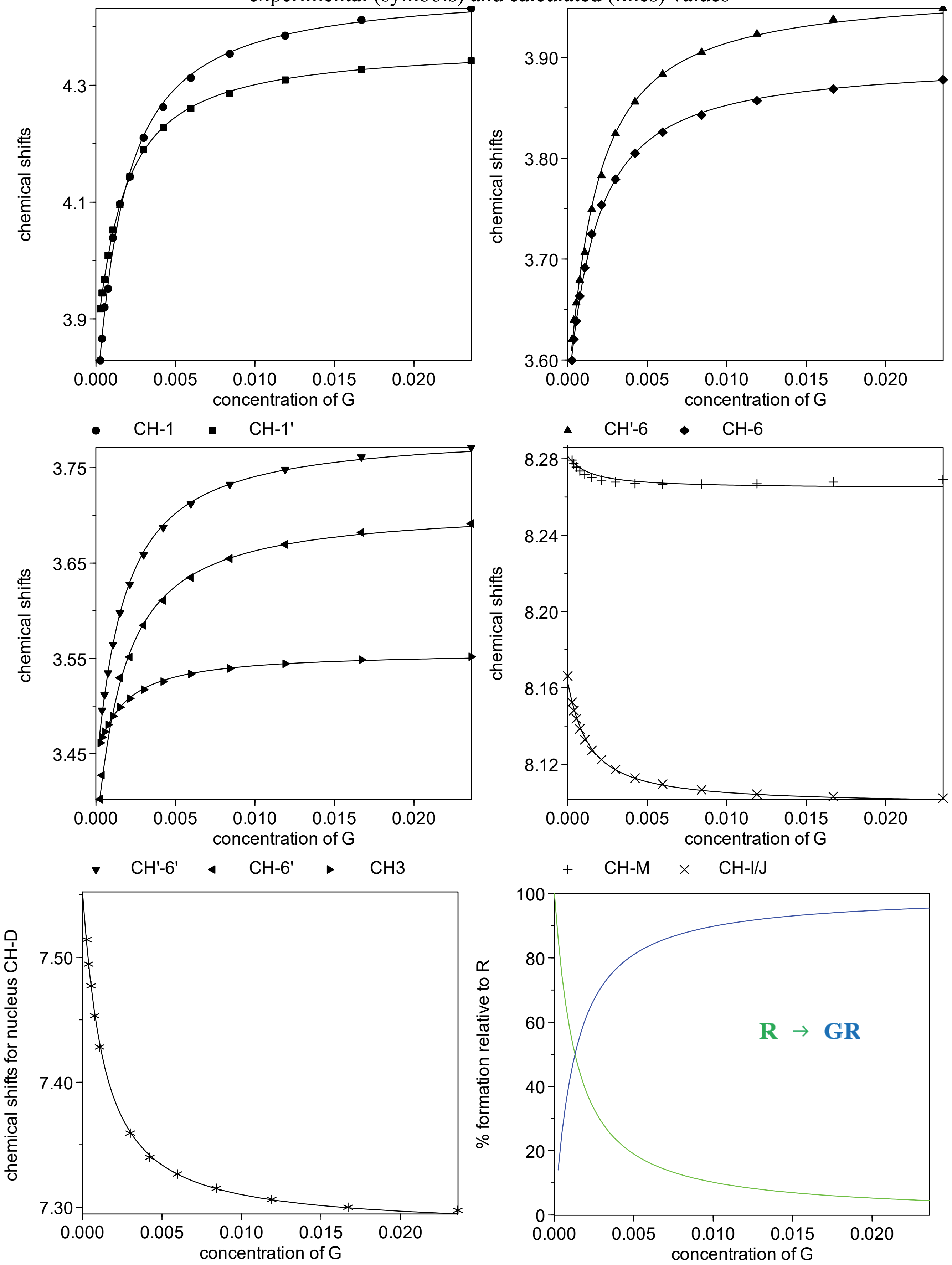

3 + MeβCeB (D_2O, pD 7.4, 298 K, 500 MHz).

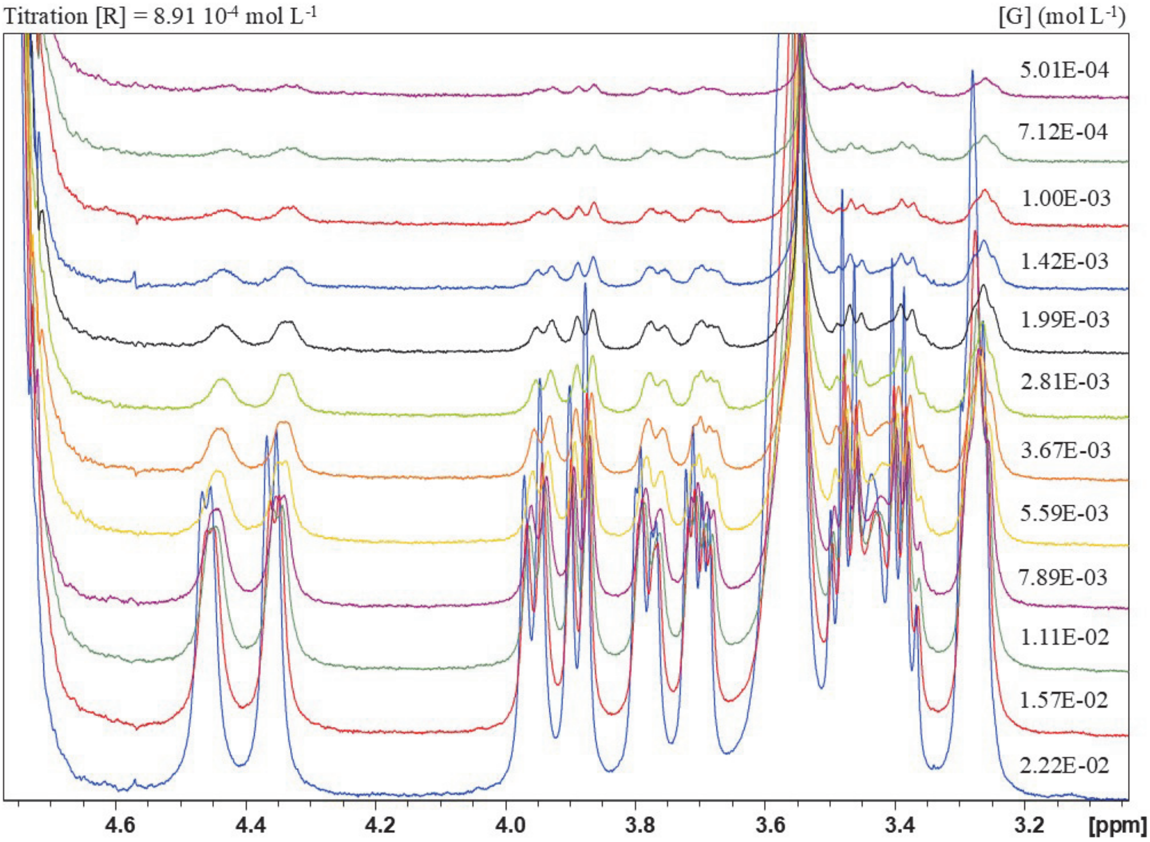

Figure S30. ^{1}H NMR spectroscopic titration (500 MHz, D_2O, pD7.4, 298 K) of receptor **3** (8.91 10^{-4} mol L^{-1}) with incremental concentrations of MeβCeB (G).

3 + MeβGlcNAc2 (D_2O, pD 11, 298 K, 500 MHz).

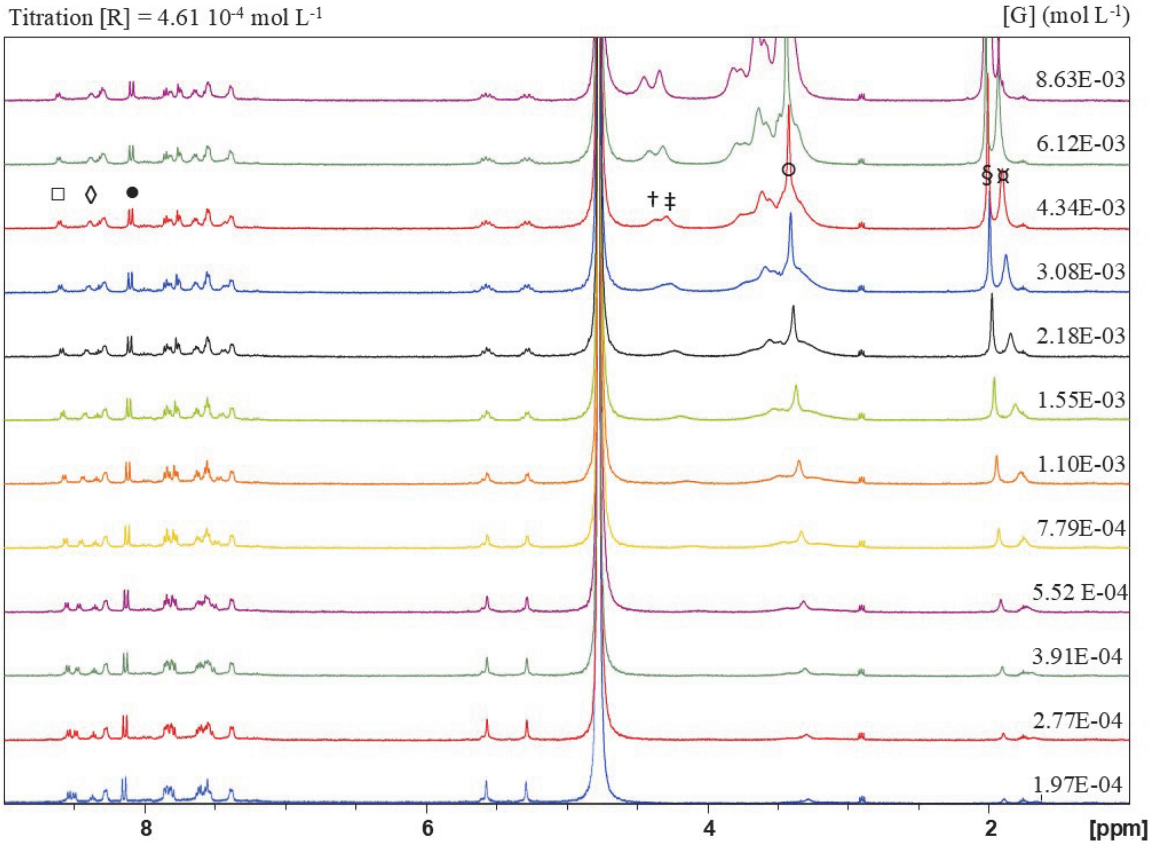

Figure S31. ^{1}H NMR spectroscopic titration (500 MHz, D_2O, pD 11, 298 K) of receptor **3** (4.61 10^{-4} mol L^{-1}) with incremental concentrations of MeβGlcNAc2 (G). Followed signals are labelled (CH-F, □; CH-F', ◊; CH-I/J, ●; CH'-1, †; CH-1, ‡; CH3, ○; Ac', §; Ac, ¤).

Data Table

R = **3** G = MeβGlcNAc2

δ (ppm) *vs.* [G] (mol L^{-1})

Titration [R] = 4.61 10^{-4} mol L^{-1}

[G]	CH-F<br>R	CH-F'<br>R	CH-I/J<br>R	CH-1'<br>G	CH-1<br>G	CH3<br>G	Ac'<br>G	Ac<br>G
0.00E+00	8.5204	8.5204	8.1661	-	-	-	-	-
1.97E-04	8.5357	8.4958	8.1567	-	-	3.2898	1.8902	1.6628
2.78E-04	8.5407	8.4902	8.1535	-	-	3.2977	1.8972	1.6807
3.92E-04	8.5485	8.4804	8.1496	-	4.0467	3.3072	1.9061	1.7004
5.52E-04	8.5543	8.4693	8.1450	-	4.0570	3.3182	1.9185	1.7389
7.79E-04	8.5650	8.4543	8.1390	-	4.1069	3.3353	1.9317	1.7567
1.10E-03	8.5738	8.4412	8.1334	-	4.1492	3.3521	1.9466	1.7768
1.55E-03	8.5837	8.4268	8.1277	-	4.1914	3.3717	1.9638	1.8134
2.18E-03	8.5939	8.4140	8.1218	4.2463	4.2256	3.3897	1.9798	1.8461
3.08E-03	8.6037	8.4008	8.1168	4.3085	4.2593	3.4080	1.9953	1.8792
4.34E-03	8.6095	8.3926	8.1130	4.3725	4.2921	3.4246	2.0094	1.9069
6.12E-03	8.6153	8.3856	8.1098	4.4166	4.3189	3.4380	2.0209	1.9325
8.63E-03	8.6178	8.3827	8.1067	4.4528	4.3434	3.4488	2.0308	1.9518

Results page

```
no. of spectra            13
no. of resonance values   90
no. of resonant nuclei     8

  sigma =   0.00345477498          RMS weighted residual =   0.00311142933

      stoich            value   relative    log    standard
      coeff                     std devn    beta   deviation

Beta  1  1 refined  1.2475E+003  0.0469    3.0960   0.0204  (   GR   )

Individual chemical shifts

                  G                       R
=========================================================
       +       value    error         value    error
CH-F   +                              8.5199   0.0020
CH-F'  +                              8.5191   0.0021
CH-I   +                              8.1661   0.0019
CH-1'  +      4.5556   0.0047
CH-1   +      4.3997   0.0034
CH3    +      3.4740   0.0026
Ac'    +      2.0532   0.0025
Ac     +      1.9957   0.0032
       +
                 1,1
=================================
       +       value    error
CH-F   +      8.6271   0.0021
CH-F'  +      8.3677   0.0023
CH-I   +      8.1021   0.0020
CH-1'  +      2.4521   0.0538
CH-1   +      3.2142   0.0361
CH3    +      2.9156   0.0193
Ac'    +      1.5625   0.0174
Ac     +      1.0121   0.0317
```

Titration Plots

Chemical shifts (δ, ppm) *vs*. concentration of G (mol L^{-1})

experimental (symbols) and calculated (lines) values

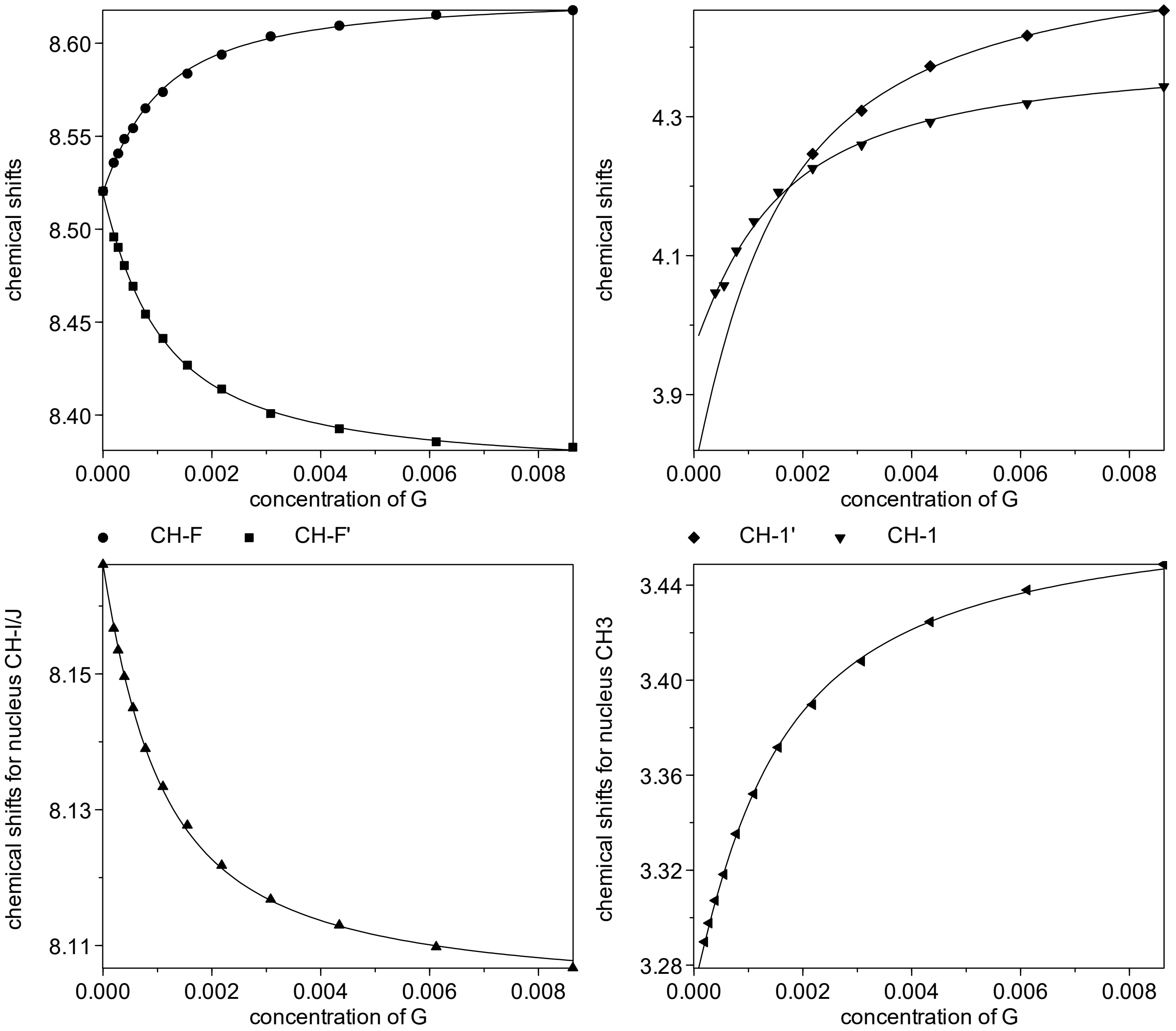

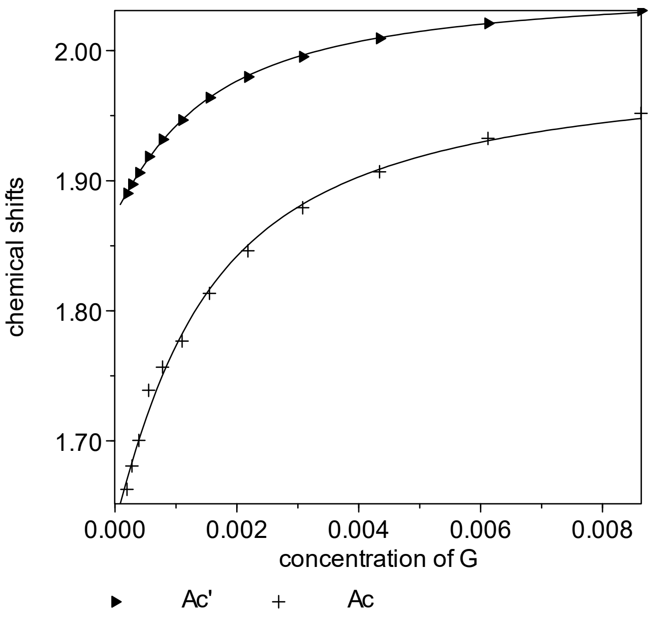
2.00
1.90
1.80
1.70
chemical shifts
0.000
0.002
0.004
0.006
0.008
concentration of G
Ac'
Ac

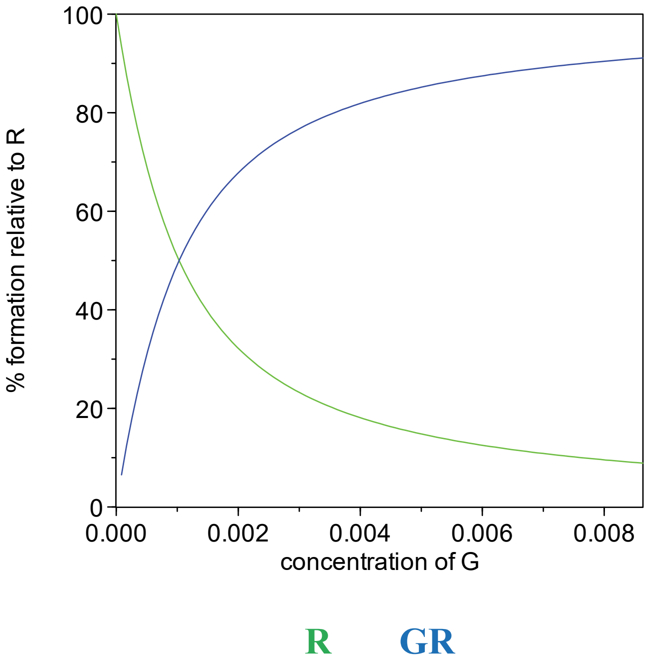
100
80
60
40
20
0
% formation relative to R
0.000
0.002
0.004
0.006
0.008
concentration of G
R
GR

3 + MeβGlcNAc$_2$ (D_2O, pD 7.4, 298 K, 500 MHz).

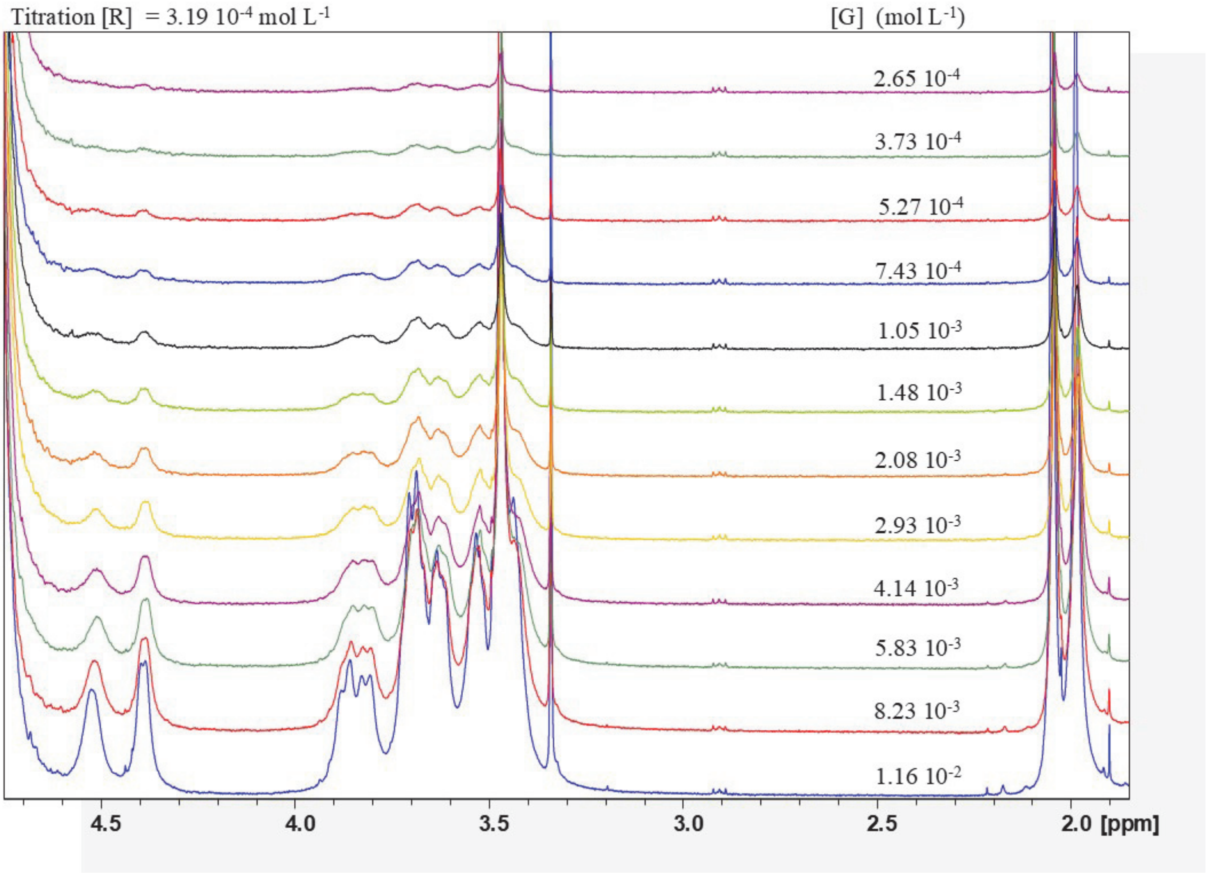

Figure S32. ^{1}H NMR spectroscopic titration (500 MHz, D_2O, pD7.4, 298 K) of receptor **3** ($3.19\ 10^{-4}$ mol L^{-1}) with incremental concentrations of MeβGlcNAc$_2$ (G).

Calorimetric titrations and data analysis.

Isothermal Titration Microcalorimetry experiments were performed at 298 K with a Nano-ITC instrument. After an initial injection of 3 μL, which was excluded from data analysis. aliquots of the titrant solution, containing the glycoside, were injected stepwise into the sample cell containing a solution of the titrate **3**. Solutions containing **3** were prepared in H_2O adjusting the pH to a value of 7.4 or 11. All experiments were performed in H_2O at pH 7.4 and 11. Heats of dilution were measured by injecting the titrant solution into neat H_2O and then subtracted from the binding heats. Data from titrations were fitted to measure the cumulative association constants and the thermodynamic parameters using the HypCal software package.[34]

3 + MeβGlcNAc₂ (H_2O, pH 11, 298 K).

Data Table

R = **3**   G = MeβGlcNAc2

[G] = 1.02 10^{-2} mol L^{-1}

Titration: [R] = 4.95 10^{-4} mol L^{-1}

Injection	Q (μJ)	Corrected Q (μJ)	inj volume (μL)	mol G (mol)	mol R (mol)	mol G / mol R	total volume (μL)
1	-23.30586167	-20.15525733	3	3.06E-08	4.66E-07	0.065624397	945
2	-538.015225	-461.642875	20	2.34E-07	4.56E-07	0.512579748	945
3	-451.97765	-383.8181	20	4.33E-07	4.47E-07	0.969198999	945
4	-385.122	-313.36955	20	6.28E-07	4.37E-07	1.435691099	945
5	-350.410475	-278.996375	20	8.19E-07	4.28E-07	1.912269514	945
6	-301.470775	-230.50805	20	1.01E-06	4.19E-07	2.399152327	945
7	-272.202375	-203.59605	20	1.19E-06	4.10E-07	2.896562337	945
8	-247.79385	-178.00565	20	1.37E-06	4.01E-07	3.404727157	945
9	-225.33725	-156.006475	20	1.54E-06	3.93E-07	3.923879325	945
10	-204.91395	-138.69545	20	1.71E-06	3.85E-07	4.454256405	945
11	-192.29765	-127.610775	20	1.88E-06	3.76E-07	4.996101097	945
12	-179.99095	-115.4682	20	2.05E-06	3.69E-07	5.54966135	945
13	-168.5206	-106.3197	20	2.21E-06	3.61E-07	6.115190473	945

Results page

```
 Reagent Reagent
  number   name
    1      R
    2      G

 Temperature: 25.000 Celsius. Excessive limit 0.99

   sigma =   0.00625

 Formation constants   Value    relative    log    standard
                                std devn    beta   deviation
 Beta A refined     0.6098E+03  0.0343    2.7852    0.0149    1  1


 Formation entalpies    Value   standard
                                deviation
-DeltaH A refined      10.6159   0.2019


+++++++++++++++++++++++++++++++++++++++++++++++++++++++++++++++++++++++++++++++
 Thermodynamic Functions, kJ/mol


              - DeltaG°                - DeltaH°                 T DeltaS°
   A        15.8978    0.0849        10.6159    0.2019         5.2820    0.2823


+++++++++++++++++++++++++++++++++++++++++++++++++++++++++++++++++++++++++++++++

 Correlation coefficients*1000         Run timed at 09.45 on  4 Sep 2025

   2 -924
       1

 Order of parameters:
   1       Beta   A
   2      -DeltaH A
```

Results table

Addition (µL)	Qobs (mJ)	Qcalc (mJ)	residual (mJ)	QTobs (mJ)	QTcalc (mJ)
0.87	-0.0202				
20.87	-0.4616	-0.4539	-7.7000e-3	-0.4818	-0.4740
40.87	-0.3838	-0.3831	-7.0000e-4	-0.8656	-0.8572
60.87	-0.3134	-0.3249	0.0116	-1.1790	-1.1821
80.87	-0.2790	-0.2772	-1.8000e-3	-1.4580	-1.4593
100.87	-0.2305	-0.2381	7.6000e-3	-1.6885	-1.6974
120.87	-0.2036	-0.2058	2.3000e-3	-1.8921	-1.9032
140.87	-0.1780	-0.1792	1.2000e-3	-2.0701	-2.0824
160.87	-0.1560	-0.1570	1.0000e-3	-2.2261	-2.2394
180.87	-0.1387	-0.1384	-3.0000e-4	-2.3648	-2.3778
200.87	-0.1276	-0.1227	-4.9000e-3	-2.4924	-2.5006
220.87	-0.1155	-0.1094	-6.0000e-3	-2.6079	-2.6100
240.87	-0.1063	-0.0981	-8.3000e-3	-2.7142	-2.7081

Titration Plots

Experimental (symbols) and calculated (cross and lines) heats

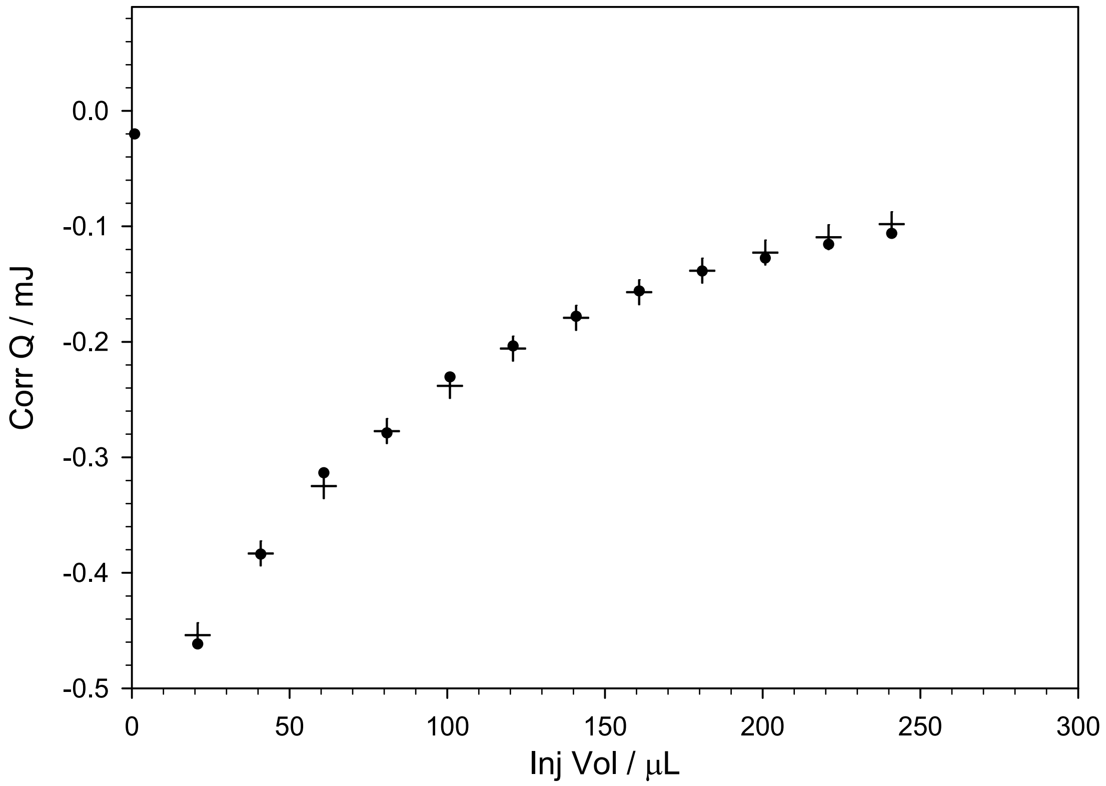

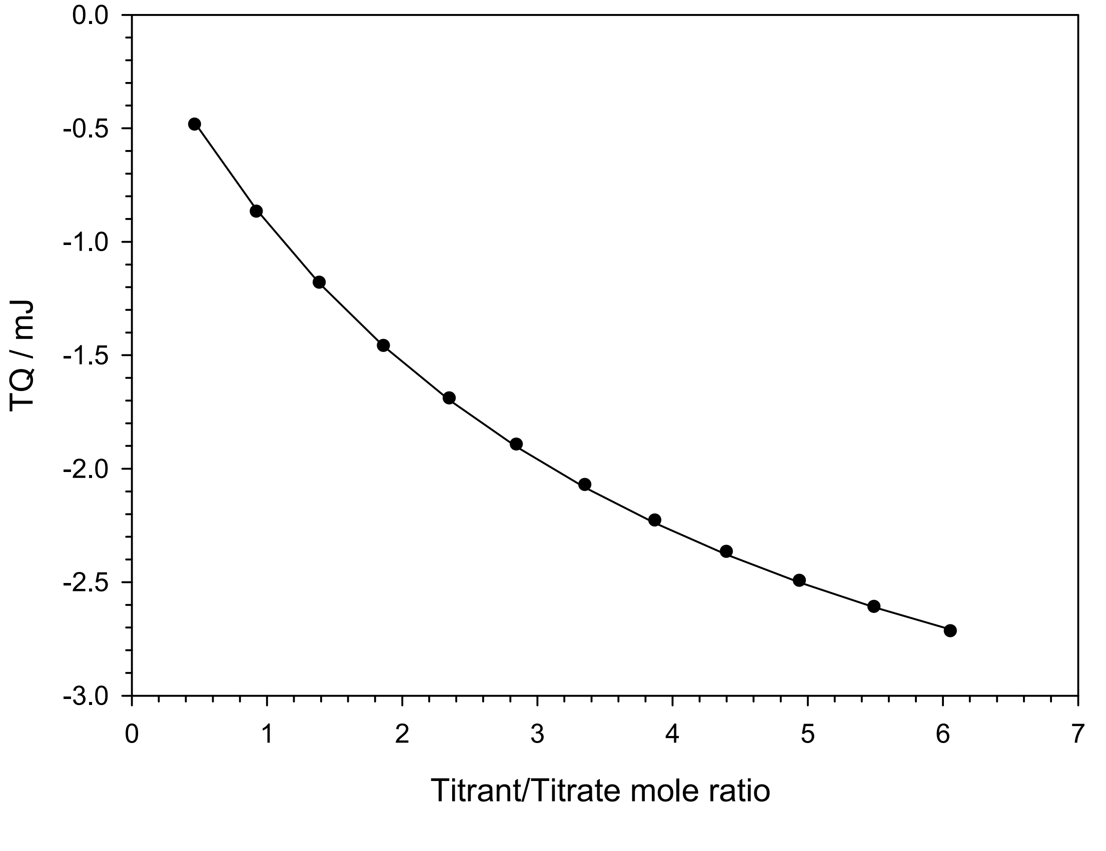

3 + MeβGlcNAc$_2$ (H_2O, pH 7.4, 298 K).

Data Table

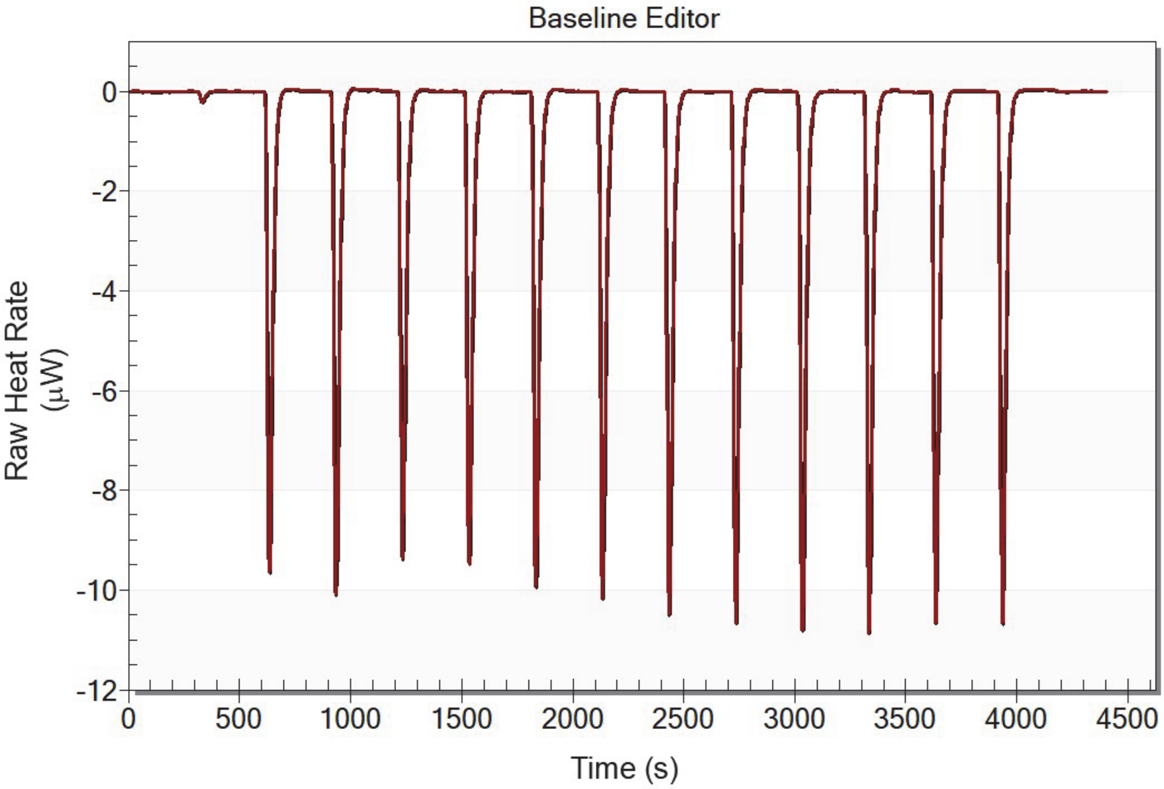

Figure S33. ITC titration (H_2O, pH 7.4, 298 K) of receptor **3** (4.95 10^{-4} mol L^{-1}) with incremental injection (3 μL first, then 12 x 20 μL) of MeβGlcNAc$_2$ (1.02 10^{-2} mol L^{-1}).

Structural studies.

NMR studies.

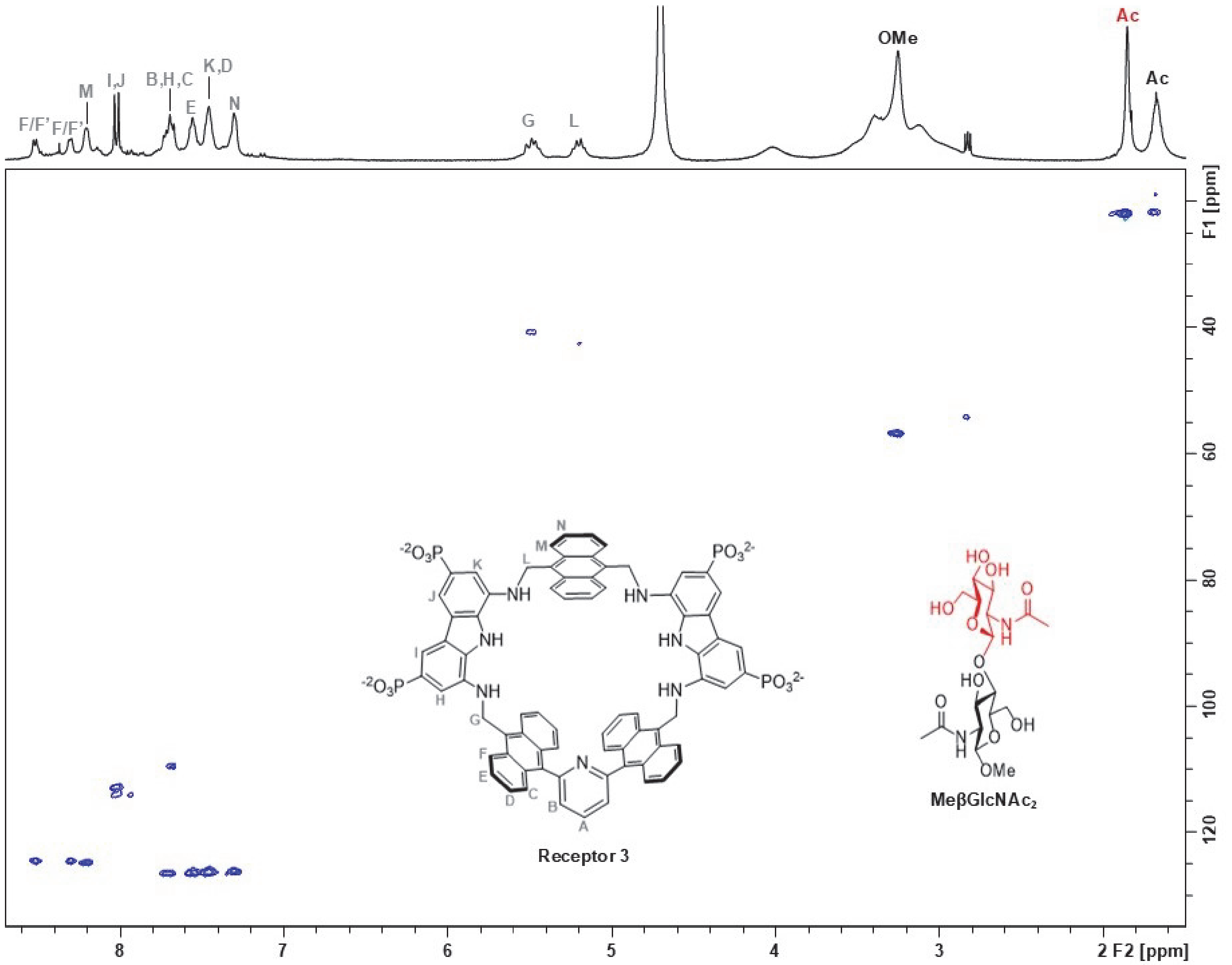

Figure S34. HSQC (500 MHz) spetrum of a mixture of **3** (1 mM) and MeβGlcNAc2 (3 mM). Assigned protons are labelled.

In Silico Docking Study. Initial structures of MeβGlcNAc2 and receptor **3** (in its tetraphosphonate anionic form as expected at pH 11) were built and minimized using conjugate gradients with the OPLS_2005 force field, Generalized Born/Surface Area (GB/SA) continuum solvation model with water set as solvent and an extended cutoff used to treat remote interactions. A maximum number of 5000 iterations were employed with the Polak-Ribiere Conjugate Gradient (PRCG) scheme, until the convergence energy threshold was 0.05. The MeβGlcNAc2 was manually docked within the receptor cleft with different starting relative orientations and further minimized. Minimization results afford different structures which were employed as input for conformational search using a Monte Carlo torsional sampling method (MCMM) with automatic setup during the calculation, energy window of 21 kJ mol-1, 10000 maximum number of steps, and 100 steps per torsion of the bond to be rotate, without any constraint. Several complexes were found to be stable, in which the sugar was located inside the receptor cleft. The protocol returned a family of structures, containing the minimum energy structure of the conformational search.

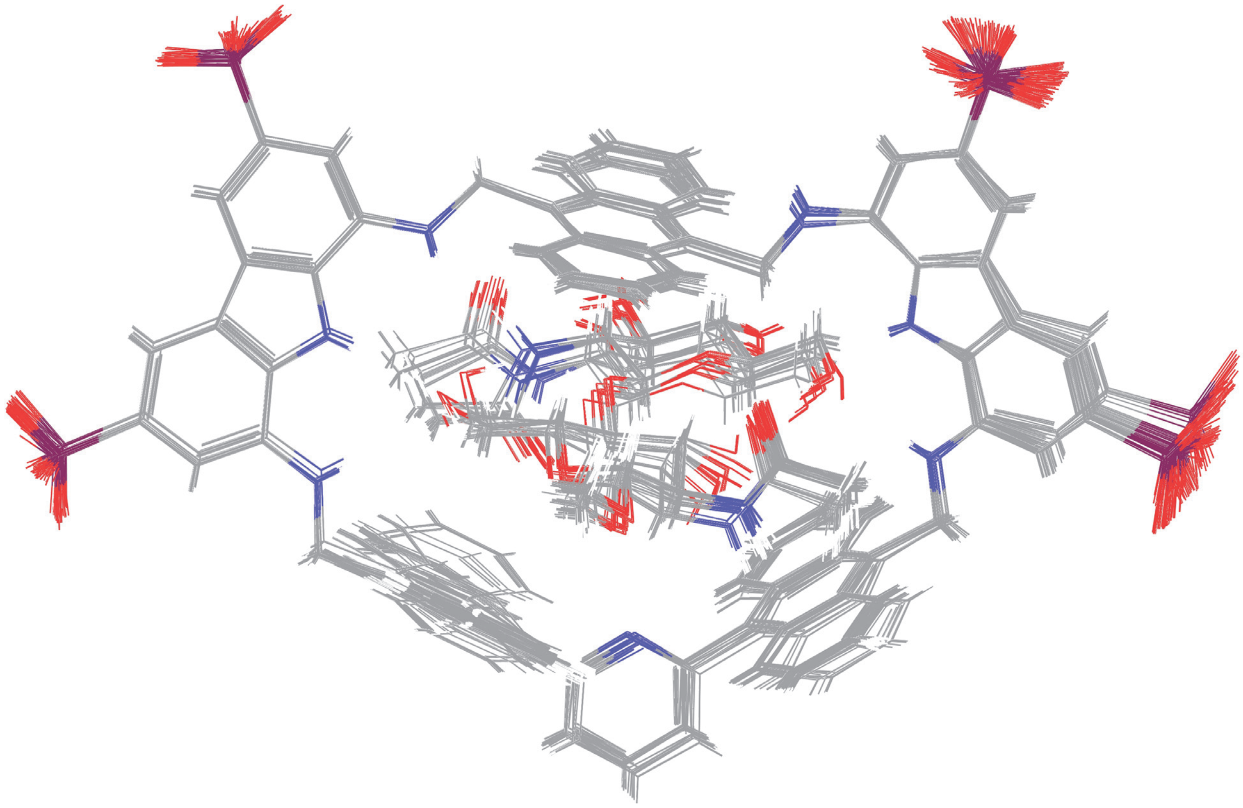

Figure S35. Molecular modelling results from the conformational search on the 1:1 complex between **3** and MeβGlcNAc2. Superposition of the 112 minimum energy structures, within an energy window of 10.0 kJ mol^{-1}, identified among the 744 structures obtained from the calculation.
